# Transcription-independent induction of rapid-onset senescence is integral to healing

**DOI:** 10.1038/s41556-026-01948-2

**Published:** 2026-05-28

**Authors:** Karla Valdivieso, Tomaz Rozmaric, Stella Victorelli, Vaibhav Jadhav, Nadja Anneliese Ruth Ring, Sylwia Machcińska-Zielińska, Barbara Schädl, Helene Dworak, Eirini Klinaki, Ines Fischer, Agnieszka Gadecka, Iryna Moskalevska, Sara Kostrebic, Michaela Kienberger, Edyta Marzec, Christina Efraimoglou, Alessia Zanchetta, Julien Cherfils-Vicini, Oleh Lushchak, Andreas Löscher, Thomas Kolbe, Maik Dahlhoff, Nicholas E. Pirius, Aniko Gutasi, Sarolta Takacs, James Ferguson, Bruno K. Podesser, Paul Slezak, David Monroe, Bin Zhou, Sundeep Khosla, Johannes Grillari, Heinz Redl, Diana Jurk, Mikolaj Ogrodnik

**Affiliations:** 1 Ludwig Boltzmann Research Group Senescence and Healing of Wounds, Vienna, Austria; 2 Ludwig Boltzmann Institute for Traumatology in AUVA Research Center, Vienna, Austria; 3 Austrian Cluster for Tissue Regeneration, Vienna, Austria; 4 Institute of Molecular Biotechnology, University of Natural Resources and Life Sciences, Vienna, Austria; 5 Department of Physiology and Biomedical Engineering and Robert and Arlene Kogod Center on Aging, Mayo Clinic, Rochester, MN USA; 6 InLife Institute of Animal Reproduction and Food Research, Polish Academy of Sciences, Regenerative Biology Team, Olsztyn, Poland; 7 University Clinic of Dentistry, Medical University of Vienna, Vienna, Austria; 8 Université Côte d’Azur, Centre National de la Recherche Scientifique (CNRS) UMR7284, Institut National de la Santé et de la Recherche Médicale (INSERM) U1081, Adipo-Cible Research Study Group, Institute for Research on Cancer and Aging, Nice (IRCAN), Nice, France; 9 Institut Hospitalo-Universitaire (IHU) RESPIRera and FHU OncoAge, CHU, Nice, France; 10 Kyiv School of Economics, Kyiv, Ukraine; 11 Research and Development University, Ivano-Frankivsk, Ukraine; 12 Institute of In Vivo and In Vitro Models, University of Veterinary Medicine Vienna, Vienna, Austria; 13 Department of Agricultural Sciences, University of Natural Resources and Life Sciences, Vienna, Austria; 14 Center for Biomedical Research and Translational Surgery, Medical University of Vienna, Vienna, Austria; 15 New Cornerstone Science Laboratory, State Key Laboratory of Cell Biology, Shanghai Institute of Biochemistry and Cell Biology, Center for Excellence in Molecular Cell Science, Chinese Academy of Sciences, University of Chinese Academy of Sciences, Shanghai, China

**Keywords:** Senescence, Cell growth, Ageing

## Abstract

Cellular senescence plays key roles in tissue repair, tumour suppression and ageing. Here we identify a rapid, transcription‑independent senescence response in skin following injury. Within minutes to hours after wounding, skin cells at the edge of injury display hallmark features of senescence. This response involves the utilization of pre‑existing *Cdkn1a* mRNA through the removal of nuclear export inhibitors, which enables *Cdkn1a* transcript translation and rapid p21 protein accumulation. These cells enter stable cell‑cycle arrest and secrete pro‑migratory and pro‑inflammatory factors that promote tissue repair, including re‑epithelialization. Experimental suppression of this rapid senescence, either genetically or pharmacologically, markedly delays wound closure, whereas inhibition during later phases of repair has no effect. Our findings establish rapid‑onset senescence as a mechanistic requirement for efficient tissue regeneration.

## Abstract (web summary)

Valdivieso, Rozmaric et al. show that wounding rapidly induces a senescent state in skin cells involving p21 protein induction, without the need for new transcription. These senescent cells are beneficial to early stages of healing.

## Main

Cellular senescence is defined as a state of cells initiated with cell cycle arrest and entails changes on the levels of the transcriptome, proteome, secretome and others^1,2^. Senescent cells can be distinguished by a combination of markers, including cell cycle inhibitors: p21^CIP1/WAF1^ protein (hereafter referred to as p21), which is encoded by *Cdkn1a* gene (whose transcript/messenger (mRNA) is also denoted as *Cdkn1a* mRNA) and p16^INK4A^ (*Cdkn2a* gene)^2,3^, reduction in Lamin B1 (LMNB1)^4^, senescence-associated secretory phenotype (SASP)^5^, DNA damage^6,7^ and accumulation of lipids in senescence (ALISE) phenotype^8,9^. Senescent cells accumulate during ageing and have been causally implicated in pathologies of many age-related diseases^10^. Despite the synonymous meaning of ‘senescent’ and ‘old’, senescent cells are found not only in ageing organisms but also in wounds^11–14^. While eliminating p16^+^ cells has been shown to delay skin wound healing in healthy mice^12^, it remains unclear how the timing and mechanism of senescence induction affects healing.

The decisive stage of the response of skin to wounding is the immediate recruitment of its cells to guide the healing process^15^. Our recent findings indicate that signalling pathways become active within minutes following skin injury and exhibit spatiotemporal control that shapes the healing process^13^. It is thus reasonable to expect that additional processes are induced just as quickly in the skin, as a rapid response to damage. While senescence has been shown to be integral to the wound response^12,13,16^, the current paradigm for this field holds that cellular senescence requires at least several days from the time of insult to develop^1,12,17^. However, these results are based primarily on experiments introducing damaging stimuli, such as in cultured human fibroblasts in vitro and in animal models or preneoplastic conditions^18^, as well as treated with doxorubicin or subjected to X-ray^1,13,17^, and the timing necessary for induction of senescence in physiological processes in vivo is unknown. The question on the temporal dynamics of cellular senescence in a physiological context stands as a challenge and needs to be addressed for the field to understand what senescent cells are and what they do in vivo.

Assessing how senescence induction occurs in wounds, we discovered that p21^+^ senescent cells appear as early as 90 min after injury. Utilizing p21-lineage-tracing mice, we here show that senescence induction is persistent and that senescent cells are removed after injury is healed. Rapid senescence induction is transcription-independent, with high basal levels of *Cdkn1a* transcripts present in homeostatic skin, allowing these cells to produce p21 protein immediately upon injury. Mechanistically, *Cdkn1a* transcripts lose proteins that are known to restrict their nuclear export, which in turn permits interaction with translation initiation factors and promotes translation. Notably, interfering with the senescence machinery or eliminating senescent cells impairs wound healing, but only if carried out during the early stage of the healing process. Thus, our data support the concept that the rapid onset and transcription-independent induction of senescence is pivotal for the healing process, and its modulation might provide new therapeutic avenues.

Throughout the research project, we adhered to the Guidelines for Minimal Information on Cellular Senescence Experimentation in vivo (MICSE)^2^ to ensure a high level of reliability in identifying and analysing senescent cells.

## Results

### p21^+^ cells present a robust response to injury in vivo

**Fig. 1:**
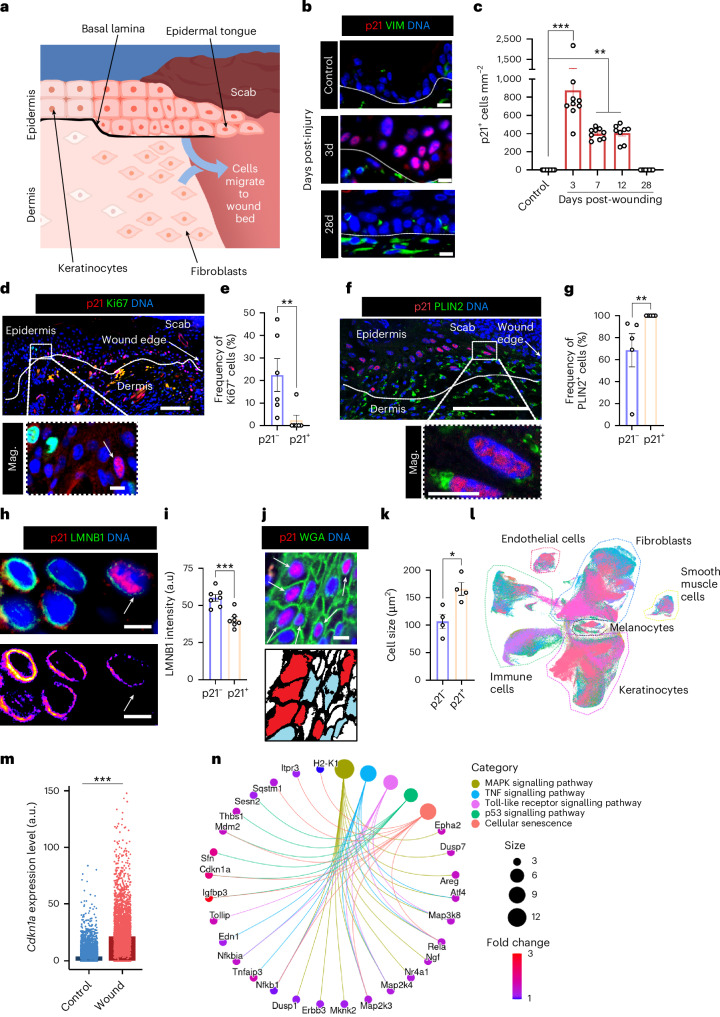
p21^+^ cells present a robust senescence response to injury in vivo. **a**, A schematic of skin regions and cell types involved in the process of healing. **b**, Representative images of p21 (red) and vimentin (VIM, green) in samples collected from control (homeostatic) mouse skin as well as from skin 3 and 28 days post-injury. **c**, Quantification of the number of p21^+^ keratinocytes per mm^2^ of epidermis proximal to the wound. *P* values are control versus 3 days (<0.0001), control versus 7 days (0.0042), control versus 12 days (0.0047) and control versus 28 days (>0.9999). **d**, Representative images of p21 (red) and Ki67 (green) staining in mouse skin 3 days after wounding. White arrow marks a p21^+^ keratinocyte. **e**, Quantification of the frequencies of Ki67^+^ nuclei within populations of p21^−^ and p21^+^ stratum basale keratinocytes. *P* value is 0.0087. **f**, Representative images of the staining against Perilipin 2 (PLIN2, green) and p21 (red). **g**, Quantification of the frequency of keratinocytes positive for PLIN2 and p21. *P* value is 0.0079. **h**, Murine skin stained for Lamin B1 (LMNB1, green) and p21 (red). The image below shows a heatmap of LMNB1 signal intensity. White arrow marks a p21^+^ keratinocyte. **i**, Quantification of LMNB1 in p21^+^ and p21^−^ keratinocytes within the close proximity to the 3-day mouse wound. *P* value is 0.0004. **j**, Representative images of staining against (top) cell outline (with wheatgerm agglutinin (WGA), green) and p21 (red), and the mask of the WGA channel, colour-mapped for p21^+^ and p21^−^ (with visible nuclei) keratinocytes. **k**, Size quantification of p21^+^ and p21^−^ keratinocytes within the close proximity to the 3-day mouse wound. *P* value is 0.0146. **l**, Uniform Manifold Approximation and Projection (UMAP) of ~400,000 cells used to create the mouse skin cell atlas. Populations of cells residing within skin were identified and are outlined with dashed lines. **m**, Average expression levels of *Cdkn1a* among keratinocytes from samples of control (homeostatic) and wounded mouse skin. Each dot represents an individual cell, and the average expression level of all cells is shown as a bar graph. *P* value is <0.0001. **n**, Category-gene-net plot of differentially expressed genes (DEGs) between *Cdkn1a*^*+*^ and *Cdkn1a*^−^ keratinocytes with upregulated pathways listed on the right side. In all the images DNA is stained with 4,6-diamidino-2-phenylindole (DAPI) (blue) and white lines mark the basal lamina of epidermis. Data are from *n* = 8–9 mice per group for **c**; *n* = 5 mice per group for **e**,**g**; *n* = 6–7 mice per group for **i** and *n* = 4 mice for **k**. Mean ± s.e.m. is plotted. For **c**, a one-way analysis of variance (ANOVA) with post hoc Dunnet’s test (two-sided) was used. For **e**,**i**,**k** an unpaired *t*-test (two-sided) was used and for **g**, a Mann–Whitney *U*-test (two-sided) were used. *P < 0.05, **P < 0.01, ***P < 0.001. The scale bars for all overview images are 100 μm and for magnifications 10 μm. Source numerical data are available in source data. Source data

**Extended Data Fig. 1:**
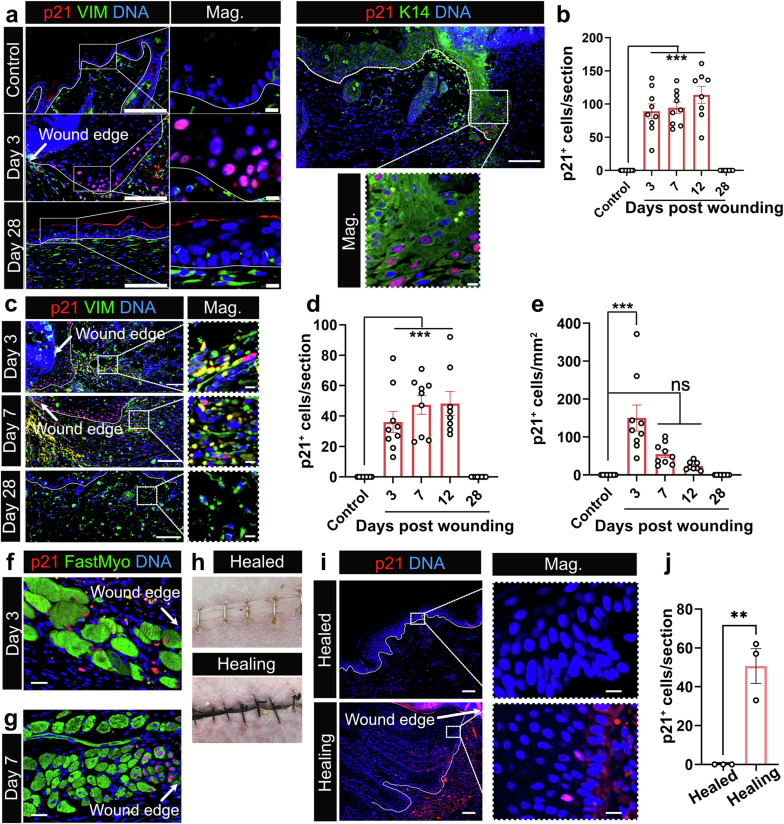
p21^+^ cells present a robust senescence response to injury in vivo. **(a) On the left side:** Representative images of p21 (red) and vimentin (VIM, green) in samples collected from control mouse skin as well as 3 and 28 days post-injury. **On the right side**: A representative image of p21 (red) and Keratin 14 (K14; green) in mouse skin injury 3 days after wounding. **(b)** Quantification of the number of p21^+^ keratinocytes per histological section. P values are: Control vs. 3 days (<0,0001), Control vs. 7 days (<0,0001), Control vs. 12 days (<0,0001), Control vs. 28 days (>0,9999). **(c)** Representative images of p21 (red) and VIM (green) in dermis of samples collected from mouse skin 3, 12 and 28 days post-injury. **(d)** Quantification of the number of p21^+^ fibroblasts per histological section. P values are: Control vs. 3 days (<0,0001), Control vs. 7 days (<0,0001), Control vs. 12 days (<0,0001), Control vs. 28 days (>0,9999). **(e)** Quantification of the number of p21^+^ fibroblasts per mm^2^ of dermis proximal to injury. P values are: Control vs. 3 days (<0,0001), Control vs. 7 days (0,0667), Control vs. 12 days (0,7165), Control vs. 28 days (>0,9999). Representative images of p21 (red) and Fast Myosin (green) showing p21^+^ myocytes in *panniculus carnosus* at **(f)** day 3 (3d) and **(g)** 7 d after injury. **(h)** Representative photos of porcine incision injuries 7 d after wounding. **(i)** Representative images of staining against p21 (red) in the epidermis of healed and healing porcine skin. **(j)** Quantification of the number of p21^+^ keratinocytes per histological section of the porcine incision injuries. P value is 0,0048. In all images DNA is stained with DAPI (blue) and white lines mark the basal lamina of epidermis. Data are from n = 8–9 mice per group for (**b**), (**d**) and (**e**), n = 3 pigs per group for (**j**). Mean ± SEM plotted. For (**b**), (**d**) and (**e**) one-way ANOVA with post-hoc Tukey’s test was used. For (**j**) an unpaired t-test was used. **p < 0.01, ***p < 0.001. The scale bars for (**a**), (**c**), (**f**), (**g**) and (**i**) images are 100 μm and their magnifications 10 μm. Source numerical data are available in source data.

Healing involves an interplay between different structures, including epidermis and dermis, their predominant types of cells such as keratinocytes and fibroblasts, which are all involved in migration and re-populating the injury site (Fig. 1a and ref. ^15^). To investigate the role of senescence in healing we made full-thickness excision wounds in mice and collected samples at days 3, 7, 12 and 28 post-wounding. We found that a large number of cells stained positive for p21 protein (which we refer to as p21^+^ cells in the manuscript), are present throughout the process of healing (Fig. 1b, c and overview in Extended Data Fig. 1a,c) in all compartments of the skin. Various skin cell types become p21^+^ upon injury, including dermal fibroblasts (Extended Data Fig. 1c–e), keratinocytes (Fig. 1b,c and overview in Extended Data Fig. 1a) and myocytes of the underlying panniculus carnosus (Extended Data Fig. 1f,g), among others. While the total number of p21^+^ cells remains constant throughout the process of healing (Extended Data Fig. 1b,d), the concentration of p21^+^ keratinocytes and fibroblasts per mm^2^ decreases as the volume of skin involved in healing and number of cells within the regrowing skin increase (Fig. 1c and Extended Data Fig. 1e). We found that among these time points the highest concentration of p21^+^ keratinocytes and fibroblasts can be observed at 3 days post-wounding (Fig. 1c and Extended Data Fig. 1e). Of note, there are no p21^+^ cells in homeostatic, unwounded skin nor are there any after wound closure (skin was collected 4 weeks after wounding, which corresponds to about 2 weeks after wound closure) (Fig. 1b,c and Extended Data Fig. 1a–e). The wounding-associated increase in the number of p21^+^ cells in mice was confirmed in porcine skin 7 days after wounding, where samples collected from healed wounds had a lower number of p21^+^ keratinocytes than samples collected from skin excised from wounds where healing was still ongoing (Extended Data Fig. 1h–j).

### p21^+^ cells present a robust senescence phenotype in wounds

**Extended Data Fig. 2:**
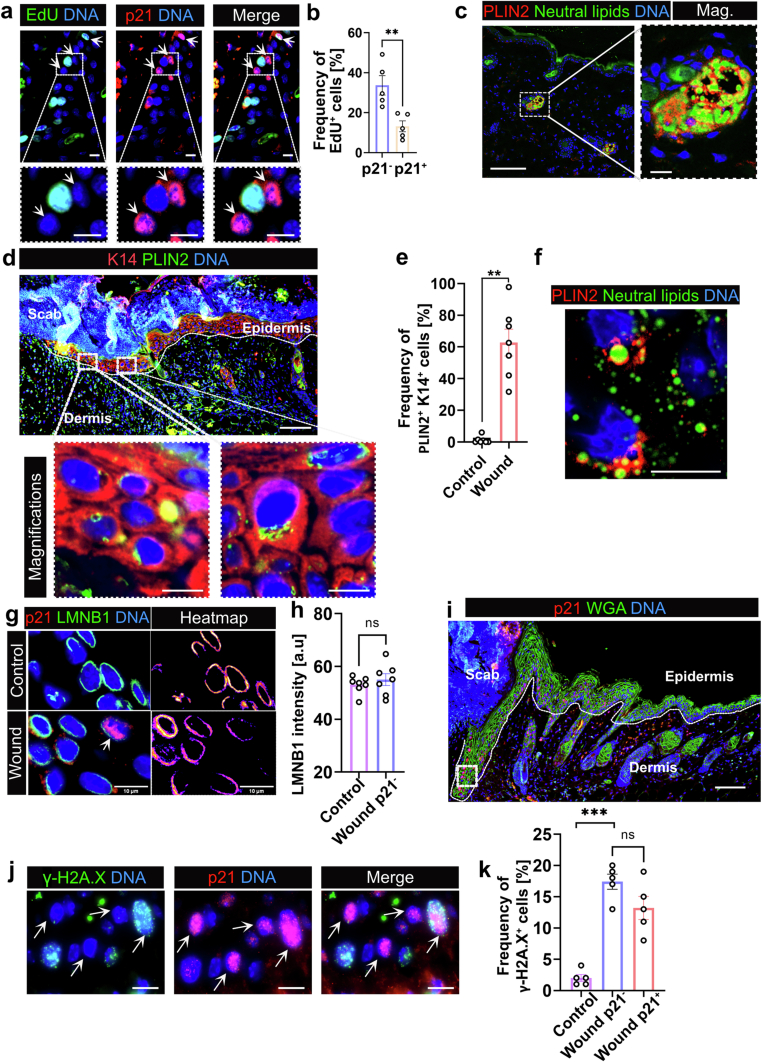
p21^+^ cells present a robust senescence response to injury in vivo. **(a)** Representative images of p21 (red) and 5-Ethynyl-2′-deoxyuridine (EdU, green) staining in mouse skin 3 days after wounding. White arrows mark cell positive for p21. **(b)** Quantification of the frequencies of EdU^+^ nuclei within populations of p21^−^ and p21^+^ *stratum basale* keratinocytes. P value is 0,0062. **(c)** A representative image of a sebaceous gland in mouse control skin stained for Plin2 (red) and neutral lipids (green). White arrow marks a cell positive for p21. **(d)** A representative image of mouse skin proximal to a day 3 (3 d) excision wound and stained for PLIN2 (green) and keratin 14 (K14, red). **(e)** Quantification of the frequency of PLIN2^+^ lipid droplets in (K14^+^) keratinocytes at the wound site and in the control mouse skin. P value is <0,0001. **(f)** A representative image of mouse keratinocytes proximal to the injury site and positive for PLIN2 (red) and neutral lipids (green). **(g)** Epidermis of wounded murine skin stained for Lamin B1 (LMNB1, green) and p21 (red). White arrow marks a cell positive for p21. Panel on the right shows LMNB1 intensity as a heatmap of signal originating from the antibody. White arrows mark a cell positive for p21. (**h**) Quantification of LMNB1 in control keratinocytes (of unwounded skin) and in keratinocytes negative for p21 within the close proximity to the 3 d mouse wound. **(i)** A representative image of mouse skin adjacent to the 3 d wound and stained for Wheat Germ Agglutinin (WGA, green) and p21 (red). The dashed rectangle shows which region was magnified for the representative image on the Fig. 1. (**j**) Representative images of p21 (red) and γ-H2A.X (green) staining in mouse skin 3 d after wounding. White arrows mark p21-positive cells. (**k**) Quantification of the frequency of cells positive for high number (≥10) of γ-H2A.X foci in control epidermis and in epidermis from samples in close proximity to the 3 d mouse wounds. P values are <0,0001 (control vs. Wound p21^−^), 0,0002 (control vs. p21^+^) and 0,1017 (wound p21^−^ vs. wound p21^+^). For all images DNA is stained with DAPI (blue) and epidermis is outlined in white. Data are from n = 5 mice per group for (**b**) and (**k**) and from n = 6–7 mice per group for (**e**) and (**h**). Mean ± SEM plotted. For (**b**), (**e**) and (**h**) an unpaired t-test (two-sided) was used. For (**k**) one-way ANOVA with post-hoc Tukey’s test (two-sided) was used. ** p < 0.01 ***p < 0.001 and, ns’ stands for, non-significant’. Scale bars show 10 μm for (**a**), (**g**), (**g**) and (**j**), while for (**c**), (**d**) and (**i**) they show 100 μm and 10 μm for low and high magnifications respectively. Source numerical data are available in source data.

In the field of cellular senescence, it is considered essential to apply multiple markers of senescence to determine whether a cell is ‘bona fide’ senescent^2^. One of the core features of senescent cells is a reduction in their proliferation, that is, the cell cycle arrest^1,2^. To confirm that p21 expression in this context was indicative of a cell cycle arrest, we co-stained for Ki67, a marker for proliferation, resulting in almost fully exclusive positivity for either p21 or Ki67, suggesting that p21^+^ are not dividing (Fig. 1d,e). To further verify this, we treated mice with the thymidine analogue 5-ethynyl-2′-deoxyuridine (EdU), which is incorporated into DNA during proliferation. The subsequent analysis revealed that p21^+^ keratinocytes are indeed significantly less frequently positive for EdU (Extended Data Fig. 2a,b). Perilipin 2 (PLIN2) is found on the surface of lipid droplets and its detection can be used as a reliable way to quantify lipid droplets in histology^8,19^. A phenotype of ectopic accumulation of lipid droplets, known as the ALISE phenotype, has recently been shown by us^8,9^ and others^20^ to be associated with cellular senescence in situ. Accordingly, while PLIN2^+^ vesicles in homeostatic skin are present solely in the sebaceous glands (Extended Data Fig. 2c), PLIN2^+^ vesicles are found in high number at the injury site and associated with p21^+^ cells of several types, including keratinocytes (Fig. 1f,g and Extended Data Fig. 2d,e). To verify that PLIN2 indeed stains lipid droplets, we used a dye binding neutral lipids and showed that PLIN2^+^ vesicles are also positive for neutral lipids in the sebaceous glands (Extended Data Fig. 2c) and at the wound site (Extended Data Fig. 2f). This represents emerging evidence that lipid droplets of skin cells may contribute to the skin healing process in situ. Another robust marker of senescence is the reduction of Lamin B1 (LMNB1)^4^, and in our wound model p21^+^ keratinocytes show a distinct reduction in the level of LMNB1 (Fig. 1h,i). In contrast, the LMNB1 levels between p21^−^ keratinocytes of wounded and control skin remain unaltered (Extended Data Fig. 2g,h shows a larger overview of the same region, with more p21^−^ keratinocytes). Moreover, senescence is often associated with cell hypertrophy^21,22^, and indeed, we found that p21^+^ keratinocytes are on average of larger soma size (Fig. 1j,k and Extended Data Fig. 2i shows a larger overview). Notably, although keratinocytes at the wound site show an overall higher level of DNA double-stranded breaks, there was no difference between p21^+^ and p21^−^ keratinocytes (Extended Data Fig. 2j,k). Overall, our results indicate that a high level of p21 protein is associated with the presence of five additional markers of cellular senescence.

### Single-cell RNA-seq atlas of mouse skin confirms *Cdkn1a*-associated senescence in wounds

**Extended Data Fig. 3:**
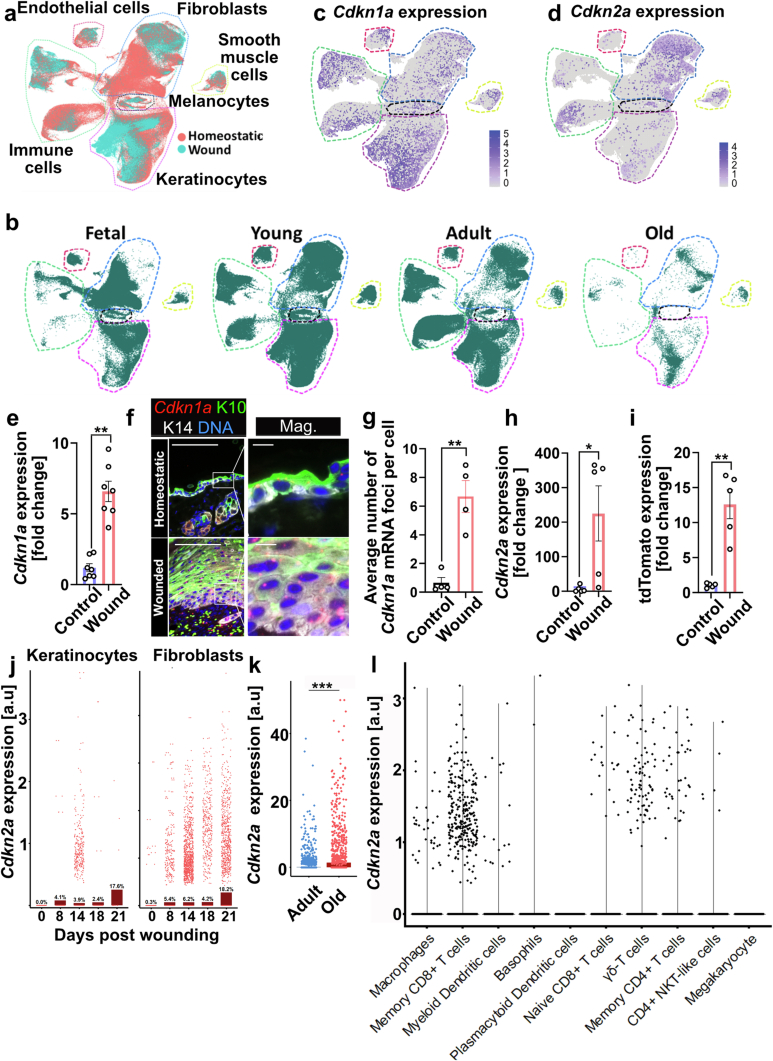
Single-cell RNA-seq atlas of mouse skin cells confirms *Cdkn1a*-associated senescence in wounds. **(a)** UMAP of mouse skin cell atlas showing cells originating from samples collected from wounds and from homeostatic (control) skin. Distinctive types of cells residing in mouse skin were labelled and outlined with dashed lines matching those of Fig. 1. **(b)** UMAPs of mouse skin cells showing which cells originate from samples collected from skin of animals belonging to different age groups: embryonic, young, adult and old. **(c)** UMAP showing expression level of *Cdkn1a* (p21) across populations of mouse skin cells. **(d)** UMAP showing expression level of *Cdkn2a* (p16) across populations of mouse skin cells. **(e)** RT-qPCR results of *Cdkn1a* expression at 7 days after wounding compared to unwounded mouse skin. P value is <0,0001. **(f)** Representative images of RNA *in situ* hybridization (RNA-ISH) against *Cdkn1a* mRNA (red foci) co-stained with a marker of *stratum basale*, keratin 14 (K14; grey) and a marker of *stratum spinosum*, keratin 10 (K10; green). DNA is stained with DAPI (blue). **(g)** Quantification of *Cdkn1a* mRNA foci in K14^+^ keratinocytes of the wounded and the control skin. P value is 0,0022. RT-qPCR results of **(h)** *Cdkn2a* (p16) (P value is 0,0159) and **(i)** *tdTomato* (P value is 0,0048) expression at 7 days after wounding of p16-tdTomato mice when compared to the unwounded skin of p16-tdTomato mice. (**j**) Average *Cdkn2a* expression levels among keratinocytes (left side) fibroblasts (right side) from samples of control and wounded mouse skin used to create the sc-RNA-seq mouse skin atlas. The wound samples were split to represent the time points at which the samples were collected. Each dot represents an individual cell and an average is shown as a bar-graph. Values above each bar show the % of population positive the presence of *Cdkn2a* transcript. (**k**) The average expression levels of *Cdkn2a* among fibroblasts from skin samples from adult and old mice. Each dot represents an individual cell, and the average expression level of all cells is shown as a bar-graph. P value is <0,0001. (**l**) An average expression levels of *Cdkn2a* among various types of immune cells from the mouse skin cell atlas. Data are from n = 7 mice per group for graphs (**e**), for n = 4 mice per group for the graph (**g**) and n = 5 per group for graphs (**h**) and (**i**). Mean ± SEM plotted. For the graphs (**e**), (**g**), (**j**) and (**k**) an unpaired *t*-test was used, for graph (**h**) Mann-Whitney U test (two-sided) was used and for (**i**) an unpaired *t*-test (two-sided) with Welch’s correction. *p < 0.05, **p < 0.01, ***p < 0.001 and ‘ns’ is ‘non-significant’. The scale bars for (**f**) show 100 and 10 μm for low and high-magnification images, respectively. Source numerical data are available in source data.

To independently and globally characterize the wound-induced, p21^+^ senescent cells, we utilized single-cell RNA sequencing (sc-RNA-seq). Specifically, we combined and harmonized 20 published datasets of mouse skin cells^23–42^ into a large sc-RNA-seq atlas of mouse skin cells (Fig. 1l, colours represent different studies). The atlas consists of 78 tissue samples, comprising 406,563 cells of different skin cell types including keratinocytes, fibroblasts, smooth muscle cells, immune cells and others (Fig. 1l). It contains samples from both wounded and homeostatic skin (Extended Data Fig. 3a) as well as samples collected from across the mouse lifespan (Extended Data Fig. 3b). Using this atlas, we were able to select cells highly expressing transcripts associated with cellular senescence, such as *Cdkn1a* (Extended Data Fig. 3c) and *Cdkn2a* (Extended Data Fig. 3d).

Leveraging the atlas, we confirmed that keratinocytes from wound samples show an elevated level of the *Cdkn1a* transcript (Fig. 1m). An increase of *Cdkn1a* transcripts in wounds was further corroborated by quantitative PCR with reverse transcription (RT–qPCR) on bulk tissue (Extended Data Fig. 3e) and on single-cell level in situ using RNA in situ hybridization (ISH) (Extended Data Fig. 3f,g). In contrast, while we confirmed by RT–qPCR an already published^12^ increase of *Cdkn2a* after wounding in bulk tissue (Extended Data Fig. 3h) as well as a corresponding increase in tdTomato (Extended Data Fig. 3i) using the reporter model of p16-tdTomato mice^43^, in our atlas, the *Cdkn2a* transcript was detected at very low level in keratinocytes and fibroblasts (Extended Data Fig. 3d). When the datasets composing the atlas were split into days from injury, certain amounts of *Cdkn2a* were found in keratinocytes and fibroblasts at late time points post-wounding (Extended Data Fig. 3j). Of note, the atlas was able to detect a more prominent increase in *Cdkn2a* transcript with age, when fibroblasts of adult and aged mice are compared (Extended Data Fig. 3k), indicating that the demonstrated low level of *Cdkn2a* in stromal cells of wounds is not due to an insufficient sensitivity of the method to detect the *Cdkn2a* transcript. The more prominent increase in the level of *Cdkn2a* by RT–qPCR in bulk homogenates, but not in stromal cells on single-cell level, could be explained by infiltration of immune cells highly positive for *Cdkn2a* transcripts, which we also detected in our atlas (Extended Data Fig. 3l). Based on these observations, we focused our efforts on the *Cdkn1a*^+^ population, and using the category-gene-net analysis, we found that the transcriptional profile of *Cdkn1a*^+^ keratinocytes is associated with cellular senescence and several signalling pathways such as Toll-like receptor, mitogen-activated protein kinases and nuclear factor (NF)-κB (Fig. 1n). These results are supported by published observations showing upregulation of these pathways in senescent cells^44,45^.

### Senescent cells in injuries are associated with migration and inflammation

**Fig. 2:**
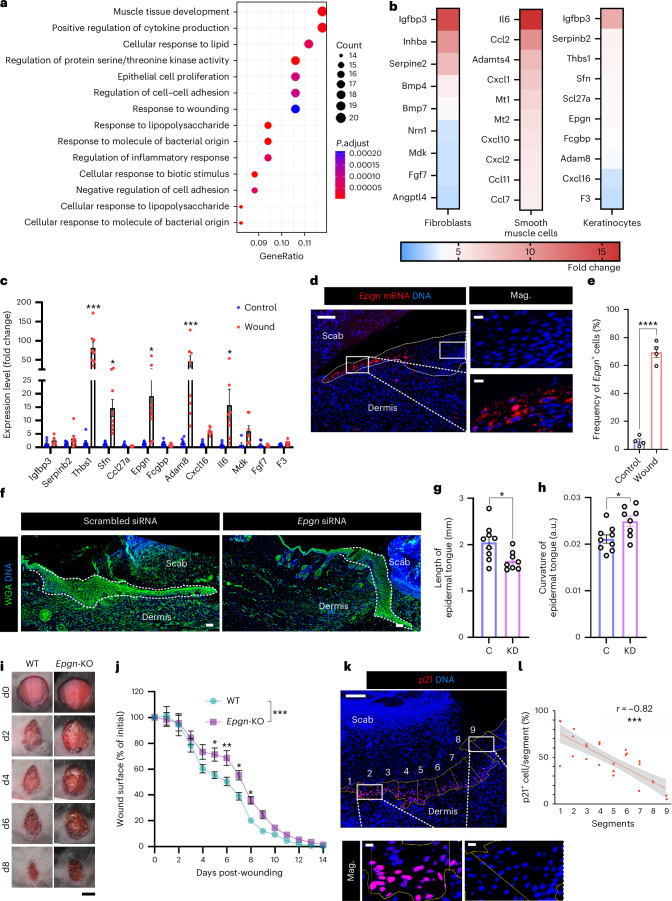
*Cdkn1a* and p21 are associated with inflammation and migration. **a**, Dot plot of GO enriched Kyoto Encyclopedia of Genes and Genomes (KEGG) terms of upregulated DEGs in the cluster of *Cdkn1a*^+^ keratinocytes compared with clusters of *Cdkn1a*^−^ keratinocytes. **b**, Heatmaps of expression of genes encoding for secretory factors, which have been found upregulated in the clusters of *Cdkn1a*^+^ fibroblasts, smooth muscle cells and keratinocytes. **c**, Results of RT–qPCR for genes selected from **b** and using 7 days (7 d) mouse wounds and control mouse skin. *P* values (for control versus wound) are 0.881931 (*Igfbp3*), 0.772919 (*Serpinb2*), <0.000001 (*Thbs1*), 0.049632 (*Sfn*), 0.944126 (*Ccl27a*), 0.009800 (*Epgn*), 0.951094 (*Fcgbp*), <0.000001 (*Adam8*), 0.483355 (*Cxcl16*), 0.038046 (*Il6*), 0.506247 (*Mdk*), 0.964925 (*Fgf7*) and 0.927831 (*F3*). **d**, A representative image of spatial distribution of *Epgn* mRNA (red) in the regrowing epidermis. **e**, Quantification of the % of *Epgn* mRNA^+^ keratinocytes in the epidermal tongue and the control region. *P* value is <0.0001. **f**, Representative images of WGA-stained mouse skin collected from mice treated with siRNA against *Epgn* or with scrambled siRNA. **g**, Quantification of the effects of *Epgn* silencing on the length of the epidermal tongue. *P* value is 0.0185. C, control; KD, knockdown. **h**, Quantification of the effects of *Epgn* silencing on the curvature of the epidermal tongue. *P* value is 0.0159. **i**,**j**, Representative photographs of wounds from wild-type (WT) or Epgn-KO B6.129-Epgn^tm1^ (Epgn-KO) mice (**i**) and its corresponding measurement of wound size (**j**) normalized to day 0. *P* values (for WT versus *Epgn*-KO) are >0.9999 (day 1), >0.9999 (day 2), 0.9927 (day 3), 0.2006 (day 4), 0.0403 (day 5), 0.0099 (day 6), 0.0339 (day 7), 0.0454 (day 8), 0.2321 (day 9), 0.9979 (day 10), 0.9995 (day 11), >0.9999 (day 12), >0.9999 (day 13) and >0.9999 (day 14). **k**, A representative image of spatial distribution of p21^+^ keratinocytes in epidermal tongue. In the image the fluorescence of the channel for p21 was removed outside the region of the epidermal tongue marked with the yellow line for clarity. Numbers 1–9 represent 100-μm-long segments that the tongue was divided into. Scab is marked above the epidermal tongue. Micrographs in the bottom panel show magnified regions of segments 2 and 9 of the epidermal tongue. The experiment was performed once with quantifications originating from three different mice. **l**, Quantification of the frequency of p21^+^ keratinocytes along the segments of the epidermal tongue. *P* value is <0.0001. In all images, DNA is stained with DAPI (blue). Data are from *n* = 6–8 mice per group for **c**, from *n* = 4 mice per group for **e**, from *n* = 8–9 for **g**,**h**,**l**, from *n* = 10 for **j** and *n* = 3 mice for **l**. Mean ± s.e.m. is plotted. For **c** an unpaired *t*-test (two-sided) normalized for multiple comparisons was used. For **e**,**g**,**h**, an unpaired *t*-test (two-sided) was used. For **j**, a two-way ANOVA with post hoc Sidak’s test (two-sided) was used. For **l**, Pearson’s correlation test (two-sided) was used. The bubble plot (**a**) was generated using enrichment analysis with Enrichr via gseapy, a Python package. The analysis employed Fisher’s exact test for over-representation, using a one-sided (right-tailed) test to assess enrichment relative to the background. Multiple-testing correction was applied using the Benjamini–Hochberg false discovery rate (FDR) method across terms within each library. The scale bars for **d**,**f**,**i**,**k** show 100 μm and 10 μm for low and high-magnification images, respectively. The scale bars for **i** are 5 mm. *P < 0.05, **P < 0.01 and ***P < 0.001. Source numerical data are available in source data.

To investigate further the p21^+^ phenotype, we conducted a clustering of skin cell populations within our sc-RNA-seq atlas and searched for clusters enriched for *Cdkn1a* expression (Supplementary Fig. 1a–f). Among keratinocytes, two clusters stood out with particularly high *Cdkn1a* expression levels (clusters 7 and 14; Supplementary Fig. 1d), of which cluster 14 displayed a keratin profile matching that of a murine wound, with high keratin 14 (K14) expression (Supplementary Fig. 1g–k). Focusing on this cluster, the subsequent pathway-enrichment analysis revealed that the *Cdkn1a*^+^ keratinocytes show an upregulation of inflammation and a reduction in adhesion (Fig. 2a). Consistently, the top upregulated genes in *Cdkn1a*^+^ keratinocytes (Supplementary Table 1), fibroblasts (Supplementary Table 2) and smooth muscle cells (Supplementary Table 3) contained a number of secretory factors, including pro-inflammatory cytokines, matrix-remodelling enzymes and growth factors (Fig. 2b), many of which overlap with known SASP factors^5,9^. To validate our in silico analysis, we used RT–qPCR and confirmed the upregulation of several of these factors in murine injuries (Fig. 2c). We further verified this finding spatially by showing that one of the prominently upregulated factors, epigen (*Epgn*) is highly enriched in keratinocytes associated with injury (Fig. 2d,e). Moreover, to assess the importance of *Epgn* in healing we performed a local knockdown (KD) of this factor using siRNA resulting in a reduction in *Epgn* expression in wounds (Supplementary Fig. 1l,m). Consistently, reduction in *Epgn* impeded re-epithelialization and results in shorter parts of regrowing epidermis, ‘epidermal tongues’ (Fig. 2f,g), which have not yet extended underneath the scab, thus of higher curvature (Fig. 2f,h). Finally, we utilized Epgn-knockout (KO) B6.129-Epgn^tm1^ mice^46^, which do not show abnormal embryonic development or tissue homeostasis^46^, to test whether absence of EPGN impacts healing. Indeed, we found that the KO mice show a prominent reduction in wound healing rate (Fig. 2i,j). Because EPGN has not been yet reported as a canonical component of the SASP, we also evaluated insulin-like growth factor-binding protein 3 (Igfbp3), identified in silico as the most highly upregulated factor in *Cdkn1a*^+^ keratinocytes and fibroblasts and is recognized as a SASP factor^47–50^. Consistently, *Igfbp3* mRNA localized to the wound site and was detected in p21^+^ keratinocytes and dermal cells (Supplementary Fig. 2a–c). A comparable expression pattern was observed for another factor revealed by our analysis, *Adam8* (Supplementary Fig. 2d). Overall, our results strengthen the association between the phenotype of p21^+^ cells and senescence in wounds and are further supported by the recent finding that p21 drives a secretory phenotype of stressed cells (p21-activated secretory phenotype; PASP)^51^.

Linked to the predicted reduction in cell adhesion (Fig. 2a) and results of *Epgn* KD and KO (Fig. 2f–j) we found a *Cdkn1a*-related upregulation of genes that are known to be essential for migration, including placenta expressed transcript 1 (*Plet1*) and β-actin (*Actb*), among others (Supplementary Fig. 1n). The prior has been reported to be associated with pro-migratory properties of cells in wounding^52,53^ and indeed we found that protein expression of PLET1 is elevated at the edge of the epidermal tongues (Supplementary Fig. 1o). The latter is commonly associated with migration of cells and accordingly, we observed an increased level of polymerized, filamentous actin (F-actin) at the epidermal tongue (Supplementary Fig. 1p). To reveal whether p21 is associated with migration in vivo, we performed an analysis of the spatial positioning of p21^+^ keratinocytes in the epidermal tongue and found that they preferentially occupy the front of the epidermal tongue that drives migration (Fig. 2k,l). Similarly, we observed that p21^+^ fibroblasts are at the forefront of the pro-migratory vimentin-high dermis (Supplementary Fig. 1q). Of note, we also detect increased activity of STAT pathways in p21^+^ cells (Supplementary Fig. 1r,s), which is in line with this pathway involvement in senescence, inflammation and migration^54,55^. Overall, these data indicate that p21^+^ cells are associated with inflammation and migration of skin cells in wounds.

### Senescence is induced as a rapid-onset response to wounding

**Fig. 3:**
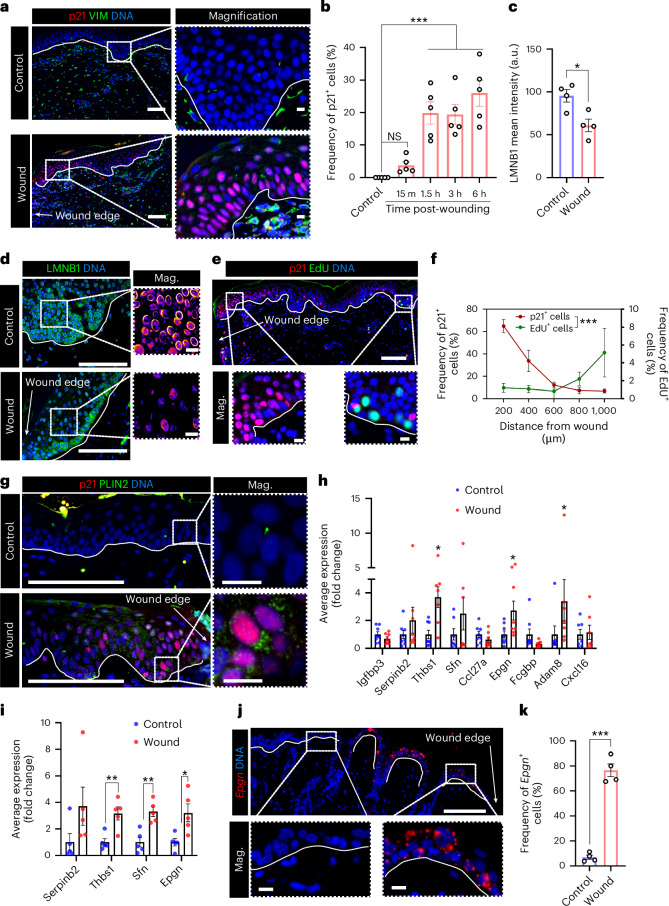
Cellular senescence is induced as a rapid-onset response to wounding. **a**, Representative images showing staining against p21 (red) and vimentin (VIM; green) of control porcine skin or 3 h after induction of an excision wound. Wound is on the right side and magnifications show regions marked with white dashed lines. **b**, Quantification of the frequency of p21^+^ keratinocytes in control porcine skin and following wounding at time points 15 min, 1.5 h, 3 h and 6 h. *P* values (for control versus time points after wounding) are 0.7436 (15 min), 0.0002 (1.5 h), 0.0003 (3 h) and <0.0001 (6 h). **c**, Quantification of the average intensity of Lamin B1 (LMNB1) in stratum spinosum keratinocytes at the wound and control sites. *P* value is 0,0157. **d**, Representative images of LMNB1 in porcine skin 6 h after wounding and at the unwounded site of the skin. The magnifications on the right show heatmaps of LMNB1 signal intensity. **e**, Representative images p21 (red) and EdU (green) staining in porcine skin following excision wounding. **f**, Quantification of an average frequency of p21^+^/EdU^−^ and p21^−^/EdU^+^ keratinocytes in porcine skin at 6 h after wounding and in relation to the distance from the wound. *P* value is <0.0001. **g**, Representative images of p21 (red) and Perilipin 2 (PLIN2; green) in porcine skin at 6 h after injury. **h**, RT–qPCR results of expression of genes encoding secretory protein at 3 h wounding of mouse skin in vivo. *P* values (for control versus wound) are 0.722415 (Igfbp3), 0.220338 (Serpinb2), 0.002293 (Thbs1), 0.093060 (Sfn), 0.651435 (Ccl27a), 0.041824 (Epgn), 0.422701 (Fcgbp), 0.008699 (Adam8) and 0.867469 (Cxcl16). **i**, RT–qPCR results of expression of genes encoding secretory protein at 6 h wounding of mouse skin ex vivo. *P* values (for control versus wound) are 0.124871 (Serpinb2), 0.004461 (Thbs1), 0.001992 (Sfn) and 0.015525 (Epgn). **j**, Representative images of *Epgn* (mRNA)^+^ staining in mouse skin following excision wounding. **k**, Quantification of an average number of *Epgn* (mRNA)^+^ keratinocytes in mouse skin at 6 h after wounding. *P* value is <0.0001. In all the images DNA is stained with DAPI (blue) and dashed white line marks basal lamina of epidermis. Data are from *n* = 5 pigs per group (**b**), *n* = 4 pigs per group (**c**,**f**), *n* = 6–7 mice (**h**), *n* = 5 mice (**i**) and *n* = 4 mice (**k**). Mean ± s.e.m. is plotted. For **b**, a one-way ANOVA with post hoc Dunnett’s test (two-sided) was used and an unpaired t-test (two-sided) was used for **c**,**k**. For **f**, a two-way ANOVA was used. For **h**,**i**, an unpaired *t*-test (two-sided) normalized for multiple comparisons was used. *P < 0.05, **P < 0.01, ***P < 0.001. For all images scale bars show 100 μm, for their micrographs scale bars are 10 μm. Source numerical data are available in source data.

**Extended Data Fig. 4:**
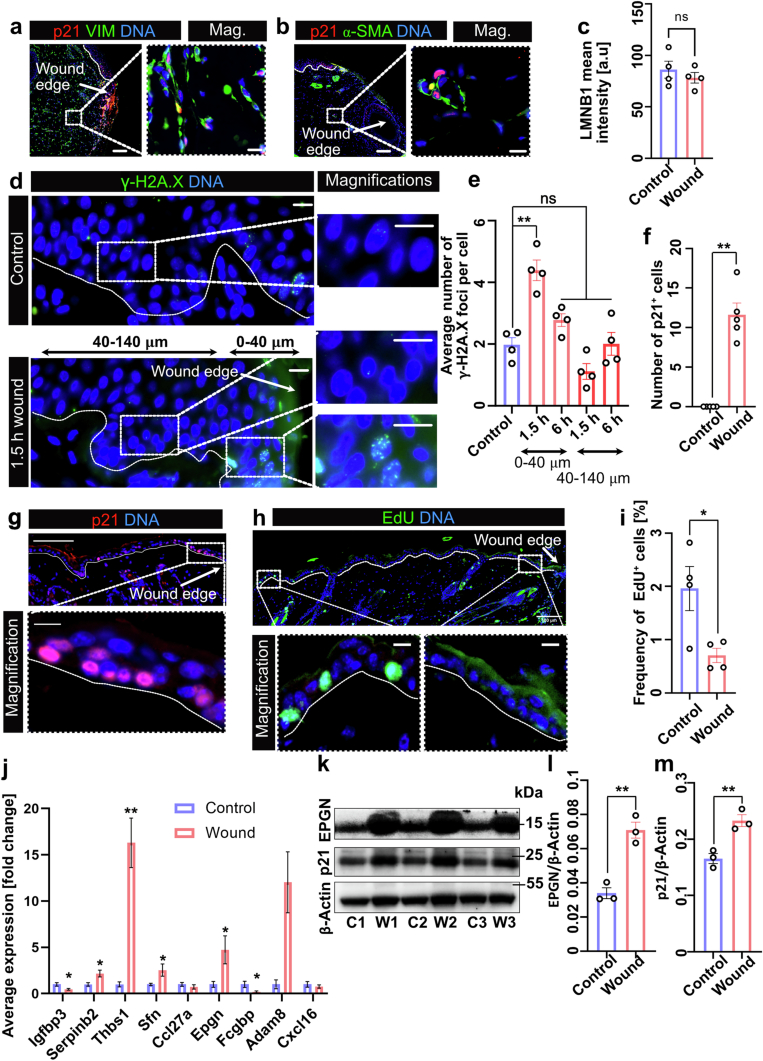
p21^+^ cells are induced as a rapid-onset response to wounding. (**a**) Representative images of immunofluorescent staining against p21 (red) and vimentin (VIM, green) in porcine skin 3 h after wounding. This experiment was performed once. (**b**) Representative images of immunofluorescent staining against p21 (red) and alpha smooth muscle actinin (α-SMA; green) in porcine skin 3 h after wounding. (**c**) Quantification of Lamin B1 (LMNB1) in stratum basale keratinocytes at the wounded and control sites of porcine skin 6 h after wounding. (**d**) Representative images of γ-H2A.X (green) staining in control and wounded porcine skin. (**e**) Quantification of an average number of γ-H2A.X per keratinocyte immediately adjacent (0–40 μm) and not -adjacent (40–140 μm) to the injury site. P values are: <0,0001 (Control vs. 1.5 h 0–40 μm), 0,1897 (Control vs. 6 h 0–40 μm), 0,1540 (Control vs. 1.5 h 40–140 μm) and >0,9999 (Control vs. 6 h 40–140 μm). (**f**) Quantification of an average number of p21^+^ keratinocytes in mouse skin at 6 h after wounding. P value is <0,0001. (**g**) Representative images of p21^+^ cells in mouse skin following excision wounding. (**h**) Representative images of EdU^+^ cells in mouse skin following excision wounding. (**i**) Quantification of an average number of EdU^+^ keratinocytes at the wound site and compared to the distal (control) site at 6 h after wounding. P value is 0,0280. (**j**) RT-qPCR results of expression of genes encoding secretory protein at 6 h wounding of mouse skin in vivo. P values (for Control vs. Wound) are: 0,024990 (*Igfbp3*), 0,014668 (*Serpinb2*), 0,000281 (*Thbs1*), 0,038212 (*Sfn*), 0,408146 (*Ccl27a*), 0,046902 (*Epgn*), 0,032675 (*Fcgbp*), 0,010877 (*Adam8*) and 0,516243 (*Cxcl16*). (**k**) Western blot (WB) results of p21 and EPGN levels in control (**c**) or wound (**w**) samples porcine skin collected 3 h after an injury. (**l**) Quantification of WB results for p21 protein level. P value is 0,0028. (**m**) Quantification of WB results for EPGN protein level. P value is 0,0082. In all the images DNA is stained with DAPI (blue). Data are from n = 4 pigs per group for (**c**) and (**e**), n = 4 mice for (**f**) and (**i**), from n = 6–7 mice for (**j**) and n = 3 pigs for (**l**) and (**m**). Mean ± SEM plotted. For the graph (**c**), (**f**), (**i**), (**l**) and (**m**) an unpaired t-test, for (**e**) one-way ANOVA with post-hoc Dunnett’s test (two-sided) and for (**j**) an unpaired t-test (two-sided) normalized for multiple comparisons was used. * is p < 0.05, ** is p < 0.01 and ‘ns’ stands for ‘non-significant’. Scale bars for (**a**), (**b**), (**g**) and (**h**) show 100 and 10 μm for low and high-magnification images respectively and for (**d**) scale bars show 10 μm. Source numerical data and unprocessed blots are available in source data.

Inspired by our finding that p21^+^ cells were already present in high quantity at the wound site at 3 d post-wounding (Fig. 1b,c and Extended Data Fig. 1b,d,e), and following our recent findings on the immediate responses of skin to wounding by establishing the p-rpS6-zone^13^, we characterized changes in the number of p21^+^ cells within minutes to hours after wounding (Fig. 3). We therefore utilized porcine skin as it closely resembles human skin and allows for the collection of multiple samples and time points from a single animal, using an established excision wound model^13^. We discovered that the first p21^+^ keratinocytes appear within minutes, reaching a statistically significant difference as early as 1.5 h after wounding (Fig. 3a,b). The rapid-onset induction of p21 was obvious not only in keratinocytes (Fig. 3a,b), but also in fibroblasts and smooth muscle cells (Extended Data Fig. 4a,b). Together with the rapid-onset of p21 we also observed the rapid appearance of another senescence marker, namely the reduction of LMNB1 in stratum spinosum (Fig. 3c,d), but not basale keratinocytes (Extended Data Fig. 4c). Of note, we discovered that in parallel with upregulation of p21, wounded tissue becomes polarized: keratinocytes within ~400 μm of the injury were p21^+^EdU^−^, whereas keratinocytes located >1 mm from the injury were more frequently p21^−^EdU^+^ (Fig. 3e,f) indicating that rapid induction of p21 is associated with reduced proliferation.

Consistent with our observation that p21^+^ cells do not display DNA damage several days after injury (Extended Data Fig. 2i,j), the DNA-damaged cells appearing immediately upon injury do not spatially colocalize with p21^+^ keratinocytes, being present exclusively in the 40 µm nearest the wound, and reducing down to insignificant levels after 6 h post-injury (Extended Data Fig. 4d,e). Finally, in contrast to DNA damage, we observed the formation of PLIN2^+^ vesicles at the injury site within 6 h post-injury (Fig. 3g), signalling the rapid appearance of lipid droplets.

The rapid-onset cellular senescence seems to be conserved in mammals, as it was just as prominent in mouse skin in vivo, where cells at the edge of the recent injury show a prominent upregulation of p21 at 3 h and earlier post-wounding (Extended Data Fig. 4f,g). This is accompanied by a decline in the proliferative capacity as shown by a reduction in the number of EdU^+^ keratinocytes (Extended Data Fig. 4h,i). Of note, we observed an upregulation of the SASP factors Thrombospondin 1 (*Thbs1*), *Adam8* and *Epgn* as early as 3 h after wounding (Fig. 3h) and Stratifin (*Sfn*) at 6 h after wounding (Extended Data Fig. 4j). To distinguish whether upregulation of these SASP factors is due to infiltration of immune cells or occurs in the residing (stromal) skin cells, we conducted similar wounding experiments on skin ex vivo (lacking circulation, and thus excluding confounding data from infiltration of immune cells) and observed that numerous SASP factors, including *Thbs1*, *Epgn* and *Sfn*, are significantly upregulated in the injured, but not in the control skin (Fig. 3i). This was further verified spatially in situ, where we observed a prominent upregulation of *Epgn* colocalizing specifically with epidermis near the recent wound (Fig. 3j,k). Finally, to provide further verification of the histological assessment, we performed western blot (WB) of samples collected from porcine wounds and confirmed a rapid increase in p21 and EPGN (Extended Data Fig. 4k–m).

**Extended Data Fig. 5:**
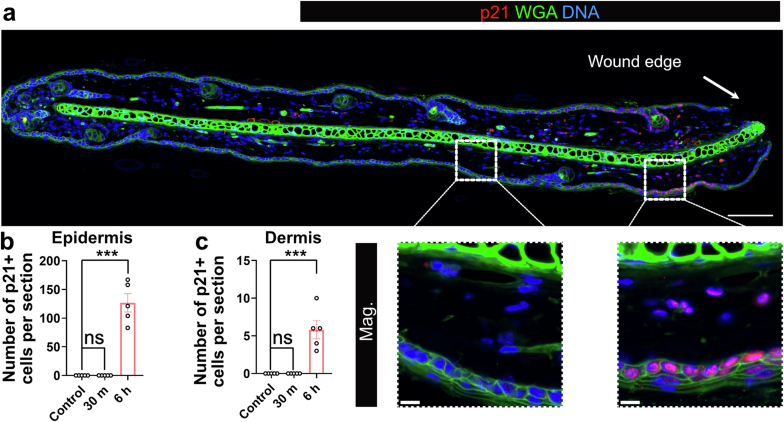
p21^+^ cells are induced as a rapid-onset response to wounding of murine aural tissue. **a**) Representative images of p21 (red) and WGA (Wheat Germ Agglutinin; green) staining in samples of murine aural injury at 6 h post-wounding. DNA is stained with DAPI (blue). The scale bars show 100 μm, for their micrographs scale bars are 10 μm. **b**) Quantification of the number of p21^+^ cells per section in the epidermal compartment next to the injury in the control, unwounded region as well as in injuries at 30 min and 6 h. P values are: >0,9999 (Control vs. 30 min) and <0,0001 (Control vs. 6 h). **c**) Quantification of the number of p21^+^ cells per section in the dermal compartment next to the injury in the control, unwounded region as well as in injuries at 30 min and 6 h. P values are: >0,9999 (Control vs. 30 min) and 0,0001 (Control vs. 6h). Data are from n = 5 mice. Mean ± SEM plotted. For all the graphs one-way ANOVA with post-hoc Dunnett’s test (two-sided) was used. ***p < 0.001 and ‘ns’ is non-significant. Source numerical data are available in source data.

To further verify that the observed phenotype is not specific to truncal skin, we assessed a murine model of aural (ear) injury. Similar to the skin model, the injury involves excision of tissue, but in this case, it creates a gap in the tissue structure and damages not only the epidermis and dermis but also the underlying cartilage. Consistent with our observations in excisional injuries of the truncal skin, we found that ear tissue also exhibits a rapid upregulation of p21 at the edge of the injury, primarily in the epidermis and dermis (Extended Data Fig. 5a–c). Overall, these data demonstrate that senescence is induced rapidly in response to injury within minutes to hours after wounding.

### Rapidly induced senescent cells remain cell cycle arrested throughout healing

**Fig. 4:**
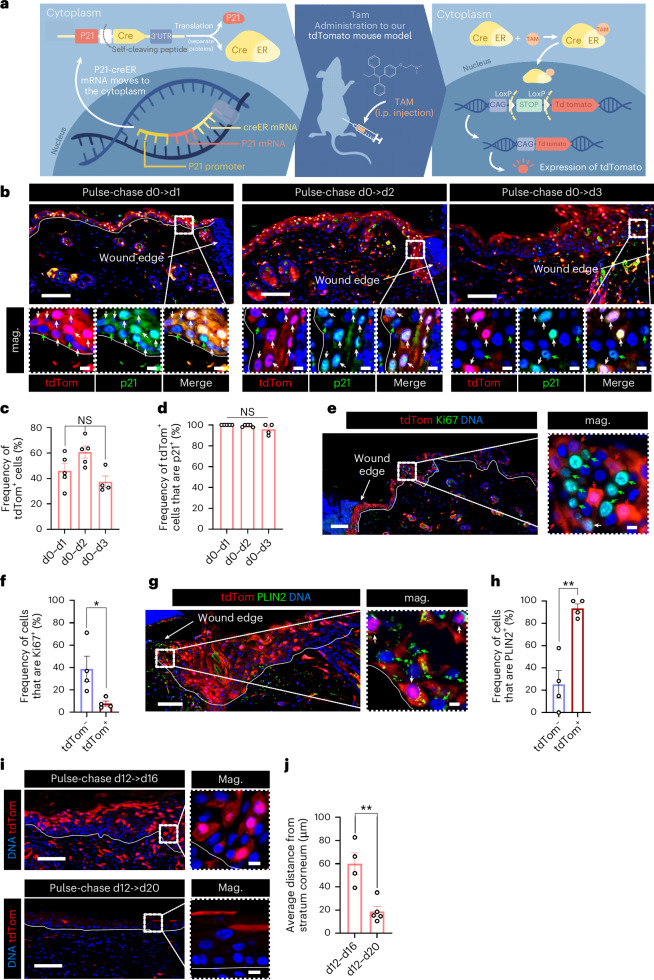
Rapidly induced senescent cells are stable throughout healing and removed upon completion of the healing process. **a**, Schematics showing the mode of function of the p21-creER-R26-tdTomato mouse model. On the left side, condition without Tam is shown, on the right after Tam administration. i.p. intraperitoneal. **b**, Representative images of staining against tdTomato (tdTom, red) and p21 (green) of murine injuries collected 1–3 days after Tam (20 mg kg^−1^) administration performed at the time of injury. White, green and red arrows mark cells positive for both tdTom and p21, only for p21 and only for tdTom, respectively. **c**, Quantification of the frequency of tdTom^+^ keratinocytes that are 1–3 days after induction. **d**, Quantification of the frequency of tdTom^+^ keratinocytes that show expression of p21 at 1–3 days after the Tam pulse. **e**, A representative image of staining against tdTom (red) and Ki67 (green) of murine injuries collected 3 days after Tam administration performed at the time of injury. White, green and red arrows mark cells positive for both tdTom and Ki67, only for Ki67 and only for tdTom, respectively. **f**, Quantification of the frequency of Ki67^+^ cells among tdTom^+^ and tdTom^−^ cells. *P* value is 0.0366. **g**, A representative image of staining against tdTom (red) and PLIN2 (green) of murine injuries collected 3 days after Tam administration performed at the time of injury. White and green arrows mark keratinocytes that are tdTom^+^/PLIN2^+^ and only PLIN2^+^, respectively. **h**, Quantification of the intensity of LMNB1 among tdTom^+^ and tdTom^−^ keratinocytes. *P* value is 0.0019. **i**, Representative images of staining against tdTom (red) of the sites of murine injuries collected 16 and 20 days upon wounding and 4 and 8 days upon Tam treatment, respectively. **j**, Quantification of the distance of tdTom^+^ keratinocytes from the outer layer of epidermis, stratum corneum. *P* value is 0.0035. In all images DNA is visualized by DAPI (blue) and dashed white line marks basal lamina of epidermis. Data are from *n* = 4–5 mice (**c**,**d**,**j**), *n* = 4 mice (**f**,**h**). Mean ± s.e.m. is plotted. For **c**,**d**, **a** one-way ANOVA with post hoc Dunnett’s test (two-sided) was used and for **f**,**h**,**j**, an unpaired *t*-test (two-sided) was used. *P < 0.05, **P < 0.01, NS, not significant. Scale bars, 100 μm and 10 μm (micrographs). Source numerical data are available in source data.

The previous experiments have shown that rapidly induced p21^+^ cells display numerous markers of cellular senescence. Yet, given the unexpected speed of their induction, it could be inferred that the observed phenomenon is transient or reversible. Investigating the persistence of senescent cells is challenging in vivo, as samples collected from animals display only a snapshot of their physiology, without evidence of whether an observed phenotype persists or how long it was present before killing. To address this issue, we crossed p21-creER and Rosa26-tdTomato strains to generate inducible lineage-tracing p21-creER-R26-tdTomato (p21-lineage-tracing) mice^56^. These mice express endogenous p21 with a self-cleaving peptide holding creER (Fig. 4a and Methods). Upon translation of p21, the peptide cleaves, releasing creER, which is transported to the nucleus solely upon the presence of tamoxifen (Tam), leading to the removal of a STOP codon and the subsequent expression of an ultra-bright red fluorescent reporter with tandem-dimer Tomato (tdTom). In this way, a ‘senescent-cell-lineage’ tracing of p21^+^ cells is possible, through administration of Tam at specific time points after injury in a pulse–chase labelling approach.

**Extended Data Fig. 6:**
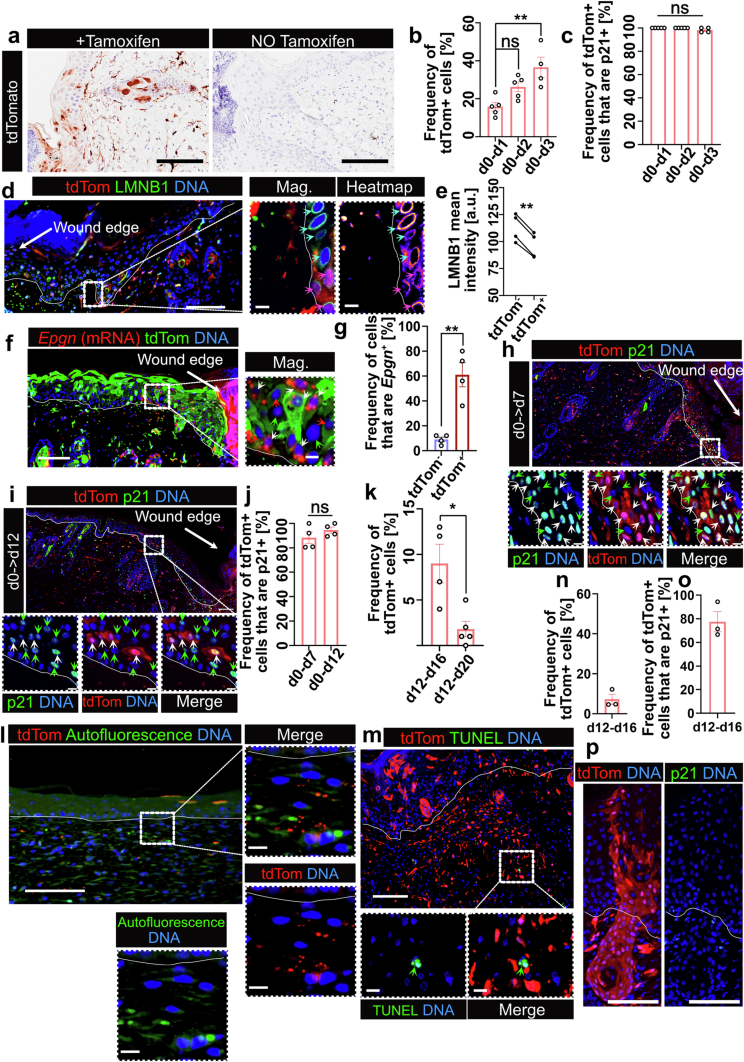
Rapidly induced senescent cells are stable throughout healing and removed upon completion of the healing process. (**a**) Representative images of IHC staining against tdTomato (tdTom) of wound samples collected from p21-tracker mice with or without Tamoxifen (Tam; 20 mg/kg) treatment. (**b**) Quantification of the frequency of tdTom^+^ dermal cells that are 1–3 days after induction. P values are: 0,0776 (d0-d1 vs. d0-d2) and 0,0026 (d0-d1 vs. d0-d3). (**c**) Quantification of the frequency of tdTom^+^ dermal cells that show expression of p21 at 1–3 days after the Tam pulse. (**d**) A representative image of staining against tdTom (red) and LMNB1 (green) of murine injuries collected 3 days after Tam administration performed at the time of injury. Magenta and cyan arrows mark cells positive and negative for tdTom, respectively. (**e**) Quantification of the LMNB1 intensity among tdTom^+^ and tdTom^−^ cells. P value is 0,0007. (**f**) A representative image of staining against *Epgn* (red) and tdTom (green) of murine injuries collected 3 days after Tam administration performed at the time of injury. White, red and green arrows mark cells positive for tdTom and *Epgn*, positive for tdTom, but negative for *Epgn* and negative for both signals, respectively. (**g**) Quantification of the frequency of *Epgn*^+^ cells among tdTom^+^ and tdTom^−^ cells. P value is 0,0019. (**h**) Representative images of a staining against tdTomato (red) and p21 (green) of samples from injury sites collected at 7 days and (**i**) 12 days post-wounding from p21-tracker mice that were treated with Tam at day 0. White, green and red arrows mark cells positive for both tdTom and p21, only for p21 and only for tdTom, respectively. (**j**) Quantification of the frequency of tdTom^+^ keratinocytes that show expression of p21 at 7–12 days after the Tam pulse conduced at the day 0. (**k**) Quantification of the frequency of tdTom^+^ epidermal cells in samples collected at days 16 and 20, at 4 and 8 days after a Tam pulse. P value is 0,0111. (**l**) A representative image of a staining against tdTomato (red) of a sample from an injury site collected at 20 days post-wounding from a p21-tracker mouse that was treated with Tam at day 12. Autofluorescence is visualized in green. (**m**) A representative image of TUNEL (green) and tdTomato (red) staining of an injury sample collected at 16 days post-wounding from a p21-tracker mouse that was treated with Tam at day 12. (**n**) Quantification of the frequency of tdTom^+^ keratinocytes 16 days post-wounding from p21-tracker mice that were treated with Tam at day 12 and (**o**) the corresponding quantification of the frequency of tdTom^+^ keratinocytes that show expression of p21. (**p**) A representative image of a single instance of a cluster of tdTom^+^, but p21^−^ keratinocytes. In all images DNA is visualized by DAPI (blue) and dashed white lines mark basal lamina of epidermis. Data are from n = 4–5 mice for (**b**), (**c**) and (**k**), from n = 4 mice for (**e**), (**g**) and (**j**) and from n = 3 mice for (**n**) and (**o**). Mean ± SEM plotted for (**b**), (**c**), (**g**), (**j**), (**k**), (**n**) and (**o**) and individual values plotted for (**e**). For the graphs (**b**) and (**c**) one-way ANOVA with post-hoc Dunnett’s test (two-sided), for (**e**) a paired t-test (two-sided) was used and for (**g**), (**j**) and (**k**) an unpaired t-test (two-sided) was done. *p < 0.05. **p < 0.01 and ‘ns’ is ‘non-significant’. For all images scale bars show 100 μm, for their magnifications the scale bars are 10 μm. Source numerical data are available in source data.

We first confirmed that tdTom was robustly expressed upon skin injury, dependent on Tam administration (Extended Data Fig. 6a). Next, we conducted a pulse–chase study where a single dose of Tam was administered to mice at the time of injury and mice were killed 1–3 days afterwards. We observed that the frequency of tdTom^+^ keratinocytes does not change significantly throughout healing (Fig. 4b,c), indicating their persistence over time. Of note, the frequency of dermal tdTom^+^ cells increases between days 1 and 3 (Extended Data Fig. 6b) suggesting that cells induced to senescence at the time of injury migrate to the injury site, for example, from the underlying fascia as shown^57^ and in accordance with our previous results (Fig. 2). Both keratinocytes and dermal tdTom^+^ cells display a high expression of p21 protein (Fig. 4b,d and Extended Data Fig. 6c) confirming that once induced, the expression of p21 is stably maintained. Finally, we verified that tdTom^+^ keratinocytes are co-positive not only for p21, but also for other senescence markers: low levels of proliferation-specific protein Ki67 (Fig. 4e,f), high levels of PLIN2-associated lipid droplets (Fig. 4g,h), low levels of LMNB1 (Extended Data Fig. 6d,e) and elevated *Epgn* expression (Extended Data Fig. 6f,g). To validate the findings on the long-term coexpression of p21 and tdTom, we repeated the experiments by injecting p21-creER-R26-tdTomato mice with Tam at day 0, but this time mice were killed at days 7 and 12 (Extended Data Fig. 6h–j). Yet, even with these long-term experiments, the frequency of tdTom^+^ keratinocytes that remained co-positive for p21 stayed at ~90% for 7 (Extended Data Fig. 6h,j) and 12 days (Extended Data Fig. 6i,j). Overall, these results suggest that rapidly induced senescent cells maintain their senescence phenotype for a prolonged period of time.

### Senescent cells are removed upon wound closure through shedding and death

We observed that senescent cells disappear upon the completion of the healing process (Fig. 1), via an unknown mechanism. To dissect the mechanism behind the disappearance of senescent cells, p21-tracker mice received Tam at day 12 post-injury (which is a late stage of healing at which senescent cells are still present, as can be seen in Fig. 1) and killed on days 16 or 20. First, we observed that the frequency of p21^+^ keratinocytes declines when comparing the injury site on days 16 and 20 (Extended Data Fig. 6k). Second, in epidermis we observed an overall decrease in the distance of tdTom^+^ cells from the stratum corneum (Fig. 4i,j) from where keratinocytes shed. Noteworthy, the majority of tdTom^+^ keratinocytes at day 20 are already enucleated (Fig. 4i) indicating that they are in the process of being removed via terminal differentiation and shedding. Likewise, the number of dermal tdTom^+^ cells decreases with a concomitant increase in small vesicles of an intense tdTom signal (Extended Data Fig. 6l). As these vesicles were in close proximity to TUNEL-positive nuclear fragments (Extended Data Fig. 6m) it suggests that p21^+^ dermal cells are eliminated via apoptosis. To validate whether tdTom^+^ cells retain p21 even at the later stages of the healing process, we tracked keratinocytes with a pulse of Tam administered at day 12 until day 16 (Extended Data Fig. 6n,o). While the frequency of tdTom^+^ keratinocytes has markedly decreased (Extended Data Fig. 6n) in comparison to the pulses started at earlier days (suggesting that lower number of senescent cells is generated at the late stages of healing; Fig. 4b), the majority of keratinocytes still showed coexpression with p21 (Extended Data Fig. 6o). In all our tdTom-based tracing experiments of p21-expressing cells in p21-creER-R26-tdTomato mice, we observed only a single instance of a cluster of tdTom^+^ keratinocytes that were largely negative for p21. In this case, the tdTom^+^ keratinocytes originated from the bulb of a hair follicle (Extended Data Fig. 6p), suggesting that some cells may escape senescence, or express insufficient levels of p21 to fully enter it, although this appears to be exceedingly rare. Based on our observations that, upon completion of healing, tdTom^+^ keratinocytes are eliminated through epidermal shedding and tdTom^+^ fibroblasts become co‑positive for apoptotic markers, we conclude that injury‑induced senescent cells are cleared after wound closure via distinct, cell‑type‑specific mechanisms.

### Rapid-onset translation of p21 is made possible by an existing pool of *Cdkn1a* transcript

**Fig. 5:**
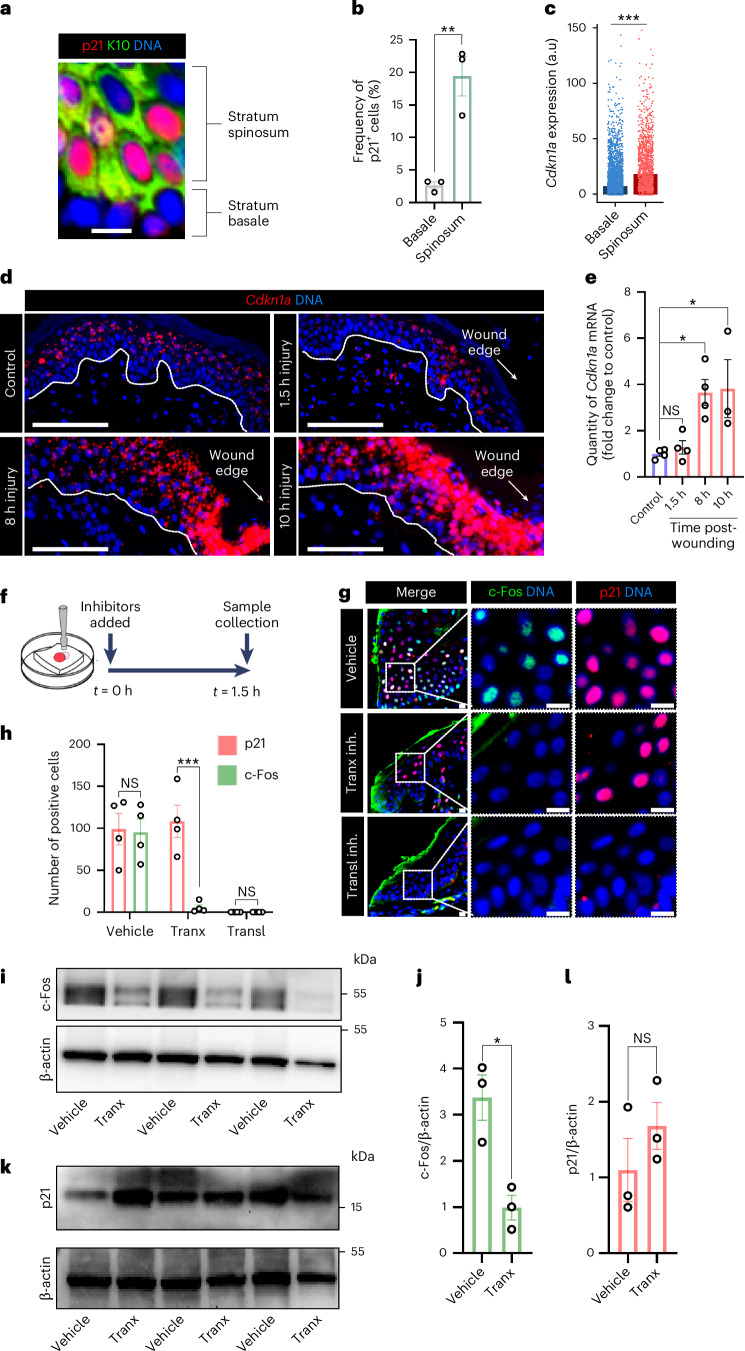
Rapid-onset p21 induction is driven by the existing pool of *Cdkn1a* transcript and is transcription-independent. **a**, Representative images of staining against keratin 10 (K10, green) and p21 (red) of porcine skin collected from a site of an excision wound. **b**, Quantification of the frequency of p21 keratinocytes found in stratum spinosum (K10^+^) and stratum basale (K10^−^). *P* value is 0.0055. **c**, Expression of *Cdkn1a* in keratinocytes from stratum basale and *spinosum* from the sc-RNA-seq dataset. *P* value is <0.0001. **d**, Representative images of RNA-ISH staining against *Cdkn1a* (red) in samples from porcine skin unwounded or wounded and collected at 1.5 h, 8 h and 10 h after injury. **e**, Quantification of the expression of *Cdkn1a* in keratinocytes after 1.5 h, 8 h and 10 h post-injury and normalized to the average of the control skin. *P* values (for control versus time points after wounding) are: 0.9720 (1.5 h), 0.0190 (8 h) and 0.0207 (10 h). **f**, Schematics of the experimental design for ex vivo wounding of porcine skin and administration of drugs. **g**, Representative images of staining against p21 (red) and c-Fos (green) in samples from porcine skin ex vivo wounded and treated for 1.5 h with vehicle (PBS), inhibitors of transcription (Tranx; 50 ng ml^−1^ actinomycin D with 50 µg ml^−1^ α-amanitin in PBS) or inhibitors of translation (Transl inh.; 1 ml cycloheximide in PBS). **h**, Quantification of the number of p21^+^ and c-Fos^+^ keratinocytes 1.5 h after the treatment with the vehicle or the inhibitors. *P* values (for p21 versus c-Fos) are 0.9950 (vehicle), <0.0001 (transcription inhibitors; Tranx) and >0.9999 (translation inhibitors; Transl). **i**, WB of c-Fos and β-actin of porcine samples collected 1.5 h after an injury and a subsequent treatment with vehicle or transcription inhibitors (Tranx). **j**, Quantification of WB results for c-Fos in reference to β-Actin. *P* value is 0,0130. **k**, WB of p21 and β-actin of porcine samples collected 1.5 h after an injury and a subsequent treatment with vehicle or Tranx. **l**, Quantification of WB results for p21 in reference to β-actin. In all the images DNA is stained with DAPI (blue). Data are from *n* = 3 pigs per group (**b**,**j**,**l**), *n* = 3–4 pigs (**e**) and *n* = 4 pigs (**h**). For all graphs, mean ± s.e.m. is plotted. For **b**,**c**,**j**,**l**, an unpaired *t*-test (two-sided) was used. For **e**, a one-way ANOVA with Dunnett’s post hoc test (two-sided) was used. For **h**, a two-way ANOVA with Sidak’s post hoc test (two-sided) was used. *P < 0.05, **P < 0.01, ***P < 0.001, NS, not significant. Scale bars, 10 μm (**a**,**g**) and 100 μm (**d**). Source numerical data and unprocessed blots are available in source data.

To determine the mechanism of the rapid-onset induction of p21 in response to injury, we first asked whether there are certain subtypes of skin cells that would be more responsive to the injury-triggered p21 induction. Differential analysis of p21 induction between keratin 10 (K10)^+^ stratum spinosum keratinocytes and K10^−^ stratum basale keratinocytes revealed that the spinosum keratinocytes become more effectively p21^+^ upon wounding (Fig. 5a,b and overview image in Supplementary Fig. 3a), which is also consistent with these cells losing LMNB1 upon injury (Fig. 3c,d).

Although *Cdkn1a* expression is increased in wounds within several days post-injury (Fig. 1 and Extended Data Fig. 3c,e–g), our sc-RNA-seq atlas indicates that a substantial quantity of *Cdkn1a* mRNA is present even in the unwounded skin (Extended Data Fig. 3b and Supplementary Figs. 1d–f and 3b), despite a distinct lack of p21 protein (Figs. 1 and 3). To establish whether specific subtypes of keratinocytes in unwounded skin show different basal levels of *Cdkn1a* transcript, we interrogated our sc-RNA-seq atlas, which revealed that indeed under homeostatic conditions the spinosum keratinocytes have a higher level of *Cdkn1a* expression than the basale keratinocytes (Fig. 5c). We further verified this finding using RNA ISH against *Cdkn1a* mRNA in mouse skin samples and confirmed that indeed homeostatic keratinocytes contain *Cdkn1a* mRNA, with K14^+^ (basale) keratinocytes showing a lower number of *Cdkn1a* mRNA particles than K10^+^ (spinosum) keratinocytes (Supplementary Fig. 3c,d). This reflects the more robust induction of rapid-onset p21^+^ senescence in spinosum keratinocytes upon injury, when compared with basal keratinocytes (Fig. 5a,b). Likewise, fibroblasts that become p21^+^ in injuries are those of detectable *Cdkn1a* levels (Supplementary Fig. 3e,f). Thus, the basal quantity of *Cdkn1a* transcript in homeostatic skin determines rapid-onset induction of p21 protein expression upon injury.

### Rapid induction of p21 is transcription-independent

Following this line of thought, if the basal level of *Cdkn1a* transcripts enables a rapid-onset induction of p21 protein in response to wounding, this process is likely to be transcription-independent, both due to the necessity of speed and a lack of need, as the mRNA particles are already present in the cells. This hypothesis is also supported by the lack of a significant upregulation of *Cdkn1a* transcript in mice at 3 h and 6 h (Supplementary Fig. 3g,h) or in pigs at 1.5 h (Fig. 5d,e) after wounding in vivo despite the concomitant increase in p21 protein (Fig. 3). However, we observed an increase in *Cdkn1a* transcript levels at 8 h and 10 h post-wounding in porcine injuries (Fig. 5d,e), suggesting that while p21 protein is induced as early as ~90 min after injury, the upregulation of *Cdkn1a* occurs later, presumably contributing to the maintenance of the senescence phenotype.

We sought to critically test the hypothesis of transcription-independent induction of p21 by comparing protein expression of p21 with that of c-Fos, which we^13^ and others^58–60^ have reported to be upregulated in wounds. c-Fos is an immediate early gene that is rapidly expressed in response to a variety of stimuli^58,60,61^ and notably, is transcription dependent^61,62^. To test whether the protein expression dynamics of p21 and c-Fos differ, we used porcine skin ex vivo and added inhibitors of transcription or translation directly to the cavity of the excision wound (Fig. 5d). While vehicle-treated wounds show a high number of p21^+^ and c-Fos^+^ cells, adding translation inhibitors predictably prevented the appearance of both proteins (Fig. 5e,f). Of note, transcription inhibition also abrogated the induction of c-Fos protein in wounds as expected (Fig. 5e,f), yet had no effect on the expression of p21 protein (Fig. 5e,f). These findings were further verified using WB (Fig. 5g–j). Thus, induction of p21 following wounding is a transcription-independent process driven by the existing pool of *Cdkn1a* mRNA transcripts.

### Rapid induction of p21 is independent from DNA damage and is partially controlled by the mTOR signalling

**Extended Data Fig. 7:**
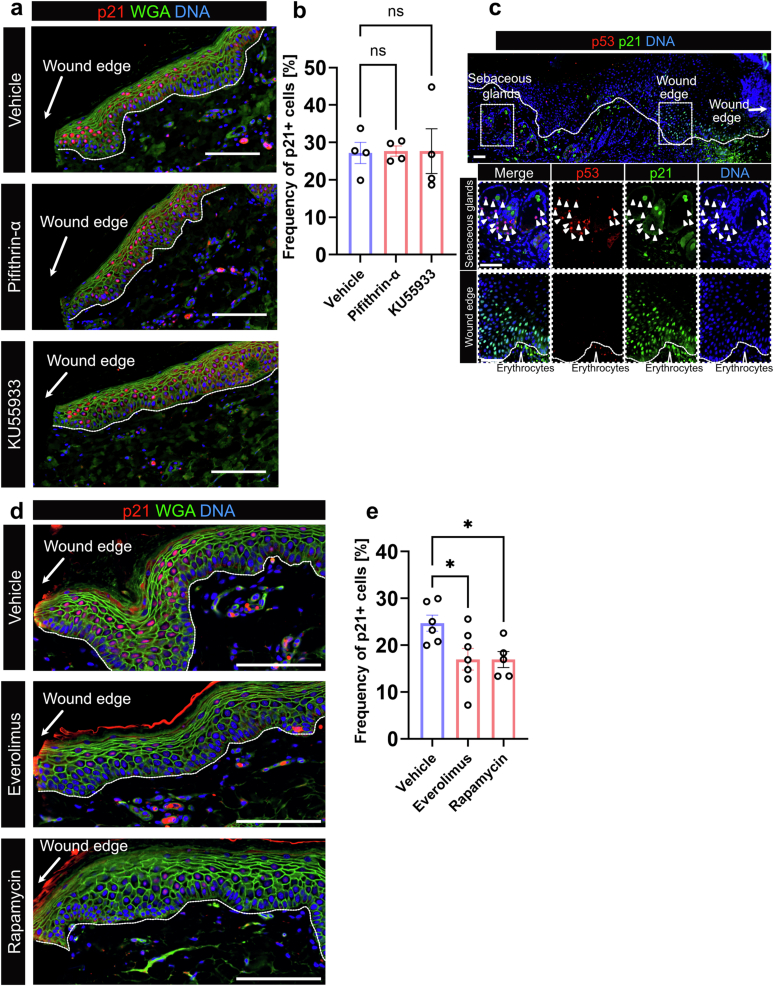
Rapid induction of p21 is independent from DNA damage and partially controlled by the mTOR signalling. **a**) Representative images of staining for p21 (red) in samples from porcine skin ex vivo, wounded and treated for 1.5 h with vehicle (DMSO in PBS), p53 inhibitor (Pifithrin-α; 200 µM), or ATM inhibitor (KU55933; 15 µM). **b**) Quantification of the % of p21^+^ keratinocytes. **c**) Representative images of staining for p21 (green), p53 (red), and DNA (blue) in samples from mouse wounded skin. The upper panel shows a low-magnification view of the peri-wound region. The middle panel shows a magnified view of sebaceous glands. p53^+^ cells are marked with white arrows. The bottom panel shows the epidermis at the edge of the injury. A hollow arrow indicates erythrocytes that are autofluorescent and visible in both green and red channels. **d**) Representative images of staining for p21 (red) in samples from porcine skin ex vivo, wounded and treated for 1.5 h with vehicle (DMSO in PBS) or one of the two mTOR signalling inhibitors, Rapamycin (55 µM) or Everolimus (500 nM). **e**) Quantification of the % of p21^+^ keratinocytes. P values are: 0,0255 (vehicle vs. Everolimus) and 0,0383 (vehicle vs. Rapamycin). In all images DNA (blue) was stained with DAPI, and cell membranes are outlined using WGA (green). The epidermis is outlined in white. Data are from n = 4 pigs for (**b**) and from n = 5–7 pigs for (**e**); Mean ± SEM are plotted. For all graphs one-way ANOVA with post-hoc Dunnett’s test (two-sided) was used for statistical analysis. For images (**a**) and (**d**) scale bars represent 100 μm and for (**c**) scale bars represent 50 μm. Source numerical data are available in source data.

To investigate the upstream mechanism underlying rapid induction of p21, we utilized our recent model of skin injury ex vivo^63^. In brief, in this model a test substance is added directly to the injury cavity for a short period of time following an incubation period enabling the development of cellular responses resulting from wounding^63^. Our initial hypothesis was that DNA damage and the DNA damage response (DDR), both well-established triggers of senescence^2,7^, might be involved. Yet, our experiments revealed that inhibition of p53 or ATM does not prevent the induction of p21^+^ keratinocytes at the injury site (Extended Data Fig. 7a,b). Additionally, we investigated whether p53^+^ cells overlap with p21^+^ cells. Of note, we found that while p21^+^ keratinocytes do not exhibit detectable levels of p53, other cells located farther from the injury site do (Extended Data Fig. 7c). In particular, cells of the sebaceous glands seem to express high levels of p53 in the absence of p21 (Extended Data Fig. 7c).

Inspired by previous publications on the role of mTOR signalling in driving SASP and senescence^21,64–66^, we aimed to investigate the relationship between mTOR signalling and rapid-onset senescence induction. To this end, we used two well-established mTOR inhibitors: everolimus and rapamycin. Notably, we found that both inhibitors significantly, though not completely, reduced the number of p21^+^ cells at the edge of the injury cavity (Extended Data Fig. 7d,e). Overall, these results indicate that the rapid-onset induction of p21 might be dependent on mTOR signalling rather than the DDR.

### Release of nuclear export-blocking proteins from *Cdkn1a* mRNA enables rapid-onset induction of p21 upon injury

**Fig. 6:**
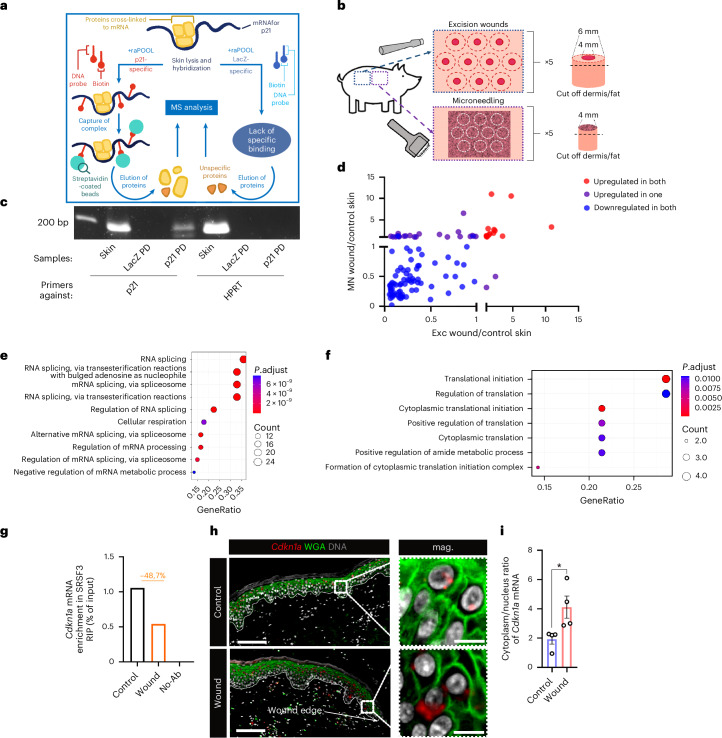
Release of nuclear export-blocking proteins from *Cdkn1a* mRNA enables rapid-onset induction of p21 upon injury. **a**, The experimental design for the pulldown (PD) of *Cdkn1a* mRNA is as follows: Crosslinked *Cdkn1a* mRNA and its protein binding partners are captured using raPOOL probes specific to either *Cdkn1a* mRNA or *LacZ* (as a negative control). Subsequent proteomics analysis reveals the binding partners of *Cdkn1a* mRNA under both homeostatic conditions and in wounds. **b**, The experimental design for sample collection in the PD experiment is as follows: porcine skin is subjected to one of three conditions: excision injury (Exc. wound; 1 mm-thick ring around the injury site), microneedling (MN) or left unwounded (control). To obtain a sufficient number of p21^+^ cells, the layers beneath the epidermis are removed. **c**, Gel electrophoresis of the PD products amplified by RT–qPCR, assessing the quantity of Cdkn1a and HPRT (negative control) cDNA. The experiment was performed on positive control samples (wounded porcine skin before PD), and on samples after *Cdkn1a* and *LacZ* mRNA PDs. The experiment was performed once. **d**, The results of the proteomics analysis show *Cdkn1a* mRNA-protein interactions that are upregulated or downregulated under both types of wounding (represented by red and blue dots, respectively) or only under one type of wounding (represented by purple dots). **e**,**f**, Dot plot showing GO enriched KEGG terms for downregulated (**e**) and upregulated (**f**) DEGs in both types of wounding, based on the proteomics analysis results from the PD experiment. **g**, Results of RNA immunoprecipitation (RIP) followed by qPCR analysis of wounded and unwounded porcine skin showing the change in the amount of *Cdkn1a* mRNA bound to SRSF3 before (‘Control’) and 1.5 h after excision injury (‘Wound’). ‘No-Ab’ indicates RIP–qPCR when no SRSF3 antibody was used. **h**, Representative images of staining for *Cdkn1a* mRNA (red) and co-stained with WGA (green) in porcine skin collected from an excision wound site or a control site. For better contrast, DNA staining is shown in grey. **i**, Quantification of the cytoplasm-to-nucleus ratio of *Cdkn1a* mRNA in keratinocytes from an excision wound site or a control site. *P* value is 0.0370. Data are from *n* = 4 per group (**i**). Mean ± s.e.m. is plotted for **i**. For **i**, an unpaired *t*-test (two-sided) was used. *P < 0.05. The bubble plots (**e**,**f**) were generated using enrichment analysis with Enrichr via gseapy, a Python package. The analysis employed Fisher’s exact test for over-representation, using a one-sided (right-tailed) test to assess enrichment relative to the background. Multiple-testing correction was applied using the Benjamini–Hochberg FDR method across terms within each library. Scale bars, 100 μm and 10 μm (micrographs). Source numerical data are available in source data.

To elucidate the mechanism responsible for the absence of p21 (protein) in homeostatic skin and its rapid generation upon injury, we performed a pulldown experiment of *Cdkn1a* mRNA and its protein binding partners (Fig. 6a). For this, we utilized a variant of the comprehensive identification of RNA-binding proteins by mass spectrometry (ChIRP-MS), which has been successfully used in human cells for large noncoding RNAs^67^, but never before applied to tissues in vivo or to small targets such as *Cdkn1a* mRNA.

**Extended Data Fig. 8:**
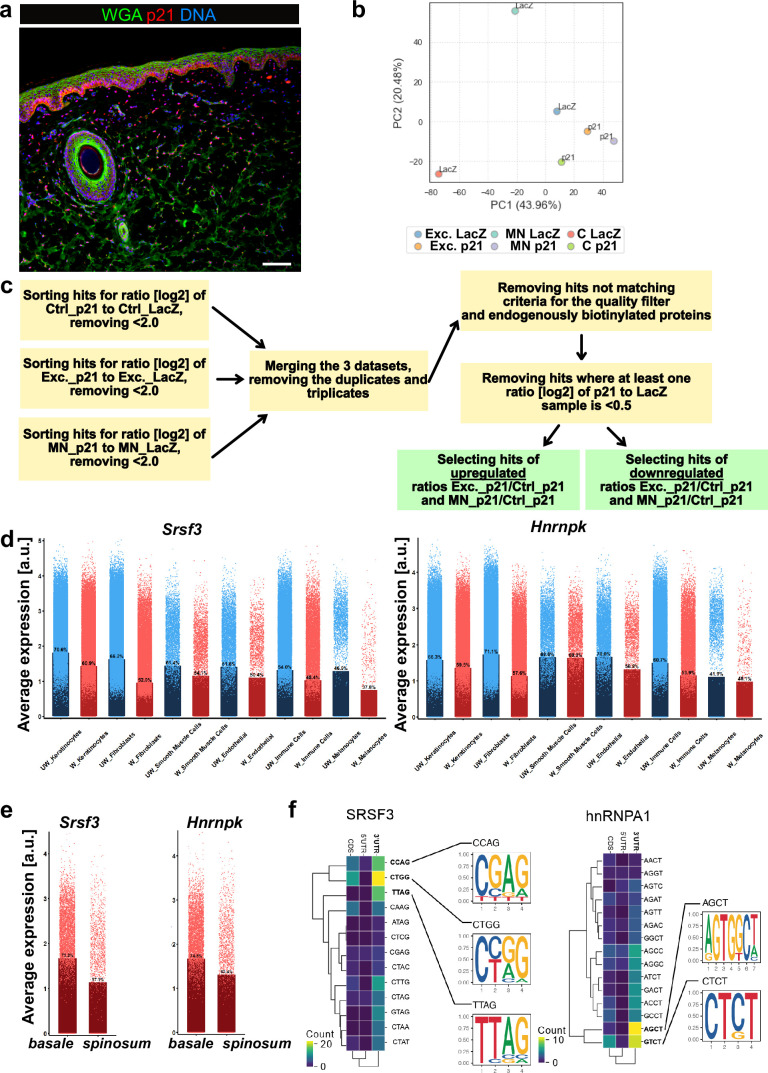
Release of nuclear export-blocking proteins from *Cdkn1a* mRNA enables rapid-onset induction of p21 upon injury. (**a**) Representative images of IHF staining against p21 (red) and co-stained with WGA in porcine skin, which underwent microneedling. DNA is labelled by DAPI and shown in blue. This experiment was performed once. (**b**) Principal component analysis (PCA) of the proteomics results for porcine skin samples which underwent excisional wounding (Exc.) or microneedling (MN) or was untreated (Ctrl; C) and on which pulldown experiment for *Cdkn1a* mRNA (in schematics ‘p21’) or LacZ. (**c**) Pipeline of the proteomics data analysis. (**d**) Average expression levels of *Srsf3* (left side) and *Hnrnpk* (right side) among types of skin cells from samples of control (unwounded; UW) and wounded (**w**) mouse skin used to create the sc-RNA-seq mouse skin atlas. Each dot represents an individual cell and an average is shown as a bar-graph. Values above each bar show the % of population positive the presence of *Srsf3* or *Hnrnpk* transcript. (**e**) Average expression levels of *Srsf3* (left side) and *Hnrnpk* (right side) among keratinocytes from unwounded murine skin identified as originating from *stratum basale* or *stratum spinosum* layers of epidermis. Each dot represents an individual cell and an average is shown as a bar-graph. Values above each bar show the % of population positive the presence of *Srsf3* or *Hnrnpk* transcript. Scale bar shows 100 μm. (**f**) Sub‑motif enrichment patterns for SRSF3 and hnRNPA1 across *Cdkn1a* transcript regions. Clustered heatmaps showing the distribution of individual SRSF3 (left) and hnRNPA1 (right) sub‑motifs across the 5′UTR, coding sequence (CDS), and 3′UTR of *Cdkn1a*. DNA logo plots show evolutionary conservation of *Cdkn1a* binding motif sequences across mammals.

One of the challenges was to obtain a sufficient amount of tissue enriched in cells undergoing rapid-onset senescence (the aforementioned study used 500,000,000 cells). To address this, in addition to collecting a large number of samples (Fig. 6b), we performed an additional type of wounding, microneedling (MN), which we found effective in increasing p21 (protein) expression over a large skin surface area (Extended Data Fig. 8a)

First, we validated the specificity of the mRNA pulldown (Fig. 6c) by comparing RT–qPCR products for *Cdkn1a* and hypoxanthine-guanine phosphoribosyl transferase (*HPRT*), with the latter serving as a negative amplification control (Fig. 6a,c). We found that *Cdkn1a* was present in whole wounded skin and in the product of the *Cdkn1a* mRNA pulldown, but not in the product of the LacZ pulldown, which served as a specificity control (Fig. 6a,c). *HPRT* was present solely in the whole wounded skin and was absent in the products of both pulldowns (Fig. 6c). Overall, this experiment demonstrated that the pulldown products are indeed enriched in *Cdkn1a* mRNA, and thus likely also in its binding partners.

Next, we performed proteomics analysis on *Cdkn1a* mRNA and LacZ pulldown samples isolated from unwounded skin (control) as well as from samples that underwent MN or excision wounding (Fig. 6a). The proteomics results (Supplementary Table 5) showed higher clustering of the *Cdkn1a* mRNA pulldown samples, with a further increase in clustering of samples from the two types of wounding (Extended Data Fig. 8b). Following extensive analysis to remove unspecific hits (those present in LacZ pulldown products; Extended Data Fig. 8c and Methods), we arrived at 111 hits (Fig. 6d).

To increase certainty in identifying proteins enriched or downregulated upon injury, we selected those that are upregulated or downregulated under both MN and excisional wounding (Fig. 6d). The subsequent pathway-enrichment analysis revealed that the downregulated proteins are responsible for mRNA processing (Fig. 6e), while the upregulated proteins are responsible for translation initiation (Fig. 6f), including eukaryotic initiation factor (EIF) family members such as EIF2S2, EIF4G2, EIF3E and EIF3M.

While consistent with the idea of rapid-onset induction of translation, the majority of the upregulated proteins are not specific to *Cdkn1a* mRNA. However, the downregulated proteins, especially those belonging to the serine/arginine-rich splicing factor (SRSF; including SRSF3 and SRSF1) and heterogeneous nuclear ribonucleoprotein (hnRNP; including hnRNPH2, hnRNPC, hnRNPK, and 15 more) families, may be specific to *Cdkn1a* mRNA and regulate its expression rate, as shown^68,69^. Moreover, we found that levels of these factors, for example, *Srsf3* and *hnRNPK* negatively correspond to the level of *Cdkn1a* mRNA. Specifically, all types of skin cells isolated from wounds exhibit lower levels of these transcripts when compared with cells isolated from homeostatic cells (Extended Data Fig. 8d). Likewise, cells that maintain a larger pool of *Cdkn1a* mRNA under homeostatic conditions, such as stratum spinosum keratinocytes show reduced levels of *Srsf3* and *hnRNPK* (Extended Data Fig. 8e). Finally, to further confirm the specificity of the interaction, we utilized the ATtRACT database, which predicts RNA-binding protein targets based on sequence and structural features. The analysis indicated strong predicted binding sites for both SRSF3 and hnRNPA1 within the 3′ untranslated region (UTR) of the *Cdkn1a* transcript, in a manner that is conserved across evolutionarily distant species (Extended Data Fig. 8f).

To verify that changes in the abundance of proteins bound to *Cdkn1a* mRNA are not due to overall alterations in protein levels following injury, we performed proteomic analysis of porcine excision injury samples collected using the same procedure as for the *Cdkn1a* mRNA samples, at two time points: 15 min and 4 h post-injury (Supplementary Fig. 4 and Supplementary Table 6). The proteomic analysis identified nearly 8,000 proteins, of which approximately 5,000 were quantified with high confidence, based on sufficient peptide coverage and protein intensity (Supplementary Table 6). As expected, minimal changes were detected at 15 min post-injury (Supplementary Fig. 4a,b), while at 4 h post-injury we observed significant upregulation of 477 proteins and downregulation of 262 proteins (Supplementary Fig. 4a,c). Gene Ontology (GO) term enrichment analysis revealed that proteins upregulated at both early and late time points were primarily associated with coagulation and immune cell infiltration (Supplementary Fig. 4d,e). In contrast, proteins downregulated at the late time point (no proteins were significantly downregulated at the early time point) were mainly linked to translation (Supplementary Fig. 4f), consistent with previously reported rapid translational responses to injury^13,70^. Of note, among the proteins upregulated on *Cdkn1a* mRNA, none showed significant upregulation at the bulk level, and several were downregulated (Supplementary Fig. 5a), highlighting the specificity of the injury-induced protein binding to *Cdkn1a* mRNA. Similarly, among 71 proteins downregulated on *Cdkn1a* mRNA, 2 were upregulated and 15 were downregulated at the bulk level, yet minimally (Supplementary Fig. 5b). For example, while bulk levels of SRSF3 and hnRNPK decreased by approximately 20%, their quantity on *Cdkn1a* mRNA was reduced by ~80% and ~65%, respectively, indicating that most changes occurring on *Cdkn1a* mRNA are specific to the transcript and not primarily driven by global protein expression changes.

To provide additional evidence for the specificity of the SRSF3–*Cdkn1a* mRNA interaction and its dissociation upon injury, we performed RNA immunoprecipitation (RIP) followed by qPCR. Excision wounding of porcine skin was carried out as for the ChIRP‑MS experiments, yielding wounded tissue estimated to contain approximately 500,000,000 p21^+^ cells, along with a similar amount of unwounded control tissue. We immunoprecipitated SRSF3 crosslinked to RNA from both wounded and unwounded skin (Supplementary Fig. 5c), reverse‑transcribed the recovered RNA to cDNA, and RT–qPCR analysis showed that, at 1.5 h after injury, SRSF3 binds only about half of the *Cdkn1a* mRNA relative to unwounded skin (Fig. 6g).

In particular, SRSF3, but also other *Cdkn1a* mRNA binders such as hnRNPK, have been shown to be involved in preventing the export of mRNAs from the nucleus^71,72^. Consistently, we found that the cytoplasm-to-nucleus ratio of *Cdkn1a* mRNA is highly upregulated shortly after injury in vivo (Fig. 6h,i). Collectively, these results suggest that under homeostatic conditions, *Cdkn1a* mRNA is retained in the nucleus by proteins of the SRSF and hnRNP families. Upon injury, these proteins dissociate from the mRNA, enabling its export to the cytoplasm, subsequent binding of EIF family factors and translation.

### Targeting rapid-, but not late-onset senescence in wounds reduces the rate of healing

**Fig. 7:**
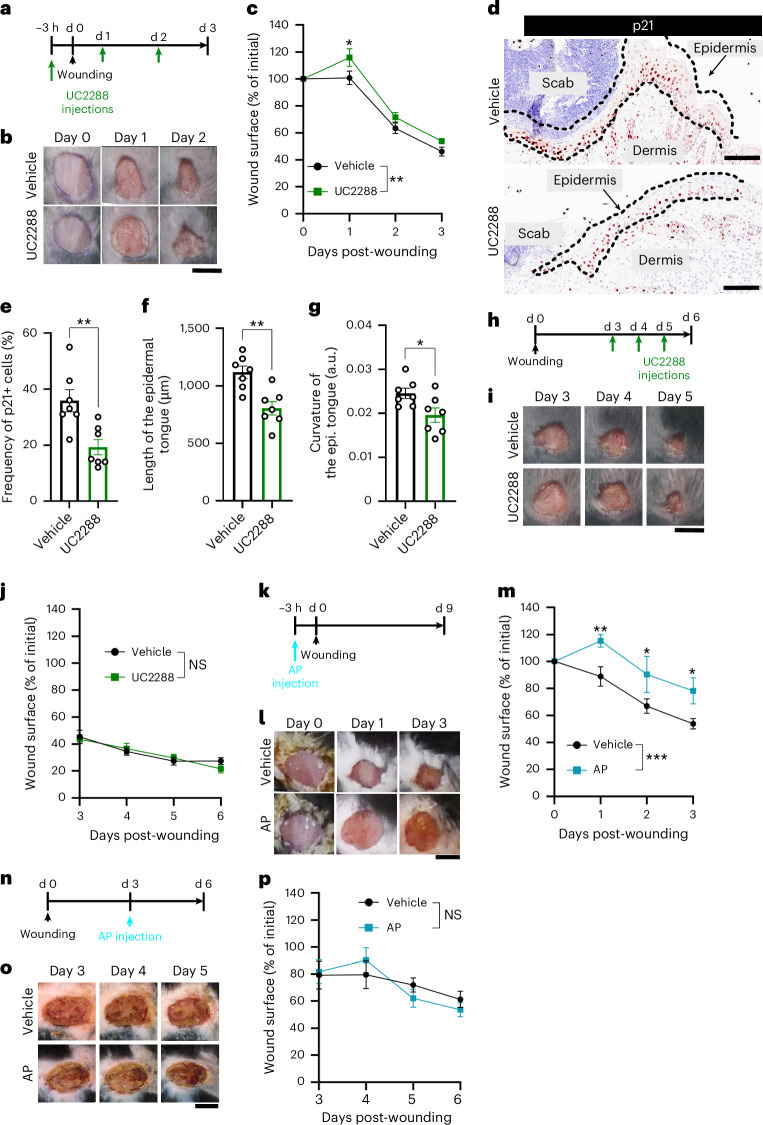
Elimination of rapid-onset senescent cells disrupts healing. **a**, The experimental design for the treatment of mice with a p21 inhibitor UC2288 (10 mg/kg, dissolved at 17.3 mM in DMSO) at days 0–2 post-injury. **b**, Representative photographs of wounds from mice treated with vehicle or UC2288 at days 0–2. **c**, Wound size was normalized to the measurement from day 0 and data are shown as percentage of initial size. *P* value for two-way ANOVA is 0.0042. For the post hoc test, *P* values are (vehicle versus UC2288): 0.0205 (day 1), 0.4100 (day 2) and 0.4568 (day 3). **d**,**e**, Representative images of skin stained for p21 of vehicle- and UC2288-treated mice (**d**) and its corresponding quantification in epidermis (**e**). *P* value is 0.0049. Quantification of parameters associated with re-epithelialization: **f**,**g**, length of the epidermal tongue (*P* value is 0.0021) (**f**) and its curvature (**g**) (*P* value is 0.0279). **h**, The experimental design for the treatment of mice with UC2288 at days 3–5 post-injury. **i**,**j**, Representative photographs of wounds from mice treated with vehicle or UC2288 at days 3–5 (**i**) and its corresponding measurement of wound size (**j**). **k**, The schematics of an experiment with administration of a single dose of AP20187 (AP; 2 mg kg^−1^) or a vehicle solution (Tween80 and PEG400 1:5 in sterile water) to p21-ATTAC mice shortly before wounding and imaging of the healing process throughout 9 days afterwards. **l**, Representative photographs of wounds from mice treated with vehicle or AP. **m**, Wound size was normalized to the measurement from day 0 and data are shown as percentage of initial size for vehicle- and AP-treated homozygotic p21-ATTAC mice. *P* value for two-way ANOVA is 0.0001. For the post hoc test *P* values are (vehicle versus UC2288): 0.0188 (day 1), 0.0237 (day 2) and 0.0367 (day 3). **n**, The schematics of an experiment with administration of a single dose of AP to p21-ATTAC mice at 3 days post-wounding and imaging of the healing process until day 6. **o**, Representative photographs of wounds from mice after a delayed treatment with vehicle or AP. **p**, Graph shows healing rate of mice after a delayed (applied at the day 3) treatment with AP or vehicle. Wound size was normalized to the measurement from day 0 and data are shown as percentage of initial size for vehicle- and AP-treated homozygotic p21-ATTAC mice. Data are, from *n* = 8 per group (**c**,**j**), *n* = 7 per group (**e**–**g**), *n* = 4–7 per group (**m**) and *n* = 6–7 per group (**p**). Mean ± s.e.m. is plotted for all graphs. For **c**,**j**,**m**,**p**, a two-way ANOVA with post hoc Sidak’s test (two-sided) was used and for **e**–**g**, an unpaired *t*-test (two-sided) was used. *P < 0.05, **P < 0.01, ***P < 0.001, NS, not significant. Scale bars, 5 mm (**b**,**i**,**l**) and 100 μm (**d**). Source numerical data are available in source data.

**Extended Data Fig. 9:**
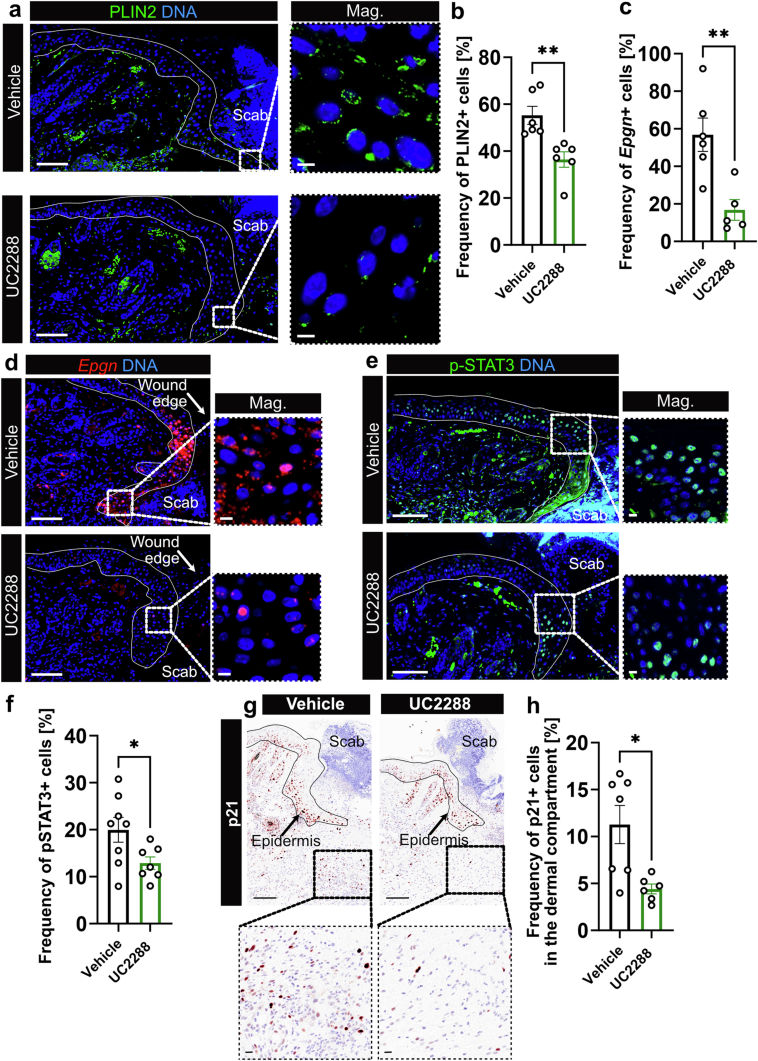
Elimination of rapid-onset senescent cells disrupts healing. (**a**) Representative images of IHF staining against PLIN2 (green) in wounds of vehicle- or UC2288 (p21 inhibitor; 10 mg/kg, dissolved at 17.3 mM in DMSO)-treated mice and (**b**) the corresponding quantification of frequency of PLIN2^+^ epidermal cells. P value is 0,0040. (**c**) The quantification of frequency of *Epgn*^+^ epidermal cells (P value is 0,0054) and (**d**) representative images of IHF staining against *Epgn* mRNA (red) in wounds of vehicle- or UC2288 (p21 inhibitor)-treated mice. (**e**) Representative images of IHF staining against p-STAT3 (green) in wounds of vehicle- or UC2288 (p21 inhibitor)-treated mice and (**f**) the corresponding quantification of frequency of p-STAT3^+^ epidermal cells. P value is 0,0397. (**g**) Representative images of IHC staining against p21 in wounds of vehicle- or UC2288-treated animals and (**h**) its corresponding quantification of the dermal compartment (P value is 0,0114). Data are from n = 6 per group for (**b**) and (**h**) and n = 5–8 for (**c**) and (**f**). Mean ± SEM plotted for all the graphs. For all the graphs an unpaired t-test (two-sided) was used. * is p < 0.05, and ** is p < 0.01. All scale bars show 100 μm and 10 μm for low- and high-magnification images, respectively. Source numerical data are available in source data.

**Extended Data Fig. 10:**
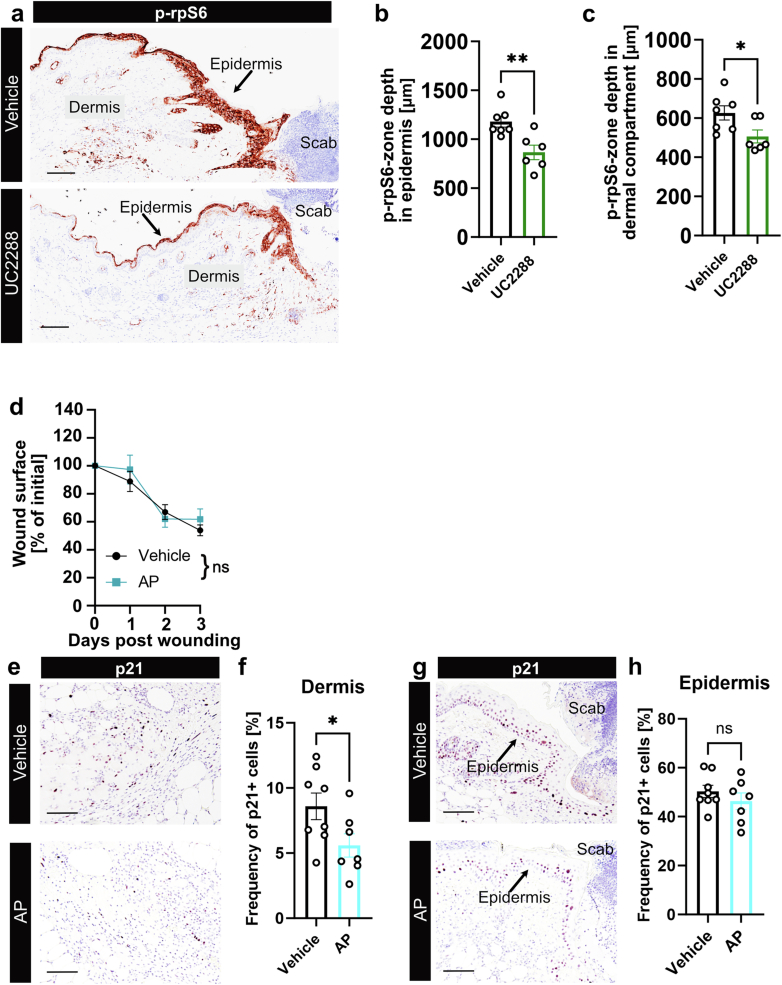
Elimination of rapid-onset senescent cells disrupts healing. (**a**) Representative images of IHC staining against p-rpS6 in wounds of vehicle- or p21 inhibitor (UC2288; 10 mg/kg, dissolved at 17.3 mM in DMSO)-treated animals. Quantifications of the depth of the p-rpS6-zone in **(b)** epidermis (P value is 0,0052) and **(c)** dermis (P value is 0,0339). **(d)** Wound size was normalized to the measurement from day 0 and data is shown as percentage of initial size for vehicle- and AP-treated heterozygotic p21-ATTAC mice. Representative images of IHC against p21 in **(e)** dermis and **(g)** epidermis of wounds collected from vehicle (Tween80, PEG400 1:5 in sterile water)- or AP20187 (AP; 2 mg/kg)-treated p21-ATTAC mice. Quantifications of frequency of p21^+^ cells in **(f)** dermis (P value is 0,0476) and **(h)** epidermis of p21-ATTAC mice. Data are from n = 6–7 for (**b**) and (**c**), n = 4–7 per group for (**d**) and n = 7–8 per group for (**f**) and (**h**). Mean ± SEM plotted for all the graphs. For the graphs (**b**), (**c**), (**f**) and (**h**) an unpaired t-test (two-sided) was used while for the graph (**d**) two-way ANOVA with post-hoc Sidak’s test (two-sided) was used. * is p < 0.05, ** is p < 0.01 and ‘ns’ stands for ‘non-significant’. All scale bars for show 100 μm. Source numerical data are available in source data.

To assess the impact of rapid-onset senescence on physiology, we utilized the p21 inhibitor UC2288 (Fig. 7a), which attenuates *Cdkn1a* mRNA and protein levels^73^. The drug was administered shortly before the excision injury was made, and we found that the treatment reduces the healing rate in the first days after wounding, most prominently on day 1 (Fig. 7b,c). To assess whether the effect is due to senescence reduction of skin cells, we measured the number of p21^+^ cells in epidermis and dermis and morphological changes associated with healing, such as the formation of the epidermal tongue. The inhibitor treatment resulted in fewer p21^+^ epidermal cells (Fig. 7d,e), a concomitant decline in PLIN2^+^ (Extended Data Fig. 9a,b), *Epgn* mRNA^+^ (Extended Data Fig. 9c,d) and p-STAT3^+^ (Extended Data Fig. 9e,f) keratinocytes, as well as reduction in a number of p21^+^ dermal cells (Extended Data Fig. 9g,h). The treatment resulted in an impairment of re-epithelialization visible as a reduction in length (Fig. 7f) and curvature (Fig. 7g) of the epidermal tongue. Consistent with our previous study^13^, inhibition of p21 caused a reduction in the depth of the p-rpS6-zone in epidermis and dermis, fortifying the observations of impaired healing (Extended Data Fig. 10a–c). To assess whether it is specifically rapid-, but not late-onset senescence that is beneficial for healing we administered the p21 inhibitor at a later time point (starting 3 days after injury), maintaining all other experimental conditions (Fig. 7h). In contrast to the results of targeting rapid-onset senescence, there was no significant effect of the late treatment on the rate of healing (Fig. 7i,j), demonstrating that senescence at days after injury has little or no impact on healing.

To further verify the impact of rapid-onset senescence on the physiology of healing, we utilized our recently generated p21-ATTAC mice^74^, in which p21^+^ cells are selectively eliminated in mice using AP20187 (AP). We found that just a single dose of AP, administered shortly before wounding (Fig. 7k), is sufficient to significantly disrupt the healing process (Fig. 7l,m). Of note, while this effect was observed for homozygotic p21-ATTAC mice, we did not observe a significant difference for heterozygotic p21-ATTAC mice (Extended Data Fig. 10d) indicating that heterozygotes contain an insufficient amount of the transgene needed to effectively eliminate p21^+^ senescent cells in skin wounds. We also verified that administration of AP reduces the quantity of p21^+^ dermal cells (Extended Data Fig. 10e,f), albeit without a significant effect on the quantity of p21^+^ keratinocytes (Extended Data Fig. 10g,h) at 24 h from administration. Finally, to confirm that late-onset senescence does not significantly impact healing, we administered AP at 3 days post-injury (Fig. 7n), maintaining all other experimental conditions. Consistent with results from the p21 inhibitor studies, we observed no significant impact of late-AP administration on the rate of healing (Fig. 7o,p). Overall, these results strongly suggest that rapid-onset but not late-onset senescence benefits the physiological process of wound healing.

**Fig. 8:**
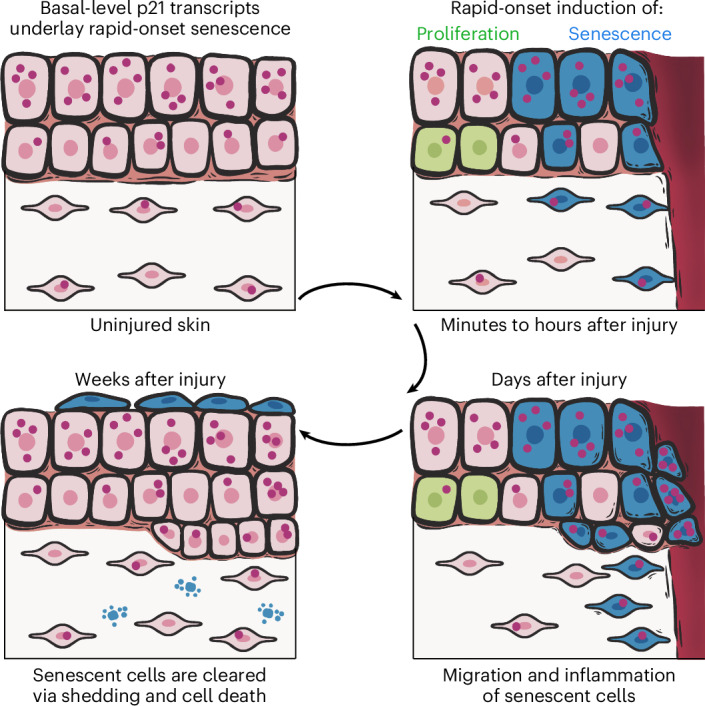
Summary of the research findings. Skin cells in vivo exhibit prominent levels of *Cdkn1a* (p21) mRNA (red dots), with the quantity varying depending on the cell type. Following an injury, *Cdkn1a* mRNA is rapidly utilized to induce senescence at the injury’s edge, which occurs concurrently with the induction of proliferation in the skin regions distal to the injury site. Throughout the healing process, senescent cells contribute to healing by secreting pro-inflammatory and pro-growth factors, and they also facilitate the migration necessary for effective wound closure. Once the healing process is complete, senescent cells are eliminated either through shedding (in the case of keratinocytes) or through apoptosis (in the case of dermal cells).

In summary (Fig. 8), our findings suggest that the skin is ‘prepared’ for potential injury by maintaining pre-existing transcripts of *Cdkn1a* mRNA. Upon injury, p21 is induced independently of transcription. Rapidly induced senescent cells participate in migration and produce various factors that facilitate healing. Once the wound is closed, senescent cells are eliminated through shedding and cell death, leaving the healed skin free of senescence.

## Discussion

As the research field of cellular senescence has been expanded from in vitro to in vivo, it has become obvious that the senescent phenotype is heterogeneous across cell types and conditions. In particular, differences have been observed between p21^+^ and p16^+^ cells, with both subtypes displaying core features of the senescent phenotype, as well as showing differences in features such as SASP, the conditions that cause their induction, and their impact on physiology^51,74,75^. Consistently, here we found that it is senescence-associated with p21 rather than p16 that occurs in skin stromal cells in the context of mammalian injuries. These results were verified using a dedicated p16-reporter mouse line (p16-tdTomato), in silico and biochemical methods and are further supported by recently published results on the impact of p21-dependent senescence on mouse physiology^74,76–78^. Despite the lack of p16, the phenotype described here shows a reliable signature of cellular senescence, including SASP, persistent cell-cycle arrest, ALISE phenotype and others. Similar to the previous study^74,79^ we report a substantial difference between ageing-induced (p16-associated) and injury-induced (p21-associated) cellular senescence. Another aspect of heterogeneity highlighted in this study is that different skin cell types can express similar senescence markers, yet display distinct functional and molecular profiles. Specifically, we observed that senescent smooth muscle cells exhibited higher levels of pro-inflammatory SASP factors (for example, interleukin-6 and CXCL-1), whereas senescent keratinocytes showed a SASP enriched in growth factors. Nonetheless, both cell types also expressed factors characteristic of the other group; for instance, *Cdkn1a*^+^ keratinocytes had elevated CXCL16 and CCL27a. Overall, this heterogeneity is consistent with previous studies^80,81^ and underscores that although cellular senescence involves a secretory and cell cycle arrest phenotype^2^, its consequences vary substantially depending on cell type. One of the shared functions of senescent skin cells, specifically, keratinocytes and fibroblasts, seems to be the spearheading of migration aimed at wound closure. While previous studies have demonstrated that cell cycle arrest is associated with the migration of epidermal cells^82,83^, our findings show that both epidermal and dermal cells exhibit a phenotype that meets the criteria for cellular senescence. Furthermore, our work broadens the understanding of the induction, phenotype, significance and molecular characterization of these senescent cells.

One major finding of our study is the identification of rapid-onset senescence. Cells induced to senesce in a rapid manner do not revert to a proliferative state, as it has been reported for developmental senescence^56^, but instead are removed after the completion of the healing process via mechanisms including shedding.

The concept of ‘rapid-onset senescence’ inherently implies that not all canonical features of senescent cells, such as epigenomic remodelling, lysosomal alterations and temporal dynamics of the secretome, will be present in cells that begin to exhibit early signs of senescence. While we observed a rapid upregulation of numerous senescence markers following injury, it is likely that some features require more time to become established. As certain characteristics, including epigenetic modifications, are technically challenging or even currently infeasible to assess spatially at the single-cell level, we remain hopeful that future studies will elucidate the distinct temporal trajectory of molecular changes associated with rapid senescence induction.

While the concept of rapid senescence induction is reasonable in the context of an effective response to tissue damage, the prevailing view is that senescence takes days or weeks to develop. In relation to animal physiology, we found only a single report where an upregulation of certain senescence markers in liver has been shown to occur within hours after haemorrhagic shock injury^84^. In this respect, a number of rapid-onset mechanisms in the skin and other tissues in response to injury have been identified, including calcium waves, generation of reactive oxygen species, and the formation of the p-rpS6-zone^13,62^. However, judging by how complex the phenotype of cellular senescence is, our finding of a rapid, yet coordinated response on the level of the transcriptome, proteome and metabolome is unique and can provide an example of how cell fate can be modulated in an accelerated manner to enable survival.

In this study, we have investigated primarily the mechanism behind transcription-independent, rapid-onset induction of p21. While our data indicate that reduced binding of SRSFs and hnRNPs to *Cdkn1a* mRNA promotes its nuclear export for cytoplasmic translation, nuclear translation^85^ represents a compelling complementary mechanism that may enable rapid p21 induction under stress. This process allows select mRNAs to engage nuclear ribosomes directly, bypassing export delays through nuclear pores and supporting swift protein synthesis. Future studies employing isolated nuclear pulldowns will be crucial to delineate the relative contributions of these mechanisms and to confirm the binding specificity of the *Cdkn1a* mRNA-binding proteins identified in this study.

While here we focused on p21, it is likely that many other components of the senescence machinery are controlled by transcription-independent, yet distinct mechanisms. Degradation of LMNB1 was suggested to be mediated by proteolytic degradation^86^, thus is likely independent of transcription. Accumulation of lipid droplets might be largely mitochondria-driven^8^ with PLIN2 translocating from cytoplasm to newly generated lipid droplets^87^ instead of a de novo expression. At the same time, however, there could be certain senescence phenotypes depending on a rapid induction of mRNA expression, for example, SASP components. Consistently, we found several SASP components upregulated on the transcript level in a short time span, yet of delayed onset when compared with p21 induction. Regarding the dependence of p21 protein expression on pre-existing *Cdkn1a* transcript, a similar mechanism was shown over two decades ago for interleukin-18, which can be expressed rapidly upon injury in a transcription-independent manner^88^. Yet, when it comes to cellular senescence, we are not aware of reports that its phenotype could be orchestrated in a pre-defined manner based on an existing content of cellular molecular components. It is likely that this mechanism of action is not specific to skin, and that these findings could be replicated in other organs, which may have pre-defined response mechanisms initiated by specific stimuli, with components stored in advance. Thus, in contrast to senescent skin cells that support healing, senescent cells of bone and muscle have been found to impair fracture healing^79,89^ and muscle repair^90^. Moreover, it was recently reported that, under certain treatment regimens in the p21-DTA model, elimination of p21-highly positive cells can actually be beneficial for healing^91^. A key distinction between that study and our findings is that the authors report their construct selectively eliminates ‘p21-highly expressing’ cells. In our study, p21-ATTAC mice undergo targeted apoptosis initiated by FKBP dimerization and subsequent caspase-8 activation, whereas in p21-DTA mice, cell death is induced through inhibition of protein synthesis. Although that study reports reduced tdTom and p21 protein synthesis, the efficiency of selective elimination of p21-positive cells remains unclear. Moreover, as those mice were treated daily with tamoxifen, unlike most of our experiments, which employ a single administration (with the exception of UC2288), this dosing regimen may have been sufficiently intense to elicit side effects, as previously reported^92,93^, potentially influencing wound healing. This finding highlights the need for further comparisons between models and treatment protocols, and we remain optimistic that future follow-up studies using these mice will provide more detailed molecular characterization, facilitating a more comprehensive comparison. Overall, it is crucial to understand the temporal aspect of the impact of senescent cells on regeneration.

We are hopeful that our study will also provide a foundation for future investigations into the mechanisms of damage propagation and the contextual selectivity of rapid-onset senescence. In this respect, our earlier research^13^ showed that damage-associated molecular patterns are at the heart of the rapid responses following the injury, and we expect those to be involved in the induction of the rapid-onset senescence as well. It also remains to be determined whether the phenomenon of rapid-onset senescence is restricted to dorsal skin and ears or whether it can also occur within internal organs, such as the liver or gut, in response to injury. In this regard, examining injuries to the liver, brain and other organs in which the induction of cellular senescence has previously been reported (liver^94,95^ and brain^96,97^) represents a particularly promising direction for future research.

Not only in relation to various tissue types, but the induction of rapid-onset senescence upon mechanical injury is likely to also differ mechanistically from senescence triggered by damage that does not directly breach the tissue integrity, such as UV or γ-irradiation, chemotherapeutics or chemical agents such as 7,12-dimethylbenz[a]anthracene (DMBA)/tetradecanoylphorbol acetate (TPA), all of which primarily act through DNA damage or oxidative stress pathways^98^. Likewise, it is unknown whether conditions that are known to modulate basal level of senescence-related machinery, including levels of *Ckdn1a* mRNA, such as ageing or sun exposure (contrasted by light-protected skin) could impact on the rate or efficiency of the rapid-onset senescence induction in situation of tissue breach such as a mechanical injury. Future work comparing mechanically induced versus irradiation- or drug-evoked senescence, including in light-deprived skin, will help to clarify how environmental exposure influences the skin’s senescence landscape

There have been a number of attempts to explain why senescent cells could be both beneficial and detrimental in a variety of contexts of animal physiology, such as healing, fibrosis and cancer^12,99,100^ and why anti-senescence interventions have variable effects^101^. The hypotheses include moderate versus excessive elimination of senescent cells^101^ and the timing of when senescent cells could be considered beneficial^100^. Our results support that there is a specific time window when senescent cells provide a benefit. These findings are pivotal for ongoing clinical studies with anti-senescence interventions^102^, where it might be possible to narrow down the treatment window to avoid elimination of rapid-onset senescent cells.

## Methods

This research complies with all relevant ethical regulations with study protocols approved by the Municipal Government of Vienna, in accordance with Austrian law and the National Institutes of Health (NIH) Guide for the Care and Use of Laboratory Animals (revised 2011) as well as the Austrian Bundesministerium fur Bildung, Wissenschaft und Forschung and the Institutional Animal Care and Use Committee at Mayo Clinic.

Throughout the execution and completion of manuscript we have been following the guidelines on the minimal information on cellular senescence experimentation in vivo (MICSE)^2^. Light microscopy is further described in Supplementary Table 7.

### Experiments in mice

Mouse experiments were conducted in adherence to ethical guidelines established by the host institution, with the support of the Ludwig Boltzmann Institute (LBI) Trauma veterinarian team. Approval for experiments involving BALB/c mice was obtained in advance from the Municipal Government of Vienna, in accordance with Austrian law and the NIH Guide for the Care and Use of Laboratory Animals (revised 2011). Experimental protocols involving C57BL/6 WT mice and transgenic models: p16-tdTomato, p21-creER-R26-tdTomato and Epgn-KO B6.129-Epgn^tm1^ were approved by the Austrian Bundesministerium fur Bildung, Wissenschaft und Forschung, approval nos. GZ-2022-0.892.233, GZ-2022-0.880.212 and 2024-0.782.004. Approval for the experimental procedures with p21-ATTAC mice was obtained from the Institutional Animal Care and Use Committee at Mayo Clinic under protocol no. A00006837-22.

For all experiments, a maximum of five animals were housed per Type-III cage, maintaining a 12-h light–dark diurnal cycle with room temperature ranging between 21 and 23 °C. Standard rodent diet (Altromin, 1320, LASQC Rod16) and water were provided continuously.

BALB/cJRj and C57BL/6JRj mice were procured from Janvier Labs (cat. nos. SC-BALBJ-F and SC-C57J-F, respectively). Additional genetically modified strains included p16-tdTomato mice provided by N. Sharpless, p21-ATTAC mice from D. Jurk, and both p21-creER and R26-tdTomato mice supplied by B. Zhou. Furthermore, the Epgn-KO B6.129-Epgn^tm1^ strain was kindly provided by M. Dahlhoff.

For all murine experiments, the following wounding procedure was used. Mice were anaesthetized using an oxygen/isoflurane mixture (4–5%) (isoflurane, AbbVie) and anaesthesia was maintained under an oxygen/isoflurane mixture (2–3%), the dorsal hair was shaved and the skin was disinfected with Isozid. A template was employed to outline a 1-cm diameter circle on the posterior dorsal region, and the skin was carefully cut along this circle using scissors to prevent damage to the underlying structures, resulting in a single, full-thickness excision wound. Following the procedure, mice were placed in a cage on a 37 °C heating pad or under a heating lamp until they recovered. Subsequently, they were returned to their cages and housed throughout the study period. Daily monitoring was conducted for signs of infection, pain and weight loss. To document the healing process, digital photographs were taken with a LifeViz Micro camera (Quantificare). The wound area was assessed through planimetric measurements using Fiji image processing software (ImageJ, NIH). At the end of the observation period, mice were killed under deep isoflurane anaesthesia via cervical dislocation. Each wound, along with the surrounding tissue, was collected and placed in a histology cassette, followed by fixation in 10% formalin or freezing.

BALB/c female mice of 12 weeks of age with an average weight of 20–25 g were procured from Janvier Labs. Analgesia (1 mg kg^−1^ of meloxicam orally) was administered 120 min before surgery and continued daily until the end of the study or wound closure.

Housing and wound procedures followed the previously described protocols, with a slight adjustment to the analgesia approach for WT C57BL/6, p16-tdTomato and p21-creER-R26-tdTomato strains. Analgesia (6 mg kg^−1^ carprofen orally) was administered at least 1 h before surgery and continued postoperatively through their drinking water for a duration of 3 days.

For the experiments with p16-tdTomato and WT C57Bl/6 animals either male or female mice 8–20 weeks of age were used. Mice were wounded as described above and killed 3 h, 6 h, 3 days or 7 days after wounding.

In the p21-CreER-R26-tdTomato mouse model, the CreER cassette is knocked into the endogenous p21 (CDKN1A) gene locus as reported^56^. In this model CreERT2 cDNA (encoding the tamoxifen-inducible Cre recombinase) is inserted at the transcriptional stop codon of the endogenous p21 gene (*Cdkn1a*) using CRISPR/Cas9-mediated genome editing. A 2A self-cleaving peptide sequence is included before the CreERT2 cDNA. This allows for the simultaneous expression of both the endogenous p21 protein and the CreERT2 recombinase from a single transcript, due to ribosomal skipping at the 2A site. As a result, in these mice, CreER expression is directly controlled by the endogenous *Cdkn1a* promoter, ensuring that Cre activity reflects the physiological expression pattern of p21 (ref. ^56^).

For the Epgn-KO B6.129-Epgn^tm1^ murine experiment, consisting of 20 female mice (10 WT and 10 Epgn-KO^103^), the following wounding procedure was employed. Analgesics (5.0 mg kg^−1^ body weight Metacam, equivalent to 7 µl of a 15 mg ml^−1^ meloxicam solution) were administered orally 30 minutes before surgery. Mice were subsequently anaesthetized using an oxygen/isoflurane mixture (4–5%; isoflurane, AbbVie), with anaesthesia maintained at 2–3% isoflurane throughout the procedure. The dorsal hair was shaved and the skin was disinfected using Isozid. A template was utilized to outline a 1-cm diameter circle on the posterior dorsal region, and scissors were carefully used to cut along this marking, creating a single, full-thickness excision wound without damaging underlying structures.

After the procedure, mice were placed in a cage on a heating pad at 37 °C or under a heating lamp until full recovery. They were then returned to their housing cages for the remainder of the study. Animals were monitored daily for signs of infection, pain and weight loss. Drinking water with paracetamol (Mexalen, 3.5 mg ml^−1^ drinking water) was offered for the next 2 days. Digital photographs documenting the wound healing process were captured daily using a LifeViz Micro camera (Quantificare). Wound areas were quantified through planimetric analysis using QuPath software v.0.5.1. At the conclusion (14 days after surgery) of the observation period, mice were killed using CO_2_ gas. Each wound, along with adjacent tissues, was collected, placed into histology cassettes and subsequently fixed in 10% formalin or frozen for further analysis.

To investigate early wound healing responses in ears, WT C57BL/6 mice of mixed sex were used. Preoperative analgesia was administered by subcutaneous injection of carprofen (6 mg kg^−1^) at least 1 h before surgery and continued postoperatively via medicated drinking water. Anaesthesia was induced with an oxygen/isoflurane mixture (4–5%) and maintained using 2–3% isoflurane in oxygen (isoflurane, AbbVie). While under anaesthesia, excisional wounds were created by performing a 2-mm biopsy punch on the ear skin. The tissue biopsy was removed, generating a full-thickness wound with a 2-mm diameter. Following the procedure, mice were placed in a recovery cage on a 37 °C heating pad until fully awake. Mice were killed under deep isoflurane anaesthesia by cervical dislocation. Ear punch tissue samples were collected at 30 min and 6 h post-injury. Additionally, control samples were collected from uninjured ear tissue to serve as baseline references. Each ear punch sample was collected into a histology cassette and fixed in 10% formalin at room temperature for 24 h. Tissues were then washed thoroughly with water for 1 h, incubated in 50% ethanol for 1 h and subsequently transferred to 70% ethanol for storage. Samples were stored in 70% ethanol until processing and embedding in paraffin for histological analysis.

In the experiments with p21-creER-R26-tdTomato, either male or female mice of 8–20 weeks of age were used. Tamoxifen pulses (20 mg kg^−1^; Sigma, T5648) were administered on days 0 or 12 after wounding and mice were killed at 1, 2, 3, 7, 12, 16 or 20 days post-wounding.

In the p21-ATTAC mouse experiments male mice of 12–17 weeks of age were used and underwent the same wounding protocol, mice were killed 6 or 9 days after the wound was inflicted. The mice received intraperitoneal (i.p.) injections of AP (AP20187; ARIAD Pharmaceuticals) at a dosage of 2 mg kg^−1^ or a vehicle solution (Tween80 and PEG400 1:5 in sterile water) given 3 h before or 3 days after wounding.

To determine the effect of p21 reduction on healing, we used its inhibitor UC2288 (MedChemExpress, MCE-HY-112780-250mg). For these experiments, p16-tdTomato female mice of 13–15 weeks of age were treated 3 h before or 3 days after wounding, with a 30-µl i.p. injection of UC2288 (10 mg kg^−1^, dissolved at 17.3 mM in dimethylsulfoxide (DMSO)), whereas the control group received a DMSO vehicle injection. Additionally, carprofen (6 mg kg^−1^ diluted in ELO-MEL isotonic solution) was subcutaneously injected 30 min before the operation began and the dorsal excision injury was performed as described above. Treatment was repeated 24 and 48 h after the first injection and mice were killed on day 3 or day 6. Mouse skin was collected and tissue samples were fixed in formaldehyde for 24 h.

For the experiments on murine skin ex vivo, 10–30-week-old male and female WT mice were killed, the dorsal skin collected and a wound was created using a single 6-mm biopsy punch. Immediately after creating the wound the samples were placed into a Petri dish and incubated for 6 h at 37 °C. Subsequently, the samples were removed from the incubator and the wound edge as well as the unwounded control skin was collected with surgical scissors. These tissue samples were immediately flash frozen. Afterwards, RNA was extracted and RT–qPCR was performed as described below.

To determine in vivo cell proliferation, EdU was used. For live mice, 50 µl of the EdU solution (5 mg ml^−1^) was injected i.p. 3 or 6 h before killing.

For ex vivo experiments on mouse skin, the tissue was stretched over a Styrofoam lid using needles. A 6-mm biopsy punch was then used to create a wound, and the tissue was incubated at 37 °C under normoxic conditions for 6 h. After incubation, samples were collected from the wound edge, fixed in formaldehyde for histological processing, or flash frozen for RT–qPCR analysis.

### Experiments in pigs

Pig experiments were conducted in accordance with the ethical guidelines of the host institution, with the support of the veterinarian team from LBI Trauma. All experimental procedures received approval from the Municipal Government of Vienna, aligning with Austrian law and the NIH Guide for the Care and Use of Laboratory Animals (revised 2011).

The transportation of animals from the breeder to the LBI housing area adhered to legal animal transport guidelines, using the institute’s air-conditioned and temperature-monitored animal transporter. Trained personnel ensured compliance and allowed the animals time to acclimatize. Internally standardized guidelines were followed for housing, with ad libitum access to water and feed. Enrichment for pigs included straw, hay, rubber balls, an additional water bowl and sugar cubes. Animal care, overseen by a veterinarian, involved several daily check-ins by animal keepers to monitor their health. Approved protocols permitted non-recovery experiments involving biopsy and sample collection up to 10 h post-injury. Pigs were anaesthetized and intubated under standard procedures. Following, dorsal skin was shaved and wounds were induced with a 6-mm biopsy punch, the edges of these wounds were then collected at 15 min, 1.5 h, 3 h, 6 h, 8 h and 10 h. Skin samples were collected from a total of four male pigs (aged 12 weeks), concurrently utilized in other experiments. Samples were fixed in 10% formalin and embedded in paraffin for histological analysis.

For studying late-onset porcine wounds, six pigs underwent a surgery unrelated to this study, which required a central laparotomy incision into the abdominal cavity and was closed with resorbable sutures or staples. At 7 days post-surgery, differences in the healing process due to inter-animal variation were observed, even within the same injury. Two trained veterinarians provided an unbiased wound score for each position of the wound where a sample was taken, dividing samples into ‘healed’ and ‘healing’.

### Experiments on porcine skin ex vivo

Pig skin was collected immediately after killing the pig, placed into a Petri dish, and incubated for 1 h at 37 °C. A single 6-mm biopsy punch was then used to create a wound. The samples were subsequently incubated for 3 h at 37 °C. After this, 400 µl of EdU (5 mg ml^−1^ in PBS) was pipetted into the hole created by the biopsy punch. After adding EdU, the skin samples were immediately returned to the 37 °C incubator for another 3 h. Subsequently, the samples were removed and two biopsy samples were taken from the edges of the original biopsy. The tissue was then fixed in formaldehyde for 24 h.

For the experiments with transcription and translation inhibition, porcine skin ex vivo was wounded as described above. Immediately after creating the wound, various treatments (vehicle (PBS), transcription inhibitors (50 ng ml^−1^ actinomycin D (Sigma A9415-2MG) with 50 µg ml^−1^ α-amanitin in PBS) or translation inhibitors (1 mg ml ^−1^ cycloheximide (Sigma, C7698-1G) in PBS)) were applied to the biopsy punch hole. Other drug interventions ex vivo were performed using 53 inhibitor (Pifithrin-α; 200 µM), ATM inhibitor (KU55933; 15 µM), Rapamycin (55 µM) or Everolimus (500 nM). The samples were then incubated for an additional 1.5 h at 37 °C. Subsequently, the samples were removed from the incubator and two half-moon-shaped biopsies were extracted from the edges of the original wound site. These tissue samples were fixed in formaldehyde for 24 h.

### Western blot

Frozen pig skin samples were collected at the control site and 3 h after injury or upon a 1.5-h-long treatment of skin with vehicle (PBS) or transcription inhibitors (50 ng ml ^−1^ actinomycin D with 50 µg ml ^−1^ α-amanitin in PBS). Samples were powdered in liquid nitrogen using a prechilled mortar and pestle and then homogenized in RIPA buffer containing protease inhibitor cocktail (Sigma-Aldrich), phosphatase inhibitor cocktail (Sigma-Aldrich) and phenylmethanesulfonyl fluoride (PMSF; Sigma-Aldrich) followed by sonication (3 × 5 s). Total cell lysates were prepared in 100–400 μl RIPA buffer containing inhibitor cocktail (Sigma-Aldrich). Protein concentration was measured using BCA protein assay (Sigma-Aldrich). Then, 30 µg of protein were separated on 12% Bis–Tris gels (Thermo Fisher Scientific) and transferred to polyvinylidene difluoride membranes (Bio-Rad). The membranes were incubated overnight separately with anti-β-actin (1:1,000 dilution, 8226, Abcam), anti p21 (1:1,000 dilution, 188244, Abcam), anti-epigen (1:500 dilution, 5767R, Thermo Fisher Scientific) and anti-c-Fos (1:1,000 dilution, 190289, Abcam) followed by incubation with secondary antibodies against goat anti-mouse IgG (1:1,000 dilution, 91196, Cell Signalling) and goat anti-rabbit IgG (1:1,000 dilution, ABIN101990) antibodies conjugated to horseradish peroxidase (HRP) for 1 h. Bands were visualized using the ChemiDoc Touch Imaging System (Bio-Rad Laboratories) and analysed using Image Lab Software (Bio-Rad Laboratories) according to the manufacturer’s protocol.

### Histology

For the analysis of tissue samples, both immunohistochemistry (IHC) and immunohistofluorescence (IHF) techniques were employed. Tissue sections underwent deparaffinization and rehydration through a graded alcohol series. To retrieve antigens through heat induction, samples were incubated in a steamer at 95 °C for 20 min using 0.1 M Tris with 0.01 M EDTA (pH 9.0) or sodium citrate buffer 0.01 M (pH 6.0), or in the microwave where the buffer was brought to a boil and then left for 15 min at sub-boiling temperature.

For IHF, samples were blocked in PBS containing 0.4% bovine serum albumin (BSA; Sigma-Aldrich) and 1.6% normal goat serum (NGS; Vectorlabs) for 1 h at room temperature. Subsequently, samples were incubated with appropriate primary antibodies in blocking buffer overnight at 4 °C. Slides were washed three times with TBS-Tween (TBST) and incubated for 1 h with DAPI and a fluorescent secondary antibody (Thermo Fisher), after which they were mounted in MOWIOL (Carl Roth) mounting medium.

For IHC, samples were blocked for 10 min using BLOXALL (Vectorlabs), washed in TBST, and then incubated for 1 h at room temperature or overnight at 4 °C with a primary antibody. Following incubation, samples were washed three times, incubated with HRP-conjugated secondary antibody (BrightVision) for 30 min, and stained for 6 min with NovaRed (Vectorlabs). Finally, samples were counterstained with haematoxylin, dehydrated and mounted.

For histological analyses, various primary antibodies and dyes were employed as detailed below. The c-Fos antibody (mouse, Abcam ab208942) was used at a dilution of 1:1,000. F-actin was detected using Phalloidin-iFluor 488 (Abcam ab176753, 1:1,000 dilution). Fast myosin was labelled with a rabbit antibody (Abcam ab91506, 1:100 dilution), while keratins K10 and K14 were identified using a guinea pig antibody (Progen GP-K10, 1:400 dilution) and a mouse antibody (Abcam ab7800, 1:200 dilution), respectively. The proliferation marker Ki67 (Abcam ab15580, rabbit) was used at 1:1,000 dilution and Lamin B1 (Abcam ab16048, rabbit) at 1:400 dilution. For p21 detection, three antibodies were used: a rat antibody (Abcam ab107099, 1:100 dilution) and two rabbit antibodies (Abcam ab109520, 1:100 dilution and Abcam ab188244, 1:500 dilution). The p53 antibody (Abcam ab13144, rabbit) was applied at 1:100 dilution. Lipid droplet-associated Perilipin 2 was labelled using a guinea pig antibody (Progen GP40, 1:250 dilution) and Plet1 was detected with a rat antibody (Nordic MUBio MUB1512P, 1:50 dilution). The phosphorylated ribosomal protein p-rpS6 (S235/236) antibody (Cell Signalling 4858, rabbit) was used at a 1:200 dilution and tdTomato was stained with a goat antibody (Origene AB8181-200, 1:100 dilution). Vimentin was detected using a chicken antibody (Abcam ab24525, 1:1,000 dilution). Wheatgerm agglutinin (WGA) (W11261) was applied at 1:200 dilution and γ-H2A.X antibody (Cell Signalling 9718S, rabbit) at 1:200 dilution.

### RNA in situ hybridization

RNA in situ hybridization (ISH) was performed according to the Advanced Cell Diagnostics (ACD) RNAscope protocol. Paraffin sections were deparaffinized and rehydrated in ethanol. Sections were incubated for 10 min with hydrogen peroxide, then sections were placed in hot retrieval reagent and heated for 15 min. After two washes with the wash buffer (ACD), a hydrophobic barrier was drawn around the tissue section. The sections were treated with ‘protease plus’ solution for 30 min at 40 °C. This was followed by two washes and incubated with respective target probe for 2 h at 40 °C. Afterwards, slides were washed, followed by incubation with the amplification steps AMP1 (30 min at 40 °C), AMP2 (15 min at 40 °C), AMP3 (30 min at 40 °C), AMP4 (15 min at 40 °C), AMP5 (30 min RT), finally, AMP6 (15 min at RT) two washes of 2 min each were performed between each amplification step. Then, the RNAscope 2.5 HD Detection kit (RED) (Bio-Techne, 322360) was used. Afterwards IHF was performed or DAPI was added for 5 min and sections were mounted.

For RNA in situ hybridization (RNA-ISH), specific probes targeting both mouse and pig transcripts were used. The *Cdkn1a* probe for *Mus* *musculus* (Bio-Techne, 408551) and the *Cdkn1a* probe for *Sus scrofa* (Bio-Techne, 845281) were employed to detect expression of the *Cdkn1a* gene across species. Additional mouse-specific probes included *Epgn* (Bio-Techne, 437971), *Igfbp3* (Bio-Techne, 405941) and *Adam8* (Bio-Techne, 1047421-C1). All probes were obtained from Bio-Techne and used according to the manufacturer’s instructions.

### Oil Red O and F-actin staining

For these experiments, murine wounds 3 days post-injury were preserved in OCT using dry ice. Sections were then cut with a cryotome with a thickness of 8 μm and placed onto a histology slide. Sections were kept for 15 min at room temperature and then fixed for 10 min in 2% paraformaldehyde dissolved in PBS, followed by two washes for 5 min in TBST. A hydrophobic barrier was drawn around the tissue section.

For F-actin staining slides were incubated for 90 min with 100 µl of DAPI and Phalloidin-488. For Oil Red O (ORO) staining, samples were first stained with IHF against Plin2, nuclei were stained with DAPI, and sections were incubated in 0.3% ORO (cat no. 26125) solution for 15 min, washed with distilled water and mounted.

### Measurements of proliferation using EdU

Fluorescent detection of EdU was performed using the EdU Click-488 kit (Roth, 7773.1). Following deparaffinization and antigen retrieval, tissue sections were stained for EdU following the manufacturer’s guidelines. The IHF staining was then completed as described above.

### RT–qPCR

Tissue samples (50–100 mg, kept frozen) were homogenized in 1 ml of TRIzol with ceramic beads in a bead mill at 30 rps for up to 30 min until the tissue was homogenized. If the tissue was not homogenized after 30 min, the samples were flash frozen in liquid nitrogen and returned to the bead mill for another cycle. Then, 200 µl of chloroform was added to the samples, they were shaken, incubated for 3 min at room temperature and centrifuged at 12,000*g* for 15 min at 4 °C. The RNA layer was transferred to a fresh Eppendorf tube and 500 µl of isopropanol added, samples were shaken, incubated for 10 min at room temperature and centrifuged at 12,000*g* for 10 min at 4 °C. The supernatant was discarded, and the pellet was washed with 1 ml of 70% ethanol and centrifuged at 7,500*g* for 5 min at 4 °C. Finally, the pellet was allowed to dry at room temperature before being resuspended in distilled water.

The RNA concentration and quality were determined by spectrophotometry. Subsequently, 2 µg of the RNA samples were treated with DNase I (Promega kit) and incubated at 37 °C. Post-digestion, the RNA was cleaned using the RNeasy MinElute Cleanup kit (QIAGEN). The purified RNA was reverse transcribed using the OneScript Plus cDNA Synthesis kit in a 20-µl reaction volume. RT–qPCR was performed with either the Biozym Blue S’Green qPCR Mix Separate ROX kit or PrimeTime One-Step RT–qPCR Master Mix for a Taqman assay. Primers used for the application of specific genes are listed below. Data were analysed using qPCRSoft v.4.1 and Microsoft Excel, focusing on the mean of triplet measurements. Relative expression was calculated using the 2^−ΔΔCt^ method, comparing target gene Ct values to the *Gapdh* housekeeping gene.

Primers used were as follows: *Adam8* (mouse) F: 5′-CAAAGATACCAACCTGAATGACC-3′, R: 5′-GCAACCACATACAACTCCAC-3′; *Ccl27a* (mouse) F: 5′-GATGTCTCCAACAAGCCAG-3′, R: 5′-TTCTAACCACCGAGCCAG-3′; *Cdkn1a* (mouse) F: 5′-AGGATTGGACATGGTGCCTG-3′, R: 5′-CGGTGTCAGAGTCTAGGGGA-3′; *Cxcl16* (mouse) F: 5′-CGTTGTCCATTCTTTATCAGGT-3′, R: 5′-GGGTGTGCTCTTTGTTTAAGG-3′; *Epgn* (mouse) F: 5′-CAAAGCAATGAAGGCAGCAC-3′, R: 5′-CCACAGCATACGAAGTTAGGG-3′; *F3* (mouse) F: 5′-CGATTGATGTGGAAGAAGGAG-3′, R: 5′-CAGGAGGATGATAAAGATGGT-3′; *Fcgbp* (mouse) F: 5′-CTACCCAGACAATTTCGCCAG-3′, R: 5′-CCATCTATCAGCACTCTTCCAG-3′; *Fgf7* (mouse) F: 5′-ATAGAAACAGGTCGTGACAAGG-3′, R: 5′-CAGACAGCAGACACGGAAC-3′; *Gapdh* (mouse) F: 5′-CTGAGTATGTCGTGGAGTC-3′, R: 5′-GGATGCATTGCTGACAATC-3′; *HPRT* (pig) F: CAGAGGGCTACGATGTGATG, R: CCAGCGTCGTGATTAGTGAT; *Cdkn1a* (pig) F: GAGAAGGGGGCATGCTAGAC, R: AGTTCCAGGCGTTGATTGGT; *Igfbp3* (mouse) F: 5′-GAGTGACCGATTCCAAGTTCC-3′, R: 5′-CAGGGACCGTATTCTGTCTC-3′; *Il6* (mouse) F: 5′-GTTCTCTGGGAAATCGTGGA-3′, R: 5′-GCATTGGAAATTGGGGTAGG-3′; *Mdk* (mouse) F: 5′-GCAACTGGAAGAAGGAATTTGG-3′, R: 5′-CTTTCTTGGCTTTGGTCTTTGAC-3′; *Serpinb2* (mouse) F: 5′-CATACCTGTCCAGATGATGTTCC-3′, R: 5′-ACTTTCCAGCAATTCCAAGCC-3′; *Sfn* (mouse) F: 5′-CCGTCTGTCTGTCCATCCT-3′, R: 5′-CCTCCTCGTTGCTCTTCTG-3′; *Thbs1* (mouse) F: 5′-AAACCGATTTCCGACAATTCC-3′, R: 5′-CATCATAACCTACAGCAAGTCC-3′; *TdTomato* (TaqMan) Probe: 5′-/56-FAM/CCGCCACCA/ZEN/CCTGTTCCTGT/3IABkFQ/-3′, F: 5′-CTTGTACAGCTCGTCCATGC-3′, R: 5′-ACCTCCCACAACGAGGACTA-3′; *Cdkn2a* (TaqMan) Probe: 5′-/56-FAM/TGCACCGTA/ZEN/GTTGAGCAGAAGAGC/3IABkFQ/-3′, F: 5′-GAACTCTTTCGGTCGTACCC-3′, R: 5′-GAGAAGGTAGTGGGGTCCT-3′; (Mm.PT.58.42804808 assay ID).

### Creation of the sc-RNA-seq atlas

The single-cell RNA sequencing (sc-RNA-seq) mouse skin atlas was created using data from 20 publicly available studies (cited in the main manuscript file), which utilized the Chromium Single Cell 3′ Reagent kit Version 2 (10x Genomics). Raw data were processed using the standard pipeline Drop-Seq (v.1.2) with some modifications. Raw read 1, bases 1–16 were tagged with cell barcode XC, and bases 17–26 were tagged with UMI XM. Read 2 was trimmed at the 5′ end to remove any adaptor sequence and at the 3′ end to remove poly(A) sequences of length six or more, followed by alignment to the mouse (mm10) genome reference using the STAR aligner (STAR_2.5.1a), allowing no more than three mismatches. The gene expression matrix was then generated using a ‘MIN_BC_READ_THRESHOLD = 2’ parameter. In our workflow, we employed the scDblFinder package^104^ for identifications of doublets. In our analysis, 2–6% of cells were identified as doublets based on their doublet score and were removed before further analysis. Next, the gene expression matrix for each sample was processed for quality control, mitochondrial genes, ubiquitously expressed ribosomal protein-coding (RPS and RPL) and MALAT1 and other noncoding RNA genes including miRNAs. Finally, filtered individual samples were merged into a combined expression matrix. Gene expression and cell type identification were performed using Seurat V4.xx^105^. Cells with >25% mitochondrial content were considered of poor quality and removed from the analysis. The data was then scaled (ScaleData), variable genes were identified (FindVariableGenes) and the top 2,000 variable genes from the combined objects were used to perform further analysis.

### Analysis of the sc-RNA-seq atlas

Data were analysed with more granularity for major cell types (for example, fibroblasts, immune cells, endothelial cells, keratinocytes and melanocytes) and a subset of keratinocytes, fibroblasts, immune cells and smooth muscle cells was used for further analysis. Furthermore, with the exception of age range of the atlas and the comparison in p16 between adults and aged fibroblasts, the analysis was limited to samples collected from adult animals. The Seurat R package was employed for data normalization and feature selection. Data normalization was executed using the logNormalize method with a scale factor of 10,000. Feature selection was carried out to identify the top 2,000 variable features using the variance-stabilizing transformation method. Subsequent principal-component analysis (PCA) was conducted to reduce the dataset to 50 principal components, aiding in the capture of the most pertinent features. The PCA process was initialized with a set seed to ensure reproducibility. To visualize the distribution of principal components and feature counts across different study and samples we used dimensional heatmaps and violin plots. To mitigate batch effects, Harmony integration was applied. This method enabled the alignment of datasets from disparate sources, refining the overall data quality. The process generated Harmony embeddings, which were subsequently visualized using dimension plots (DimPlots) and violin plots (VlnPlots), categorized by study ID, cell type, wound class and age class. The intrinsic dimensionality of the dataset was estimated using the maxLikGlobalDimEst function from the intrinsicDimension R package. An elbow plot was constructed to assess the s.d. across the principal components, assisting in the determination of the number of significant components. Clusters were identified using the Harmony-reduced data through neighbour finding, cluster determination and UMAP reduction. A cluster tree was constructed to elucidate the relationships among the identified clusters, offering insights into the underlying data structure. Afterwards a UMAP plot was generated for an overview of the generated data clusters. To identify clusters with the highest expression of *Cdkn1a* (p21) the average expression of *Cdkn1a* in each cluster was calculated. To further explore the characteristics of the clusters the expression patterns of genes of interest were visualized using violin plots. Specifically, in the analysis of keratinocytes, the expression of various keratins such as K5, K10, K2 and Ivl was explored in the clusters. Afterwards, differential gene expression analysis was performed by comparing the clusters with the highest average *Cdkn1a* expression with the rest of the clusters. The differentially expressed genes with a log fold change (FC) > 1 were used in a pathway-enrichment analysis using GO and Kyoto Encyclopedia of Genes and Genomes pathways. Results of the enrichment analysis were visualized using dotplots and network plots. Additionally, immune cells were categorized into different subtypes using the ScType pipeline (https://github.com/IanevskiAleksandr/sc-type) to observe the expression of senescent markers. A graph was constructed that depicts the expression of Cdkn2a for each sub-type of the immune cells.

### Histological data analysis

Unless specified differently, the analysis of senescence features of epidermal cells was conducted in 100 μm of epidermis, the most proximal to the injury site, and control was a region at least 1 mm away from the wound site or an unwounded skin. For the dermal compartment, an area of 100 μm^2^ at the wound site (injury) or at least 1 mm away from it on skin of unwounded animals (controls) was used. Regarding the ALISE phenotype, cells were classified as positive if they contained more than five PLIN2^+^ vesicles. To quantify the intensity of Lamin B1 (LMNB1), the analysis encompassed 50 p21^+^ and 50 p21^−^ cells, where mean signal intensity of Lmnb1 at the nuclear envelope were measured by selecting several points of measurements around the envelope. For the analysis of epigen mRNA (*Epgn*) cells containing more than five individual foci/mRNA transcripts were classified as positive. For the EdU quantification, only keratinocytes of stratum basale were counted. The quantification region extended from the tip of the epidermal tongue to the last p21^+^ keratinocyte. Subsequently, the percentage of EdU^+^ cells within the total populations of both p21^−^ and p21^+^ cells was calculated. For the analysis of the relationship between DNA damage and p21, *Z*-stack images were obtained at the wound edges and *Z*-Max projections were generated. Cells were deemed positive for γ-H2A.X if they had more than ten foci. For this analysis, the area within the epidermal tongue, from the tip to the last p21^+^ cell, was quantified for the total number of keratinocytes as well as p21^+^ and γ-H2A.X^+^ keratinocytes. Additionally, approximately 80 keratinocytes in the homeostatic part of the epidermis were quantified for the presence of γ-H2A.X. For analysis of γ-H2A.X in porcine skin, three regions of 0–40 µm from the edge of the wound, 40–140 µm from the edge of the wound and 100 µm in the unwounded region of the skin sample were quantified.

For the analysis of spatial positioning of p21^+^ cells in the epidermal tongue, the DAPI (blue) and the p21 (red) channels were separated. Subsequently, the blue channel was duplicated, and a threshold was set to create a binary mask. This mask was used to subtract the intensity of the red channel inversely, effectively reducing the red channel signal outside the cell nuclei to zero while preserving the original intensities within the nuclei. The epidermal tongue was then identified and isolated. All red channel signals outside the epidermal tongue area were removed to focus the analysis on the epidermal tongue. The epidermal tongue was segmented into 100-µm segments (measured at the centre of its thickness), starting from the tip and extending to the last identifiable p21^+^ keratinocyte. Within each segment, the total number of p21^+^ cells and nuclei were counted, and the proportion of p21^+^ cells was calculated. Finally, the proportion of p21^+^ cells in each segment was correlated with their spatial positions, and a Pearson’s correlation coefficient was calculated.

For the analysis of EdU^+^ cells in porcine skin ex vivo, sections were stained for EdU, p21 and DAPI, and then scanned with an immunofluorescent microscope (TissueFAXS i PLUS; TissueGnostics). Specifically, 1 mm of the epidermis from the wound edge was divided into five 200-µm segments. Within each segment, the proportion of EdU^+^ and p21^+^ cells relative to the total number of nuclei within that segment was quantified. Subsequently, a graph was created, showing the distance from the wound on the *x* axis and the proportion of p21^+^ or EdU^+^ cells on the *y* axis in each 200-µm segment. To determine the proportion of EdU^+^ keratinocytes in mouse skin in vivo, the epidermis was segmented into three sections from the wound edge at 500 µm, 1,250 µm and 500 µm. The proportion of EdU^+^ keratinocytes was quantified in the first 500 µm (proximal) and the second 500 µm (distal) sections.

For the relationship between transcript and protein increase upon injury, porcine skin, ex vivo samples were generated and treated as described above. The subsequent sections were stained against c-Fos, p21 and DAPI, imaging was performed using an immunofluorescent microscope and the captured data were analysed using ImageJ software. Specifically, the analysis focused on quantifying the total number of p21^+^ and c-Fos^+^ keratinocytes in the epidermis near the wound edge. Finally, a comparative graph was created to showcase the total count of p21^+^ and c-Fos^+^ keratinocytes across each treatment category of vehicle, transcription inhibition and translation inhibition.

For assessment of the quantity of p21^+^ cells after AP or UC2288 treatment, IHC staining against p21 was used. Images were imported into QuPath. Cells were detected using the ‘Cell Detection’ algorithm. Positive cells were identified based on an intensity threshold. The region of interest was defined as 3,000 pixels in length along the epidermal tongue for epidermal cells and 100 μm^2^ for dermal cells. In this region, the proportion of p21^+^ keratinocytes was calculated in relation to the total number of cells within that region.

Similarly, for assessment of the quantity of pSTAT3^+^ cells after UC2288 treatment, IHF staining against pSTAT3 was used. Images were imported into QuPath. Cells were detected using the ‘Cell Detection’ algorithm. Positive cells were identified based on an intensity threshold. The region of interest was defined from the tip of the epidermal tongue to a point where the epidermal thickness was twice that of the distal unwounded region. In this region, the proportion of pSTAT3^+^ keratinocytes was calculated in relation to the total number of cells within that region.

Export of *Cdkn1a* mRNA was assessed using RNA in situ hybridization (RNA-ISH) for *Cdkn1a* mRNA and WGA staining. Following the staining, *Cdkn1a* mRNA nuclear retention was evaluated by analysing ×40 magnification images of control and wounded porcine skin. The region of interest of epidermis was isolated from these images and a mask of nuclei was created. The area occupied by *Cdkn1a* mRNA particles was then measured using the particle analysis tool in ImageJ. The mask of nuclei was used to quantify the area of *Cdkn1a* mRNA particles within the nuclei of epidermal cells. The inverted mask of nuclei (representing the epidermal region excluding nuclei, approximately corresponding to the cytoplasm) was used to measure the area of *Cdkn1a* mRNA particles in the cytoplasm of epidermal cells. Finally, the ratio of *Cdkn1a* mRNA particle areas in the cytoplasm to the nucleus was calculated.

The length and curvature of the epidermal tongue were measured using the kappa curvature tool, starting from the tip of the epidermal tongue to the point where the panniculus carnosus was cut in the dermis. To identify this point in the epidermis, a line was drawn perpendicular to the panniculus carnosus and extended beyond the epidermis. Histological analysis of depth of the p-rpS6-zone was performed in OlyVIA software (Olympus Corporation). For this analysis the depth of the p-rpS6-zone was measured on both sides of the wound of each animal in epidermis and dermis. For each animal the average of these measurements was used and compared between mice treated UC2288 or vehicle.

### *Cdkn1a* pulldown experiment

The RNA affinity purification was performed following the protocol described previously^67^, with a few modifications. Three different types of pig skin samples were collected: control skin, biopsy punches from the proximity of excision wounds and microneedled skin areas. Based on a histological analysis, we calculated that approximately 50 biopsy punches (4 mm in diameter) would be needed to obtain 500 million p21^+^ keratinocytes.

For the generation of wounded skin samples, pig skin was subjected to either excision injury with a 4-mm biopsy punch or microneedled 50 times at a depth of 3 mm using a DermaStamp. Excision wound samples were generated by collecting a 6-mm biopsy punch from around the excision wound, resulting in donut (ring)-shaped samples representing ~1 mm of tissue around the injury site. All wounded skin samples were collected 1.5 h post-injury. Control and microneedled skin samples were collected using a 4-mm biopsy punch. All the biopsies were removed using tweezers and sharp scissors. The biopsy was washed in PBS, and the dermal layer was trimmed away as much as possible with a razor blade. Each biopsy punch was then immediately fixed in 4% formaldehyde for 6 h. After the fixation, the samples were washed three times with 50 ml of chilled PBS. Before freezing, excess liquid was removed by pipetting and dabbing the samples with a paper towel. The samples were then flash frozen and stored at −80 °C until further use.

To prepare samples for the pulldown experiment, a metal mortar and pestle were cooled with liquid nitrogen. Fifty biopsy samples from each group were added to the liquid nitrogen in the mortar, covered with a plastic lid and crushed with the metal pestle. Liquid nitrogen was added periodically to keep the samples frozen during crushing. Once reduced to a fine powder, the samples were transferred to a 50-ml Falcon tube containing lysis buffer (50 mM Tris-HCl, pH 7.0, 10 mM EDTA, 1% SDS, 1:200 dilution phenylmethyl sulfonyl fluoride (PMSF; Sigma-Aldrich, 93482-50ML-F), 1:100 dilution protease inhibitors (Sigma-Aldrich, P8340-5ML) and 1:100 dilution Superase-In (Thermo Fisher, AM2696)) at a volume ten times the mass of the sample. The samples were placed on ice and homogenized using a homogenizer (Janke & Kunkel IKA-Labortehnik, Ultra Turrax T25). Each sample was homogenized six times for 1 min at maximum speed, with centrifugation between each homogenization step. The samples were then sonicated (Bandelin Sonoplus mini20) at 40% maximum energy, 20 times for 1 min, with at least 1 min of rest between each sonication step. Samples were kept on ice at all times throughout the sonication process. After sonication, the samples were transferred to 2-ml Eppendorf tubes and incubated on ice for 45 min. The samples were then centrifuged at 16,100*g* for 10 min at 4 °C. The supernatant was collected into 15-ml Falcon Protein LoBind tubes, and each sample was pre-cleared with Dynabeads MyOne Streptavidin C1 (Thermo Fisher, 65002) with 30 µl of beads per 1 ml of supernatant. In brief, samples were incubated with the C-1 magnetic beads at 37 °C with gentle shaking and the beads were removed twice using a DynaMag-2 Magnet. Subsequently, 2 ml of hybridization buffer (750 mM NaCl, 1% SDS, 50 mM Tris-HCl, pH 7.0, 1 mM EDTA, 1:200 dilution PMSF, 1:100 dilution protease inhibitors and 1:100 dilution Superase-In) per 1 ml of sample and 260 pmol of raPOOL probe were added to each sample (Supplementary Table 5). The samples were incubated overnight at 37 °C with gentle shaking. The C-1 magnetic beads were resuspended in lysis buffer and 100 µl of beads per 100 pmol of raPOOL probes was added to the samples. After 30 min of incubation at 37 °C, the magnetic beads were collected on a magnetic stand, the supernatant was discarded, and the beads were resuspended in 1 mL of preheated wash buffer (2× NaCl and sodium citrate (SSC), 0.5% SDS and 1:200 dilution PMSF) at 37 °C. The resuspended beads were transferred to a fresh 1.5-ml Eppendorf tube and incubated for 7 min at 37 °C with gentle shaking, followed by bead collection using a magnetic stand. This washing step was repeated five times. After the final wash, the beads were resuspended in elution buffer (12.5 mM biotin (Invitrogen), 7.5 mM HEPES (pH 7.5), 75 mM NaCl, 1.5 mM EDTA, 0.15% SDS, 0.075% sarkosyl and 0.02% Na-deoxycholate). The samples were incubated for 20 min at room temperature on an orbital shaker, followed by a 10-min incubation at 65 °C. The beads were removed using a magnetic stand and the eluted samples were incubated at 65 °C overnight for de-crosslinking. Finally, the samples were used for RT–qPCR and sent for proteomics analysis.

RT–qPCR was used to assess the specificity of the pulldown, and for this assay 80 µl of the final eluate from each sample was used. In brief, 400 µl of TRIzol was added to each sample and the mixture was vortexed for 10 s, followed by a 10-min incubation at room temperature. Afterward, 100 µl of chloroform was added to the TRIzol-treated samples and thoroughly mixed. The samples were then centrifuged at 16,100*g* for 15 min at 4 °C. Following centrifugation, the upper aqueous phase (~400 µl) was carefully removed and transferred to a fresh 1.5-ml Eppendorf tube. To the supernatant, 600 µl (1.5× volume) of 100% ethanol was added and mixed well. The remaining cleanup steps followed the manufacturer’s protocol for the MIRNeasy Mini kit. Samples were eluted from the spin columns with 20 µl of nuclease-free H_2_O. DNA digestion RNA reverse transcription and RT–qPCR were performed as described in the previous section of the Methods. The sequences of primers with an annealing temperature of 60 °C were used for the RT–qPCR amplification are described in ‘RT–qPCR’.

To visualize the purity of the pulldown, cDNA gel electrophoresis was performed. In brief, 2% agarose gel was prepared by weighing 0.6 g of agarose powder in 30 ml of SB buffer in a glass beaker. The beaker was covered with aluminium foil and then heated in the microwave until the boiling point, and kept at the boiling temperature until all of the agarose powder was dissolved. Afterwards, 1.5 µl of ethidium bromide was added to the solution and mixed well. The agarose gel was poured into a mould and left at room temperature for 20 min to cool down. The gel was then transferred to an electrophoresis bath. To 10 µl of RT–qPCR product, 2 µl of loading dye was added, mixed well and loaded onto the gel. A low-range ladder was added into the gel for size reference. The samples were run for 90 min at 70 V for chromatographic resolution and then imaged.

Proteomics was performed by the Max Perutz Labs, Mass Spectrometry Facility. The proteomics results were analysed by removing hits that are not above LacZ control levels (log_2_ ratio to LacZ < 0.5), removing hits not matching the criteria of the quality filter and endogenously biotinylated proteins. Hits were considered relevant if the same pattern of changes (in relation to their proper controls) was observed for two modalities of wounding (excision wounds and microneedling). GO terms analysis was performed as described for the sc-RNA-seq analysis.

### Bioinformatic analysis of SRSF3 and hnRNPA1 to *Cdkn1a*

Motif analyses were performed using the ATtRACT database as the source of experimentally validated RNA‑binding protein. Position‑weight matrices for SRSF3 and hnRNPA1 were extracted and converted into a standardized four‑row format for downstream scanning. These motifs were then mapped across the full *Cdkn1a* (p21) transcript, including the 5′ UTR, CDS and 3′ UTR, to quantify motif density and positional enrichment. To assess evolutionary conservation, multiple sequence alignment was performed SRSF3 and hnRNPA1 top‑hit binding using *Cdkn1a* and its orthologues. Multi‑species orthologues were downloaded from NCBI and aligned using MAFFT (v.7)^106^ with the default parameters. For each motif, the corresponding aligned block was identified in the mouse reference sequence and the equivalent region was retrieved from the alignment. Sequence logos were generated from ungapped motif blocks to visualize base conservation and motif variability across mammals, highlighting conserved and divergent nucleotide positions within the aligned motif context

### (Bulk) proteomics of excisional wounds and its analysis

Preparation of the samples for proteomics was performed as in the pulldown experiment of *Cdkn1a* mRNA (see ‘*Cdkn1a* pulldown experiment’ section).

For proteomic analysis of porcine excision injury samples, tissue was collected from 3–4 pigs. In each animal, circular 6-mm full-thickness excision wounds were created, and the surrounding tissue was sampled using 8-mm punch biopsies at three time points of immediately after wounding (control; *n* = 4), 15 min (‘short’; *n* = 4) and 4 h (‘long’; *n* = 3). For each condition in each pig, two 8-mm biopsies were taken adjacent to the wound margin. Biopsies were briefly rinsed in ice-cold PBS and snap-frozen in liquid nitrogen immediately after collection, then stored at −80 °C until lysis.

Tissue lysates for proteome analysis were alkylated and digested with LysC and trypsin (overnight, 37 °C). Resulting peptides were desalted, quality-controlled by HPLC-UV and labelled with TMT (TMT11). Labelling efficiency was verified by LC–MS, reactions were quenched and all channels were combined. Equal mixing was assessed by LC–MS and volumes were adjusted as needed. The pooled sample was fractionated by high-pH reversed-phase chromatography into proteome fractions. Each fraction was analysed on an Orbitrap Exploris 480 (approximately 3 h per run).

Raw files were searched with MaxQuant (v.2.1.4.0) against2022.04_UP000008227_9823_Sus_scrofa(Pig)_1protein_per_gene.fasta. Searches used MS2 reporter ions with TMT11 isobaric labels and trypsin/P specificity, allowing up to two missed cleavages. Variable modifications were oxidation (M), protein N-terminal acetylation and deamidation (N,Q); carbamidomethylation (C) was set as a fixed modification. Results were filtered at 1% peptide-spectrum match protein FDR and reverse hits were removed. Data processing was performed in R. After removing contaminants and proteins with missing quantification values, statistical analysis was conducted with LIMMA to estimate FC, *P* values and multiple-testing-adjusted *P* values, including batch correction with pig as a blocking factor.

Bioinformatics analyses were performed in Python. Volcano plots were generated from per-condition log_2_ ratios with Benjamini–Hochberg-adjusted *P* values; significance was defined as adjusted *P* value < 0.05 with effect-size thresholds FC > 1.333 or FC < 1/1.333. PCA was performed on log_2_-transformed intensity values; data were scaled before PCA to emphasize variance and visualize separation of experimental conditions along the principal components. Gene set enrichment analysis was conducted separately for upregulated and downregulated protein sets to distinguish their association with biological processes. Over-representation was assessed with Fisher’s exact tests in Python and multiple testing was controlled using Benjamini–Hochberg procedure to report adjusted *P* values. Bubble plots summarized GO Biological Process terms (GO, 2025): the *x* axis shows the GeneRatio *k*/*K* (where *k* is the number of test genes annotated to a term and *K* is the size of the upregulated or downregulated set), the bubble size encodes the hit count (*k*) and the colour encodes the adjusted *P* value. Terms were ranked and the top GO Biological Process (2025) categories were shown, enabling direct comparison of dominant pathways in upregulated and downregulated gene sets.

### SRSF3 RIP–qPCR

RNA immunoprecipitation from fixed pig skin was performed using an SRSF3-specific antibody. In brief, control skin and excision wound samples (ring-shaped biopsies representing ~1 mm of tissue surrounding the injury site) were collected 1.5 h post-injury, fixed in 4% formaldehyde for 6 h, washed in chilled PBS, flash frozen and stored at −80 °C.

Frozen biopsies were cryo-pulverized (as described in the *Cdkn1a* pulldown/ChIRP‑MS experiment) and transferred to 5-ml tubes. Per biopsy, 200 μl RIP lysis buffer (50 mM Tris-HCl, pH 7.4–7.5, 150 mM NaCl, 0.5% NP-40, 0.1% SDS, 1 mM EDTA, 1:200 dilution PMSF (Sigma-Aldrich, 93482-50ML-F), 1:100 dilution protease inhibitors (Sigma-Aldrich, P8340-5ML) and RNase inhibitor (Promega RNasin N2511)) was added. Lysates were incubated on ice for 30 min, homogenized and sonicated (as described in *Cdkn1a* pulldown/ChIRP‑MS experiment), incubated at 65 °C for 30 min for de-crosslinking, clarified by centrifugation 17,000*g* for 15 min at 4 °C, quantified by Pierce 660 (Thermo Scientific, A55864), and pre-cleared with Protein A magnetic beads (Invitrogen, Dynabeads Protein A 10002D) for 1 h at 4 °C. To degrade DNA, samples were incubated with 2 μl Turbo DNase (Invitrogen, AM2238) at 37 °C for 5 min.

Protein A beads were washed 3× in 500 µl RIP lysis buffer. Antibody coupling was performed by incubating beads with 30 µg with SRSF3 antibody (Abcam, #ab198291) per 100 µl beads overnight at 4 °C with rotation, followed by one wash and resuspension in RIP lysis buffer. For each sample, 5% of the pre-cleared lysate was reserved as input and stored at −80 °C. The remaining lysate was incubated with antibody-coupled beads overnight at 4 °C with rotation. In parallel, a no-antibody control immunoprecipitation was performed by incubating lysate with beads that had undergone the same handling in the absence of antibody. Beads were washed on a magnetic rack three times with low-salt wash buffer (50 mM Tris-HCl, pH 7.4–7.5, 150 mM NaCl, 0.1% SDS, 0.5% NP-40 and 1 mM EDTA), twice with high-salt wash buffer (50 mM Tris-HCl, pH 7.4–7.5, 500 mM NaCl, 0.1% SDS, 0.5–1% NP-40 and 1 mM EDTA), followed by a wash in 1× proteinase K (PK) buffer and resuspension proteinase K solution (0.5 mg ml^−1^ proteinase K in 1× PK buffer) and incubated at 37 °C for 45 min. Subsequently, 200 µl of 2× PK buffer (100 mM Tris-HCl, 50 mM NaCl, 10 mM EDTA and 0.4% SDS) was added and samples were incubated at 37 °C for 30 min (1,000 rpm; Eppendorf Thermomixer Comfort 5355). Crosslinks were reversed by incubating eluates (and appropriately diluted inputs) at 65 °C overnight (300 rpm; Eppendorf Thermomixer Comfort 5355).

RNA was purified by phenol:chloroform:isoamyl alcohol extraction (25:24:1), followed by a chloroform:isoamyl alcohol (24:1) re-extraction. RNA was precipitated by addition of 3 M sodium acetate (50 µl), glycogen (1 µl) and 1:1 ethanol:isopropanol (1 ml), incubated overnight at −80 °C, pelleted at 17,000*g* for 15 min at 4 °C, washed sequentially with 80% and 75% ethanol, air-dried and resuspended in 20 µl RNase-free water. For low-yield IP samples, cDNA was synthesized using qScript Ultra SuperMix (Quantabio) according to the manufacturer’s instructions. qPCR was performed using gene-specific primers (listed in ‘RT–qPCR’) at an annealing temperature of 61 °C and a SYBR Green-based detection chemistry on a real-time PCR system (qTOWER^3^). Each reaction contained 4 µl cDNA per well. Ct values were obtained from technical qPCR replicates and averaged per sample for downstream calculations. Enrichment of target mRNA in RIP was expressed as % input, correcting for both the fraction of lysate reserved as input (5%) and the fraction of extracted RNA represented per qPCR well (due to RNA volume used for reverse transcription and final cDNA dilution). The % of mRNA target in the IP, normalized to the total RNA of the input was computed as such: %Input = 100 × 1.98^(Ct^_Input,adj_^−Ct^_IP_^)^.

### Statistics and reproducibility

All statistical analyses including testing the normality of data distribution were performed using GraphPad Prism v.9.3.1 and a *P* value < 0.05 was considered as significant. In all graphs, *P < 0.05, **P < 0.01, ***P < 0.001 and NS is not significant. For differences between two groups paired or unpaired two-tailed *t*-test was used, data were further tested for equality of variances using an *F* test. For more than two group comparisons, one-way ANOVA with Dunnet’s or Tukey’s multiple comparison test was used. For analysis concerning more than one variable two-way ANOVA with Sidak’s multiple comparison test or multiple *t*-test was used. Correlations were assessed using Pearson’s rank correlation test.

No statistical methods were used to predetermine sample sizes but our sample sizes are similar to those reported in previous publications^8,9,13,63^. No data were excluded from the analyses. The animals in experiments were randomized. The investigators were blinded in the experiments.

### Software

The software used in this study include Fiji (ImageJ2), an image processing software (v.2.9.0); GraphPad Prism (v.9.3.1) by GraphPad Software and OlyVIA (Olympus, RRID: SCR_016167), a virtual slide microscope software. QuPath (v.0.4.2) was employed as an open-source platform for digital pathology image analysis. For statistical computing and graphical representation, R (v.4.3.1) was utilized alongside RStudio (v.2023.06.1), an integrated development environment for the R programming language. Additionally, Python (CPython v.3.13.5) by the Python Software Foundation was used for data analysis and processing.

### Reporting summary

Further information on research design is available in the Nature Portfolio Reporting Summary linked to this article.

## Online content

Any methods, additional references, Nature Portfolio reporting summaries, source data, extended data, supplementary information, acknowledgements, peer review information; details of author contributions and competing interests; and statements of data and code availability are available at 10.1038/s41556-026-01948-2.

## Supplementary information

Supplementary Information Supplementary Figs. 1–5, figure legends and legends for Supplementary Tables 1–7.

Reporting Summary

Peer Review file

Supplementary Table 1 Supplementary Table 1.

Supplementary Table 2 Supplementary Table 2.

Supplementary Table 3 Supplementary Table 3.

Supplementary Table 4 Supplementary Table 4.

Supplementary Table 5 Supplementary Table 5.

Supplementary Table 6 Supplementary Table 6.

Supplementary Table 7 Light microscopy reporting table.

## Source data

Source Data All Unprocessed western blots and/or gels.

Source Data Statistical Source Data.

## Data Availability

The mass spectrometry proteomics data have been deposited to the ProteomeXchange Consortium via the PRIDE^107^ partner repository with the dataset identifier identifiers PXD075222 and PXD075230. Data supporting the findings of this study are available from the corresponding author on reasonable request. Source data are provided with this paper.
