# Supplementary material for: Transcription-independent induction of rapid-onset senescence is integral to healing
Source: Nat Cell Biol. 2026 May 28;28(6):1281–99. doi: 10.1038/s41556-026-01948-2 (PMC13279267; doi:10.1038/s41556-026-01948-2)
Supplement: Supplementary file 1 — Supplementary Figs. 1–5, figure legends and legends for Supplementary Tables 1–7. [file 41556_2026_1948_MOESM1_ESM.pdf]

nature cell biology

Supplementary information https://doi.org/10.1038/s41556-026-01948-2

Transcription-independent induction of rapid-onset senescence is integral to healing

In the format provided by the authors and unedited

SUPPLEMENTARY INFORMATION

SUPPLEMENATARY DATA FIGURES AND LEGENDS

Supplementary Data Figure 1

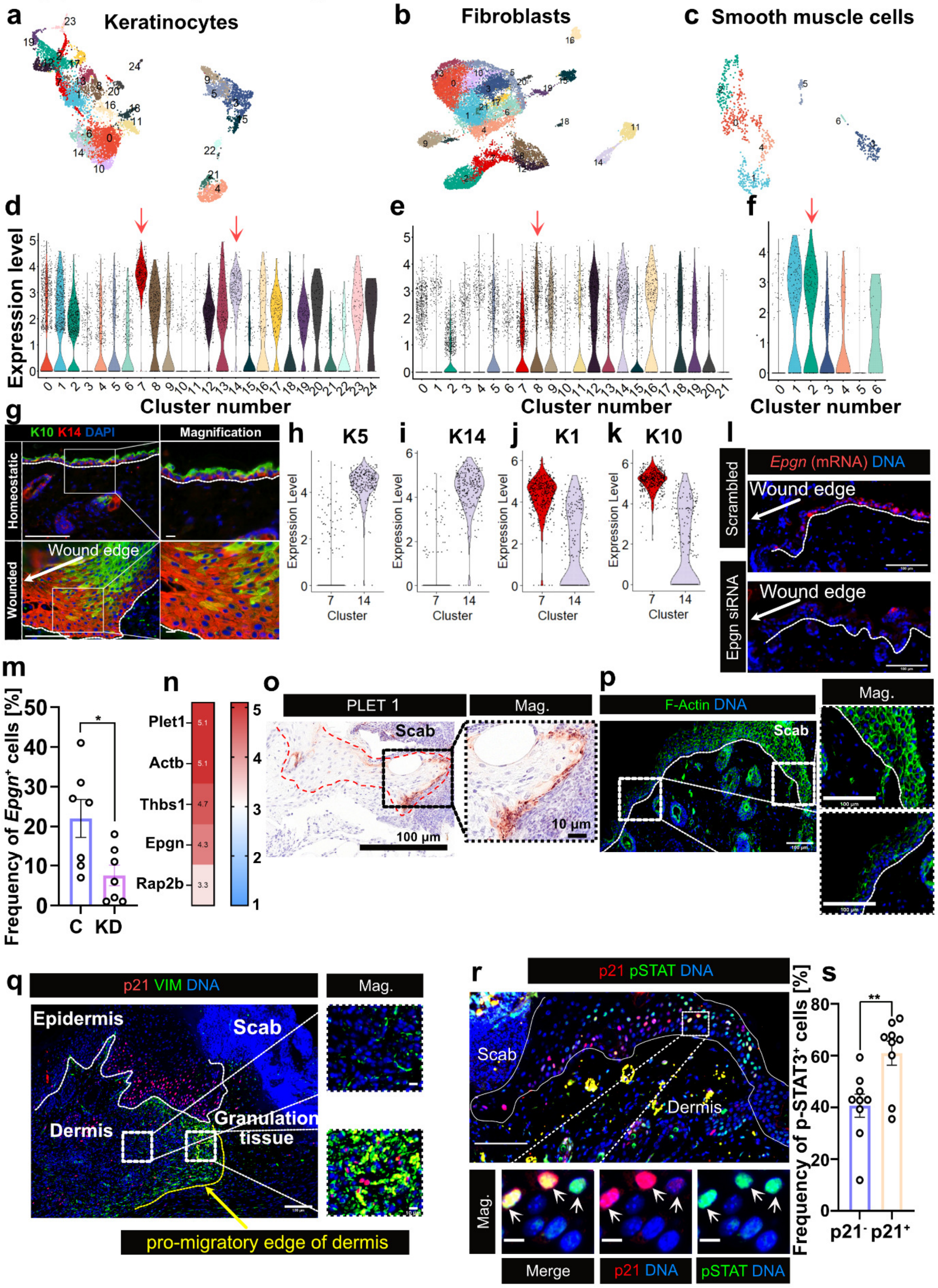

Supplementary Data Figure 1. *Cdkn1a* is associated with inflammation and migration. Clustering of mouse skin cells: **(a)** keratinocytes, **(b)** fibroblasts and **(c)** smooth muscle cells. Violin plots show *Cdkn1a* expression levels in clusters of: **(d)** keratinocytes, **(e)** fibroblasts and **(f)** smooth muscle cells clusters. Clusters of the highest *Cdkn1a* expression are marked with red arrows. **(g)** Representative images of control (homeostatic) mouse skin and skin proximal to the site of the wound showing distribution of keratins: keratin 10 (K10, green) and keratin 14 (K14, red). The regions marked with white lines in left panels are shown as magnifications on the right. The wound is on the left side and the scab is denoted in white letters.

Violin plots showing expression levels of **(h)** keratin 5 (K5), **(i)** K14, **(j)**, K1 and **(k)** K10 in keratinocyte clusters 7 and 14.

(l) Representative images of mouse skin collected from mice treated with siRNA against *Epgn* or with scrambled siRNA and stained using RNA-ISH for *Egpn* mRNA.

(m) Quantification of the effects of *Epgn* silencing on the expression of *Epgn* gene. P value is 0,0207.

(n) A heatmap showing the top upregulated genes related to migration of the cluster 14 and their consecutive fold-change increase over other clusters of keratinocytes.

(o) A representative image of IHC staining against Placenta Expressed Transcript 1 (PLET1). Red dashed line shows epidermal tongue and the black dashed lines mark the region magnified in the micrograph on the right side. The wound is on the right side and the scab is denoted in black letters. This experiment was performed once.

(p) A representative image of staining against F-Actin (green) in injured mouse skin. This experiment was performed once.

(q) A representative image of staining against vimentin (VIM; green) and p21 (red) in injured mouse skin. For both (p) and (q) magnifications on the right side represent homeostatic and

wounded sides of the skin with wound present on the right side. This experiment was performed once.

(r) A representative image of p-STAT3 (green) and p21 (red) staining of murine injured skin 3 days after wounding. White arrows mark $p21^+$ keratinocytes.

(s) Quantification of the frequency of keratinocytes positive for p-STAT3 and p21. P value is 0,0061.

In all the images, DNA is stained with DAPI or hematoxylin (blue). Data are from n = 7 mice per group for (m) and n = 8-9 for (s). Mean ± SEM plotted. An unpaired t-test (two-sided) was used for all the graphs. * is $p<0.05$ and ** is $p<0.01$. Scale bars for (g) show 100 and 10 µm for low and high magnification images respectively. Scale bars for (m), (o) and (r) are 100 µm (for the low magnification image) and 10 µm (magnifications of wounded and homeostatic sides). Scale bars for (l) and (n) are 100 µm. Source numerical data are available in source data.

Supplementary Data Figure 2

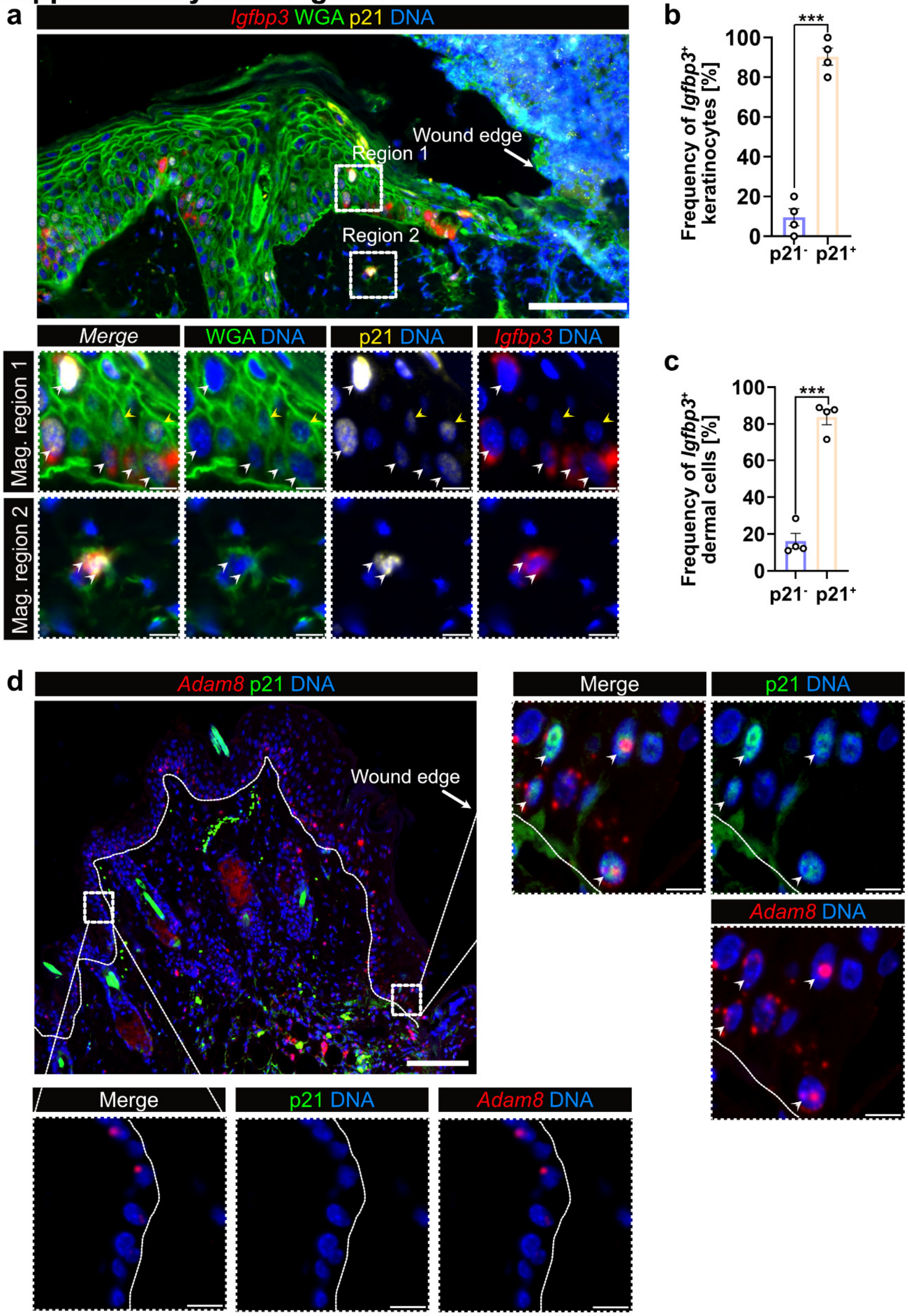

Extended Data Figure 2. Other SASP factors associated with p21 and injuries.

(**a**) Representative images of Immuno-RNA-ISH staining against *Igfbp3* mRNA (red), p21 protein (yellow) and WGA (green) in wounded murine skin 3 days after wounding.

Quantification of the frequency of *Igfbp3*$^{+}$ (**b**) keratinocytes (P value is <0,0001) and (**c**) dermal cells (P value is <0,0001) that are p21^{+} or p21^{-}.

(**d**) Representative images of Immuno-RNA-ISH staining against *Adam8* mRNA (red) and p21 protein (green) in wounded murine skin 3 days after wounding.

In all the images DNA is stained with DAPI (blue). Dashed line in (b) marks the basal lamina of epidermis. Data are from n = 4 mice per group for all the graphs. Mean ± SEM plotted. For all the graphs an unpaired t test (two-sided) was used. ***p<0.001. Scalebars for all images show 100 and 10 µm for low and high-magnification images respectively. Source numerical data are available in source data.

Supplementary Data Figure 3

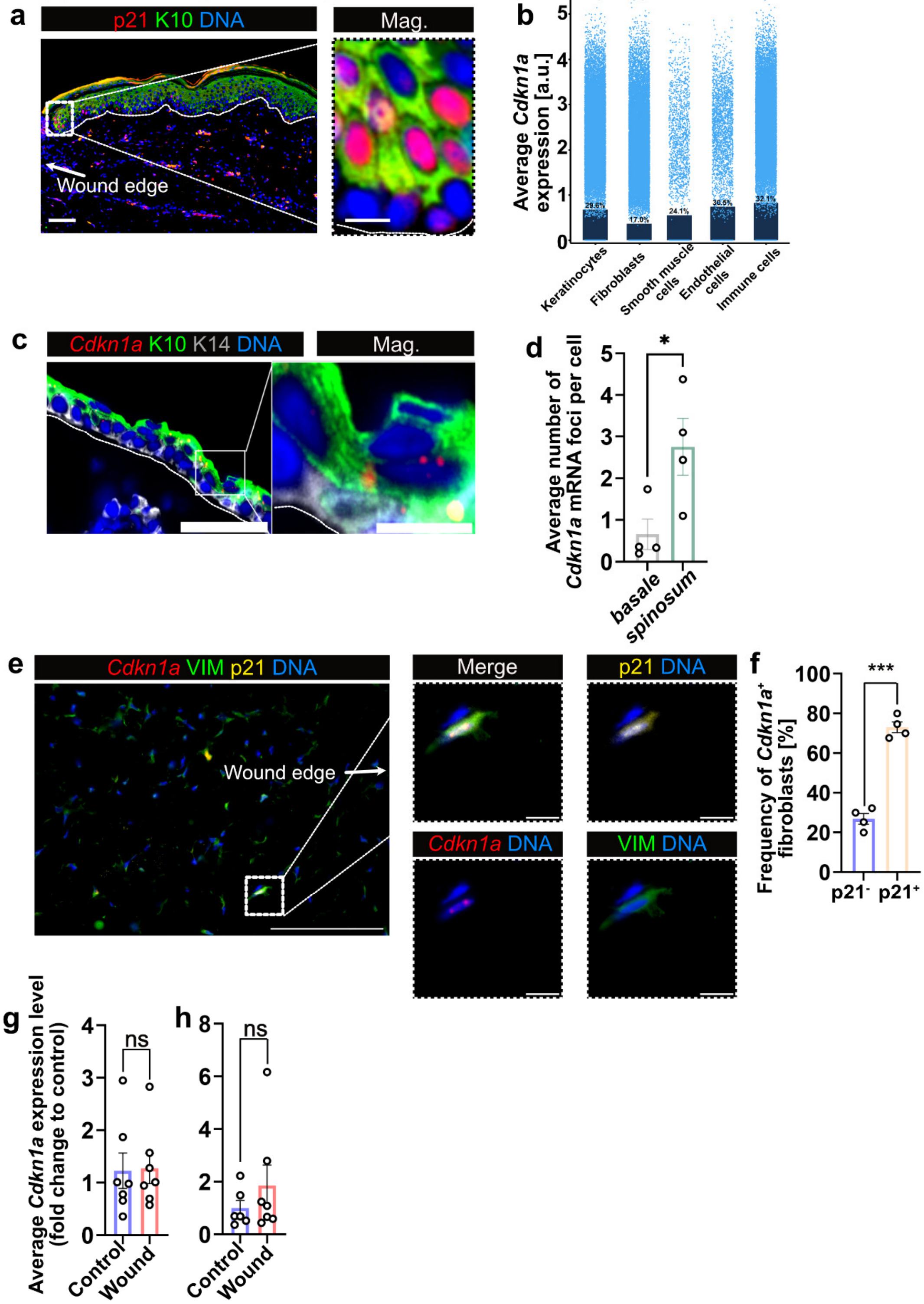

Supplementary Data Figure 3. Rapid-onset p21 induction is driven by the existing pool of *Cdkn1a* transcript and is transcription-independent.

(**a**) A representative image of porcine skin 3 h after wounding stained against p21 (red) and a marker of *stratum spinosum*, keratin 10 (K10, green). The wound is on the left side. The magnification on the right side shows the region marked with the white dashed lines (rotated).

(**b**) Average expression levels of *Cdkn1a* various types of cells from unwounded murine skin. Each dot represents an individual cell and an average is shown as a bar-graph. Values above each bar show the % of population positive the presence of *Cdkn1a* transcript.

(**c**) Representative images of RNA *in situ* hybridization (RNA-ISH) against *Cdkn1a* mRNA (red foci) co-stained with a marker of *stratum basale*, keratin 14 (K14; grey) and a marker of *stratum spinosum* keratin 10 (K10; green).

(**d**) Quantification of *Cdkn1a* mRNA foci in *stratum basale* ($K14^+$, $K10^-$) and *stratum spinosum* ($K14^-$, $K10^+$) keratinocytes. P value is 0,0351.

(**e**) A representative image of an Immuno-RNA-ISH staining against *Cdkn1a* (red), Vimentin (Vim; green) and p21 (yellow) of a sample from porcine injury 1.5 h after wounding.

(**f**) Quantification of the frequency of $Cdkn1a^+$ fibroblasts that are $p21^+$ or $p21^-$. P value is <0,0001.

Expression level of *Cdkn1a* as assessed by RT-qPCR in samples from mouse wounds collected (**g**) 3 h and (**h**) 6 h after the induction of an excision injury.

In all the images DNA is stained with DAPI (blue) and epidermis is outlined in white dashed lines. Data are from n = 4 mice per group for (d), n = 4 pigs for (f) and n = 7 mice per group for (g) and (h). Mean ± SEM plotted. For all the graphs an unpaired t test (two-sided) was used. ***p<0.001, *p<0.05 and "ns" stands for "non-significant". Scale bars for (a) and (e) show 100 and 10 μm and for (c) these are 50 and 10 μm for low and high magnification images respectively. Source numerical data are available in source data.

Supplementary Data Figure 4

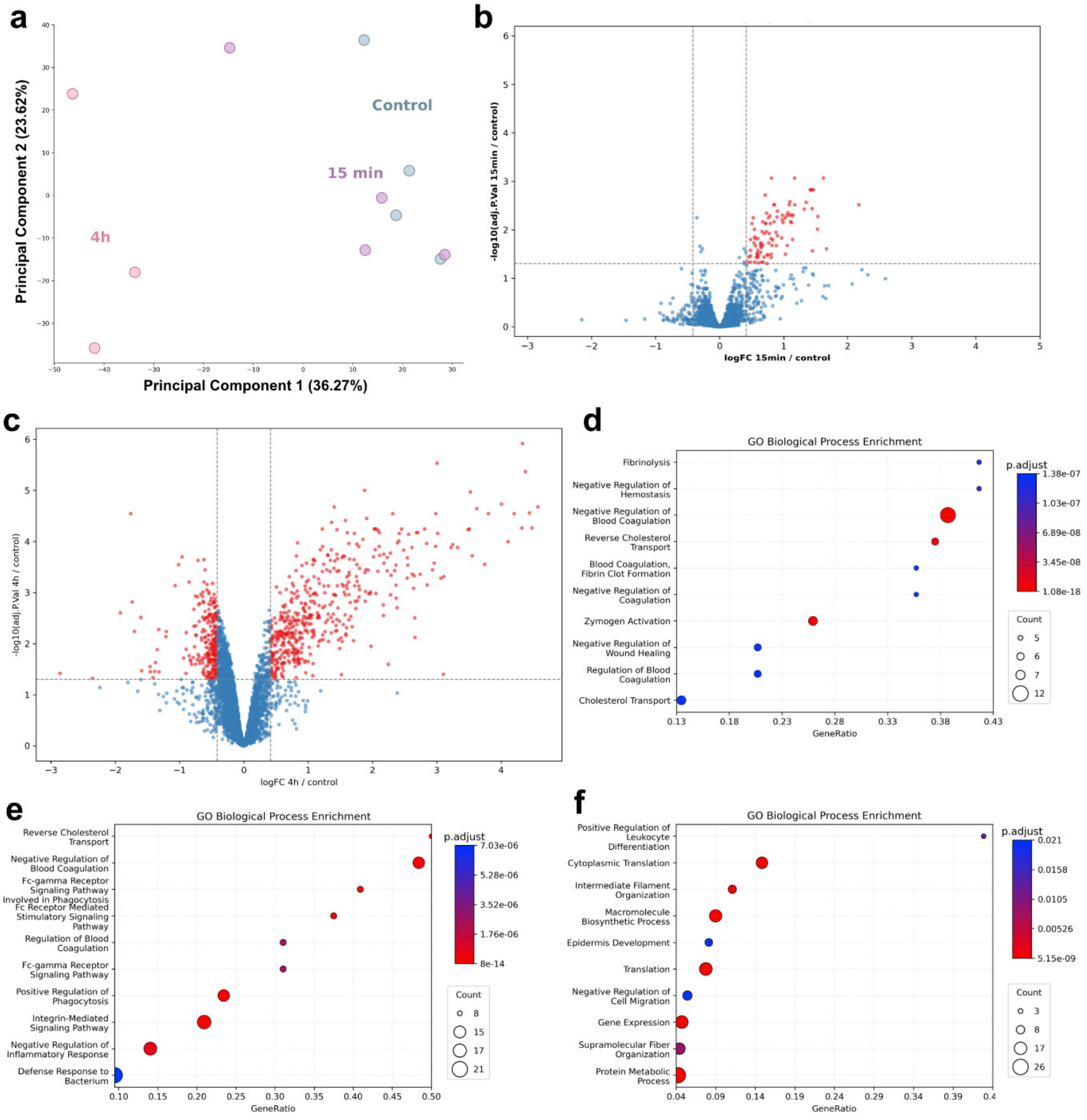

Supplementary Data Figure 4. Bulk proteomics of porcine excision injury at 15 minute and 4 hours post wounding.

(**a**) Principal component analysis (PCA) of proteomics data from porcine skin samples that were either untreated (Control; n = 4) or collected after excisional wounding at 15 minutes (“15 min”; n = 4) or 4 hours (“4 h”; n = 3).

(**b–c**) Volcano plots showing protein abundance changes in wound samples collected at (**b**) 15 minutes and (**c**) 4 hours post-injury. Red dots represent significantly (adjusted p value < 0.05

and FC > 1.333 or < 1/1.333) different proteins, while blue dots indicate non-significant changes.

(**d–f**) Dot plots showing Gene Ontology (GO)–enriched Kyoto Encyclopedia of Genes and Genomes (KEGG) Biological Process terms for differentially expressed genes (DEGs): (**d**) upregulated at 15 minutes post-wounding, (**e**) upregulated at 4 hours post-wounding, and (**f**) downregulated at 4 hours post-wounding.

Volcano plots (b, c) were generated from per-condition log2 ratios with Benjamini–Hochberg–adjusted p-values; significance was defined as adjusted p-value < 0.05 with effect-size thresholds FC > 1.333 or FC < 1/1.333. The bubble plots (d-f) were generated using enrichment analysis with Enrichr via gseapy, a Python package. The analysis employed Fisher's exact test for over-representation, using a one-sided (right-tailed) test to assess enrichment relative to the background. Multiple-testing correction was applied using the Benjamini–Hochberg (BH) FDR method across terms within each library.

Supplementary Data Figure 5

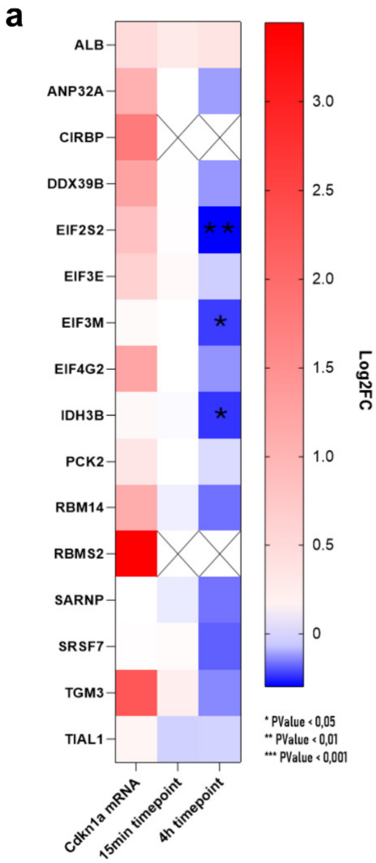

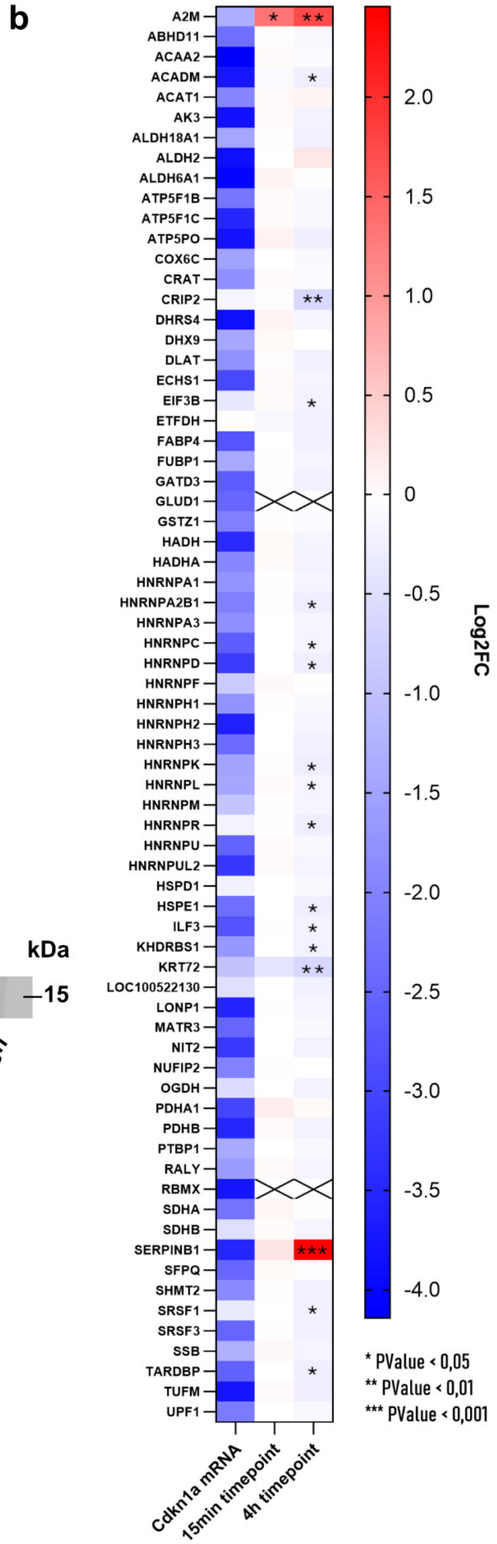

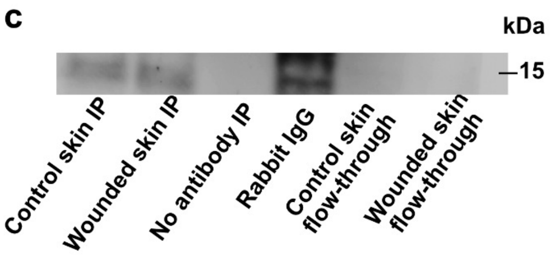

Supplementary Data Figure 5. Comparison of protein levels detected in the *Cdkn1a* mRNA pulldown and bulk proteomics of porcine excision wounds and results of RIP-qPCR of SRSF3 and *Cdkn1a*.

(**a–b**) Heatmaps showing log2 fold changes (log2FC) from the proteomic analysis of the *Cdkn1a* mRNA pulldown (left column) and bulk proteomics at 15 minutes (middle column) and 4 hours (right column) post-excision injury in porcine skin. Heatmaps display proteins (**a**) upregulated or (**b**) downregulated in association with *Cdkn1a* mRNA in porcine excision wounds and microneedling experiments.

(**c**) Western blot showing validation of SRSF3 RNA immunoprecipitation in wounded and control porcine skin, including anti-SRSF3, No-antibody, and Rabbit IgG controls, as well as flow-through fractions demonstrating SRSF3 depletion. This experiment was performed once. * is $p<0.05$, ** is $p<0.01$ and *** is $p<0.001$. Proteins not found in the (bulk) proteomics analyses are crossed out. Source unprocessed blots are available in source data.

SUPPLEMENTARY DATA TABLE LEGENDS

Supplementary Table 1. List of genes present in the cluster of p21^{high} keratinocytes. P values are calculated using Seurat default Wilcoxon rank-sum test (two-sided) with adjusted p values obtained from Benjamini–Hochberg multiple-testing correction.

Supplementary Table 2. List of genes present in the cluster of p21^{high} fibroblasts. P values are calculated using Seurat default Wilcoxon rank-sum test (two-sided) with adjusted p values obtained from Benjamini–Hochberg multiple-testing correction. p-values that are below 2.225074e-308 are marked as "0".

Supplementary Table 3. List of genes present in the cluster of p21^{high} smooth muscle cells. P values are calculated using Seurat default Wilcoxon rank-sum test (two-sided) with adjusted p values obtained from Benjamini–Hochberg multiple-testing correction.

Supplementary Table 4. Proteomics results of *Cdkn1a* pull-down

Supplementary Table 5. Sequences of raPOOL primers for pull-down of *Cdkn1a* mRNA and LacZ.

Supplementary Table 6. Results of the (bulk) proteomics analysis of the porcine excision injuries. Data processing was performed in R. After filtering out contaminants and proteins with missing quantification values, the LIMMA package was used to perform the statistical analysis (P-value, fold change, two-sided multiple testing-correction) with batch correction for each pig.

Supplementary Table 7. The light microscopy reporting table.
