# Supplementary material for: Transcription-independent induction of rapid-onset senescence is integral to healing
Source: Nat Cell Biol. 2026 May 28;28(6):1281–99. doi: 10.1038/s41556-026-01948-2 (PMC13279267; doi:10.1038/s41556-026-01948-2)
Supplement: Supplementary file 11 — Unprocessed western blots and/or gels. [file 41556_2026_1948_MOESM11_ESM.pdf]

Fig. 5i

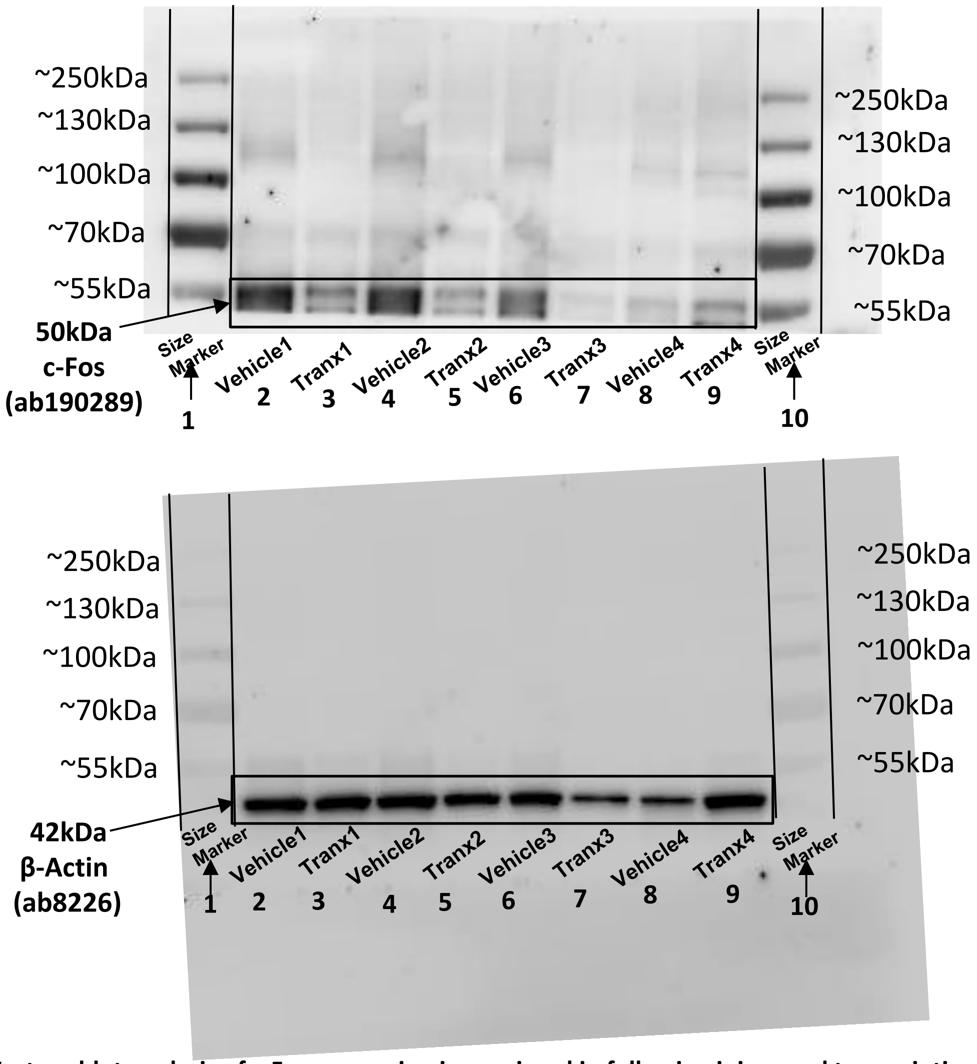

Original Western blot analysis of c-Fos expression in porcine skin following injury and transcriptional inhibition.
Original Western blot (WB) images showing c-Fos and β-Actin protein levels in porcine skin samples collected 1.5 h after injury and subsequent treatment with vehicle or transcription inhibitors (Tranx). The same protein samples were loaded onto two separate membranes owing to the similar molecular weights (kDa) of c-Fos and β-Actin to avoid overlapping signals. For the c-Fos blot, the membrane was cut after transfer; the remaining portion of the same blot was used for the detection of additional proteins not included in this experiment. The positions of the molecular weight markers (kDa) are indicated on the left (position 1) and right (position 10) side of the blots. Molecular weight estimation was performed using a prestained protein ladder (PageRuler™ Prestained Protein Ladder Plus, Catalog No. PI26619, Thermo Scientific), containing nine proteins spanning a size range of 10–250 kDa. These original Western blots correspond to the cropped panels shown in Fig. 5i. Full-length, original blot images are shown.

Fig. 5k

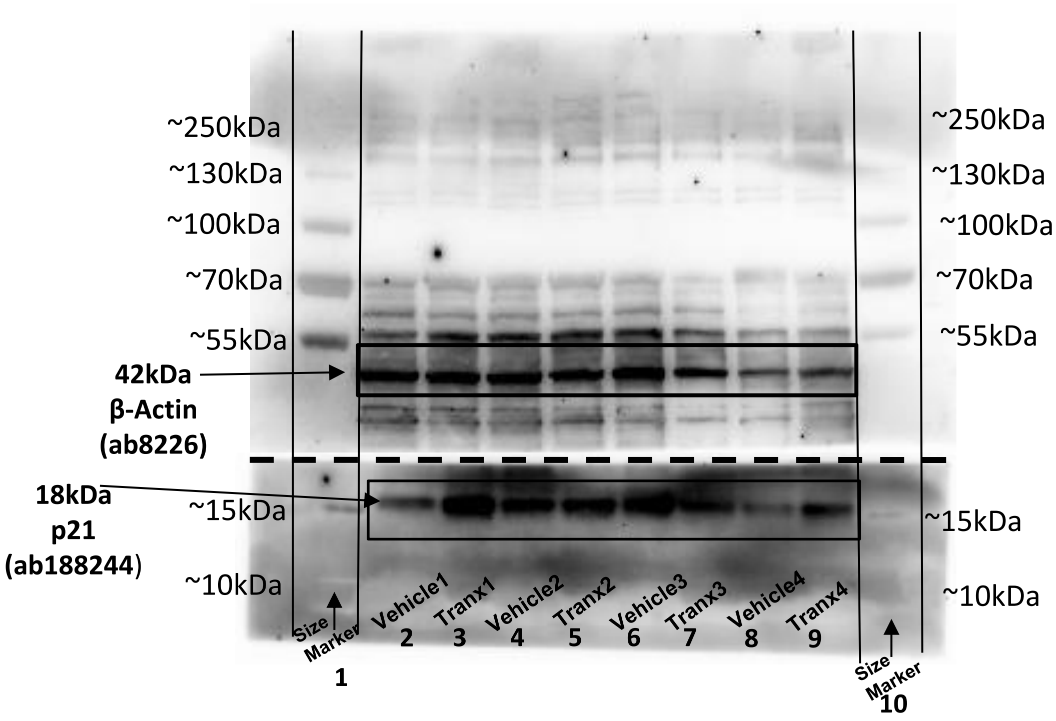

Original Western blot analysis of p21 in porcine skin following injury and Tranx treatment. Original Western blot (WB) images showing p21 and β-Actin protein levels in porcine skin samples collected 1.5 h after injury and subsequent treatment with vehicle or Tranx. The positions of the molecular weight markers (kDa) are indicated on the left (position 1) and right (position 10) side of the blots. Molecular weight estimation was performed using a prestained protein ladder (PageRuler™ Prestained Protein Ladder Plus, Catalog No. PI26619, Thermo Scientific), containing nine proteins spanning a size range of 10–250 kDa. Blots were cropped and assembled for clarity; the dashed line indicates regions where the original membranes were cut due to different exposure times during signal acquisition. These original Western blots correspond to the cropped panels shown in Fig. 5k. Full-length, original blot images are shown.

Fig. 6c

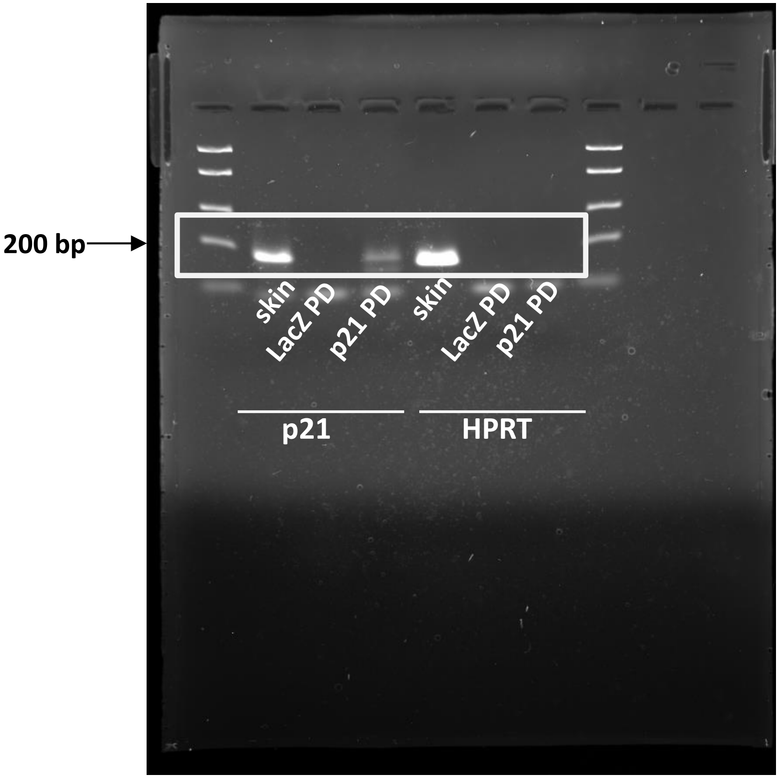

Original agarose gel electrophoresis images of PD products amplified by RT–qPCR were used to assess Cdkn1a cDNA enrichment in porcine skin samples. Analyses were performed on positive control samples (wounded porcine skin prior to PD) and on samples obtained following Cdkn1a and LacZ mRNA pull-down. Amplification of HPRT served as a negative control to confirm pull-down specificity. Full-length, uncropped gel images are shown. These original agarose gel correspond to the cropped panels shown in Fig. 6c.

Extended Data Fig. 4k.

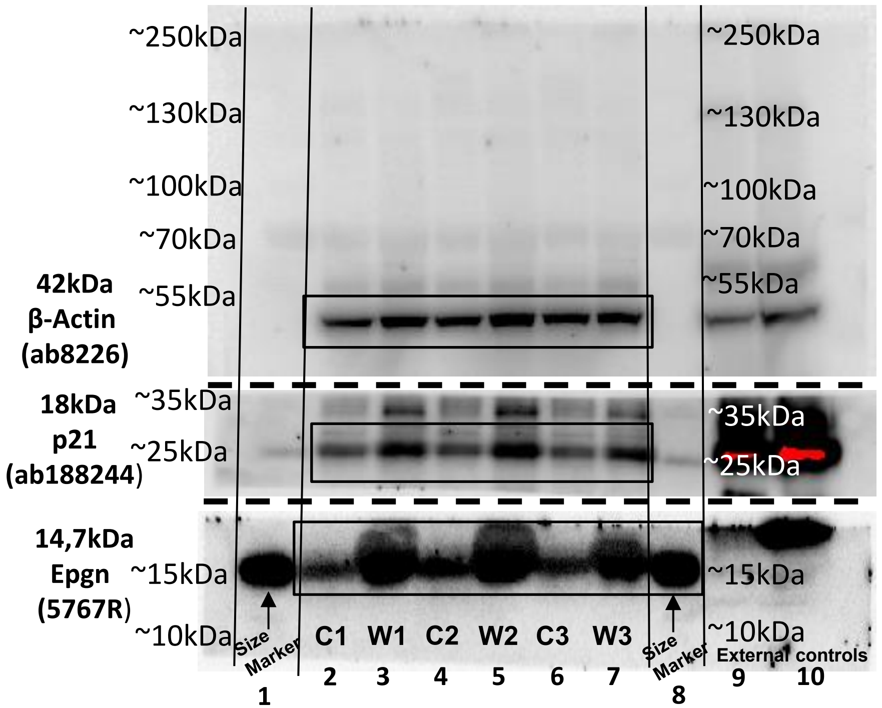

Ponceau S staining

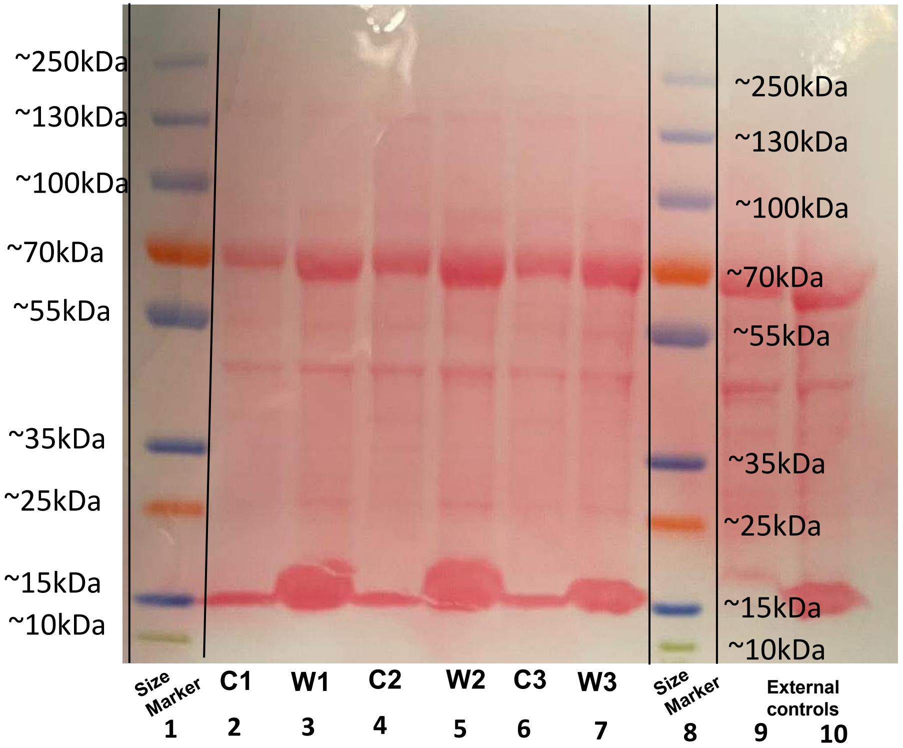

Original Western blot data corresponding to Extended Data Fig. 4k.
Original Western blot (WB) images showing p21, EPGN and β-Actin protein levels in control (C) and wound (W) samples of porcine skin collected 3 h after injury. External controls (position 9,10) are indicated. The positions of the molecular weight markers (kDa) are indicated on the left (position 1) and right (position 8) side of the blots. The positions of the molecular weight markers (kDa) are indicated on the left (position 1) and right (position 8) side of the blots. Because the molecular weight marker signal was faint in the chemiluminescent image, the marker lane visualized by Ponceau S staining after transfer is shown on the left to indicate the positions of molecular weight standards. Molecular weight estimation was performed using a prestained protein ladder (PageRuler™ Prestained Protein Ladder Plus, Catalog No. PI26619, Thermo Scientific), containing nine proteins spanning a size range of 10–250 kDa. Blots were cropped and assembled for clarity; dashed lines indicate regions where the original membranes were cut due to different exposure times during signal acquisition. These original Western blots correspond to the cropped panels shown in Extended Data Fig. 6k. Full-length, original blot images are shown.

Supplementary Data Fig. 5c

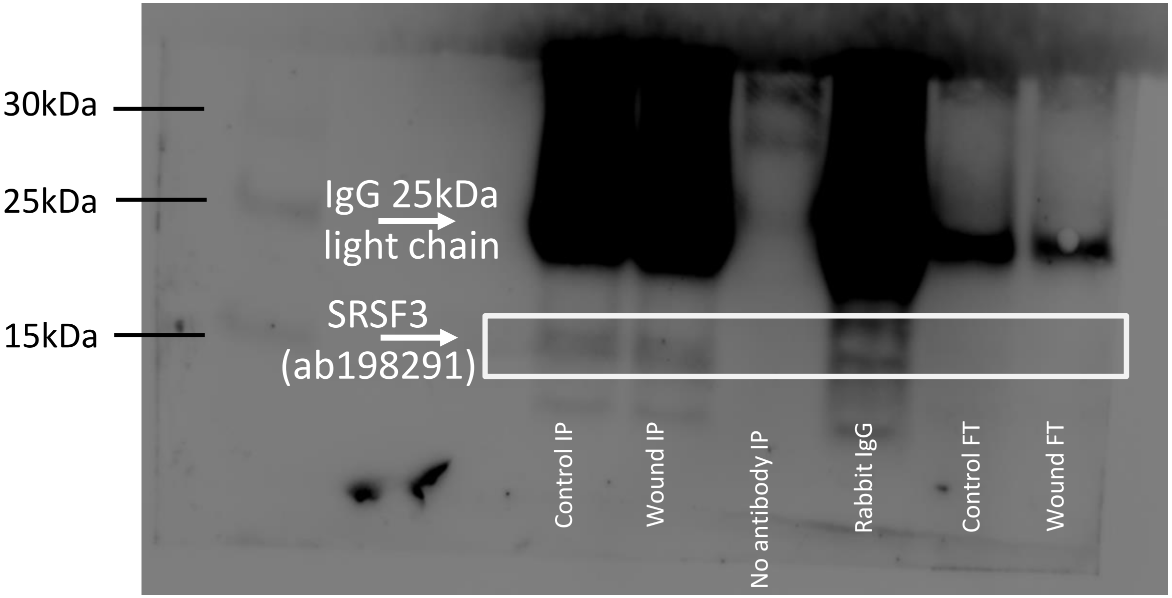

Original Western blot for the validation of SRSF3 RNA immunoprecipitation by immunoblotting of porcine skin following injury (wound IP) and unwounded, control skin (Control IP).
Original Western blot (WB) images showing 5% of the Protein A bead fraction from each RIP condition. Samples included Control + anti-SRSF3, Wound + anti-SRSF3, and the corresponding No-antibody (No-Ab) control processed in parallel. In addition, a Rabbit IgG control was included on the blot to exclude nonspecific bands. The flow-through (FT) fractions collected after bead incubation were analysed for Control and Wound lysates to document depletion of SRSF3 from the supernatant upon immunoprecipitation. The complete, uncropped blot is shown with molecular weight markers and the region corresponding to SRSF3 indicated.

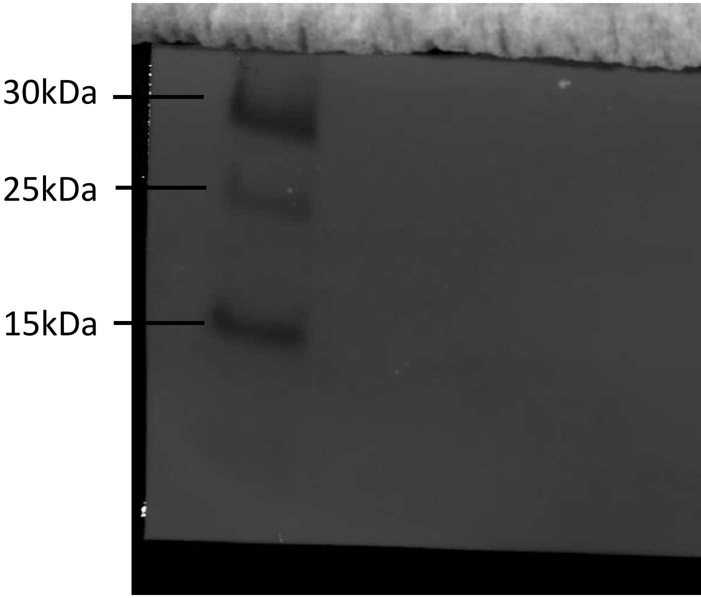

Colorimetric acquisition for original Western blot for Supplementary Data Fig 5c.
Original Western blot (WB) image, colorimetric, depicting the protein ladder. Visible bands are annotated.
