# Supplementary material for: Transcription-independent induction of rapid-onset senescence is integral to healing
Source: Nat Cell Biol. 2026 May 28;28(6):1281–99. doi: 10.1038/s41556-026-01948-2 (PMC13279267; doi:10.1038/s41556-026-01948-2)
Supplement: Supplementary file 3 — Peer Review file [file 41556_2026_1948_MOESM3_ESM.pdf]

nature portfolio

Peer Review File

Transcription-independent induction of rapid-onset senescence is integral to healing

Corresponding Author: Dr Mikolaj Ogrodnik

Version 0:

Decision Letter:

Dear Dr Ogrodnik,

Your manuscript "Transcription-independent induction of rapid-onset senescence is integral to healing", has now been seen by 4 referees, who are experts in senescence (referee 1); senescence (referee 2); senescence (referee 3); and stem cells, wound healing/regeneration (referee 4), and whose comments are pasted below. In light of their advice, we regret that we cannot offer to publish the study in Nature Cell Biology.

Nature and Nature Cell Biology are editorially independent. However, in cases such as this, when authors transfer a manuscript to Nature Cell Biology after it has been reviewed for Nature, Nature passes the referees' comments (and their identities) to us, with the author's permission. Please note that all 4 original referees have assessed the revision and retain the same numbers.

As you will see, although reviewers 1 and 3 offer positive comments, reviewers 2 and 4 continue to have persisting concerns with regard to the mechanism of p21 sequestration and rapid translation upon wound healing and the connection between mTOR and p21, and we consider these reservations sufficiently important to preclude publication of the study in Nature Cell Biology.

Please contact me if you would like me to consult my colleagues at Nature Communications regarding moving forward with your manuscript.

Although we cannot offer to publish your manuscript, I suggest that you consider Nature Communications as a suitable venue for this work. To transfer your manuscript, please use our manuscript transfer portal. You will not have to re-supply manuscript metadata and files, unless you wish to make modifications. For more information, please see our manuscript transfer FAQ page.

We are very sorry that we could not be more positive on this occasion, but we thank you for the opportunity to consider this work.

With kind regards,
Angela Parrish

Angela R Parrish, PhD
Locum Senior Editor
Nature Cell Biology

Reviewers' comments:

Reviewer #1 (Remarks to the Author):

The authors have addressed all of my concerns and the manuscript is greatly improved. Congratulations.

Reviewer #2 (Remarks to the Author):

I commend the intensive efforts of the authors in expanding the manuscript, particularly in clarifying the p21-lineage tracing

system, improving figure consistency, and integrating additional molecular data. Despite these enhancements, I remain unconvinced that the revision sufficiently addresses the core mechanistic questions. Moreover, recent literature raises notable contradictions. Gasek et al have recently demonstrated that clearing p21-high senescent cells actually expedites wound closure, mediated at least in part by NF-κB inhibition. This finding directly opposes the primary assertion of the current manuscript, which posits that $p21^{+}$ senescent cells are essential for wound healing. Unfortunately the authors merely cite and mention this recent manuscript without actually offering any potential explanation for the opposite results. Major points of concern:

1. Although additional markers have been provided, the work still lacks functional validation of true, irreversible senescence. The lineage tracking shows persistence, but may equally reflect reporter stability. It remains a stretch to define thi cell population as fully senescent.

2. Despite added experiments, the link between basal p21 mRNA levels and rapid protein induction remains inferential. Transcription-blocking agents are not ideal controls; translation inhibition suppresses all protein synthesis, not just p21. The RNA-protein binding data remain correlative and descriptive. More direct manipulation is needed to support a causal model.

3. The pulse-chase tracing with tdTomato lacks power and fails to rigorously prove that the same keratinocytes harboring high baseline p21 mRNA are tracked and eliminated. The rare occurrence of $tdTom^{+}/p21^{-}$ clusters undermines fidelity of the system. The possibility of reporter persistence or artifact remains unaddressed.

4. While the authors suggest that mTOR inhibition reduces p21 induction, this remains superficial pharmacology without mechanistic clarity. The notion of transcription-independent initiation followed by transcription-dependent reinforcement is interesting but not empirically distinguished. The actual signaling triggers such as mechanosensing, osmolarity, or others are purely speculative.

Excluding results like IL-6 (reported as “irregular and scattered”) without robust validation or explanation raises concerns about selective reporting. Both positive and negative findings need transparent inclusion to avoid bias.

Reviewer #2 (Remarks on Protocol(s)):

they seem accurate and sufficiently detailed

Reviewer #3 (Remarks to the Author):

The authors have made a comprehensive work in revising the manuscript. While they might not have addressed each and any of the sggestions of all the reviewers, the manuscript has improved in this revision and I am satisfied

Reviewer #3 (Remarks on Protocol(s)):

Yes

Reviewer #4 (Remarks to the Author):

While the authors had tried (often unsuccessfully) to address the different questions raised during the initial review, the conceptual novelty of this manuscript remain extremely limited. If we replaced the word senescence by terminal differentiation, most of the data have been previously described.

Unfortunately, the mechanistic insights about the sequestration and rapid translation of p21 upon wounding had not been significantly improved.

As it stands, this manuscript would be better suitable for a more specialized journal such as Nat Communications.

**For Nature Portfolio general information and news for authors, see http://npg.nature.com/authors.

Version 1:

Decision Letter:

*Please delete the link to your author homepage if you wish to forward this email to co-authors.

Dear Dr Ogrodnik,

Thank you for your patience while we requested further comment from an arbitrator and discussed their comments within the team.

Your manuscript, "Transcription-independent induction of rapid-onset senescence is integral to healing", has now been seen by 1 arbitrating referee, who is an expert in senescence (referee 1). We are willing to allow one more round of revision to address the remaining concerns. Please be aware that further reconsideration of this manuscript (in the event of re-review) will be conditional on the referee being fully satisfied with the extent of the revisions.

Nature Cell Biology editors discuss the referee reports in detail within the editorial team, including the chief editor, to identify key referee points that should be addressed with priority, and requests that are overruled as being beyond the scope of the current study. To guide the scope of the revisions, I have listed these points below. We are committed to providing a fair and constructive peer-review process, so please feel free to contact me if you would like to discuss any of the referee comments further.

In particular, it would be essential to:

+ address point 5 regarding p53 levels and p21 kinetics

+ address point 6 regarding specificity of p21 for SRSF

+ All other referee concerns pertaining to strengthening existing data, providing controls, methodological details, clarifications and textual changes, should also be addressed.

+ Finally please pay close attention to our guidelines on statistical and methodological reporting (listed below) as failure to do so may delay the reconsideration of the revised manuscript. In particular please provide:

- a Supplementary Figure including unprocessed images of all gels/blots in the form of a multi-page pdf file. Please ensure that blots/gels are labeled and the sections presented in the figures are clearly indicated.

- a Supplementary Table including all numerical source data in Excel format, with data for different figures provided as different sheets within a single Excel file. The file should include source data giving rise to graphical representations and statistical descriptions in the paper and for all instances where the figures present representative experiments of multiple independent repeats, the source data of all repeats should be provided.

We would be happy to consider a revised manuscript that would satisfactorily address these points, unless a similar paper is published elsewhere, or is accepted for publication in Nature Cell Biology in the meantime.

When revising the manuscript please:

- ensure that it conforms to our format instructions and publication policies (see below and https://www.nature.com/nature/for-authors).

- provide a point-by-point rebuttal to the full referee reports verbatim, as provided at the end of this letter.

- for any revision that includes light microscopy data, we ask our authors to please include a completed light microscopy reporting table https://www.nature.com/documents/Light_microscopy_reporting_table.xlsx to ensure the methods are described thoroughly. The table will be available to reviewers and ultimately published should the manuscript be accepted at the journal.

- provide the completed Reporting Summary (found here https://www.nature.com/documents/nr-reporting-summary.pdf). This is essential for reconsideration of the manuscript will be available to editors and referees in the event of peer review. For more information see http://www.nature.com/authors/policies/availability.html or contact me.

When submitting the revised version of your manuscript, please pay close attention to our href="https://www.nature.com/nature-portfolio/editorial-policies/image-integrity">Digital Image Integrity Guidelines. and to the following points below:

-- that unprocessed scans are clearly labelled and match the gels and western blots presented in figures.
-- that control panels for gels and western blots are appropriately described as loading on sample processing controls
-- all images in the paper are checked for duplication of panels and for splicing of gel lanes.

Finally, please ensure that you retain unprocessed data and metadata files after publication, ideally archiving data in perpetuity, as these may be requested during the peer review and production process or after publication if any issues arise.

Nature Cell Biology is committed to improving transparency in authorship. As part of our efforts in this direction, we are now requesting that all authors identified as ‘corresponding author’ on published papers create and link their Open Researcher and Contributor Identifier (ORCID) with their account on the Manuscript Tracking System (MTS), prior to acceptance. ORCID helps the scientific community achieve unambiguous attribution of all scholarly contributions. You can create and link your ORCID from the home page of the MTS by clicking on ‘Modify my Springer Nature account’. For more information please visit please visit www.springernature.com/orcid.

This journal strongly supports public availability of data. Please place the data used in your paper into a public data repository, or alternatively, present the data as Supplementary Information. If data can only be shared on request, please explain why in your Data Availability Statement, and also in the correspondence with your editor. Please note that for some data types, deposition in a public repository is mandatory - more information on our data deposition policies and available repositories appears below.

Please submit the revised manuscript files and the point-by-point rebuttal to the referee comments using this link:

Link Redacted

*This url links to your confidential home page and associated information about manuscripts you may have submitted or be reviewing for us. If you wish to forward this email to co-authors, please delete the link to your homepage.

We would like to receive a revised submission within 4-8 weeks.

We hope that you will find our referees' comments, and editorial guidance helpful. Please do not hesitate to contact me if there is anything you would like to discuss.

Best wishes,

Angela Parrish

Angela R Parrish, PhD
Locum Senior Editor
Nature Cell Biology

Reviewers' Comments:

Reviewer #5 (Remarks to the Author):

Valdivieso K,... …Ogrodnik M, Transcription-independent induction of rapid-onset senescence is integral to healing
Submitted to Nature Cell Biology

This is an additional comment on an ongoing controversial debate between two of the four consulted referees (#2 and #4) and the authors. As a late-entering expert #5, I provide here focused comments on this controversy and my overall impression of the manuscript – with the caveat that I had no access to the Supplementary Information/Extended Data Files.

The central observation of this work is the unexpectedly early detectability of high-level p21 protein-expressing keratinocytes just hours after skin injury, interpreted as rapid-onset senescence during wound healing by the authors. The key claim is the transcription-independent induction of p21 protein expression via immediate translation of pre-elevated p21 transcripts in the nucleus.

With respect to referee #2:
- Clashing evidence recently published in a different paper (Gasek et al., Nat Aging, 2024): This should be appropriately discussed here
- Lacks functional validation of true, irreversible senescence: I do not see this as a fundamental concern; please find my points on other types of cellular senescence below.
- Evidence on basal p21 mRNA levels and rapid protein induction: I partly agree, see my points below
- Technical issues with tdTomato-governed pulse-chase tracing: I partly agree, see my points below
- Impact of mTOR inhibition on p21 induction: I partly agree, see my points below
- Excluding results like IL-6: I cannot comment, this did not come up to my attention

With respect to referee #4:
- Conceptual novelty of this manuscript remains extremely limited: I disagree; I find the primed-for-senescence concept with pre-elevated p21 transcripts appealing and novel
- Mechanistic insights about sequestration and rapid translation of p21 upon wounding: I cannot comment on improvements during review, but see this topic addressed, albeit with some concerns, as detailed below

In essence, extensive time and resources have been invested so far to improve the manuscript based on previous statements by four referees. I share some but not all concerns of the two colleagues that remain reserved. However, my perception of the manuscript is that a thorough textual revision including better presentation of the figure material (where, how, and legends) may substantially strengthen this manuscript – which will advance the field, not without stimulating discussions and controversies, but adequate for the high level of NCB.

Major concerns and comments

1. It is intrinsic to the claim of "rapid-onset senescence" that not all features of a senescent cell (morphology, epigenomic remodeling, lysosomal changes, dynamics of the secretome over time etc.) will be found in cells that start to exhibit signs of commencing senescence unusually early. The authors should state this more carefully. Whether these early-senescent cells would potentially become truly and full-featured senescent at later times remains rather philosophical, since the fate of these keratinocytes is to be rapidly turned over by shedding anyway, irrespective of their senescent-or-not status. Of note, the authors initially also report detection of p21-high fibroblasts and myocytes, but do not follow up on those more wound-resident cells later. While p21 is essential for keratinocyte senescence (Missero et al., Genes Dev 1996), more information on paralleling or delayed kinetics of the other central cell-cycle-inhibiting senescence indicator/mediator p16INK4a would have been appreciated.

2. It would have helped to elaborate more on the underlying cell biologic, molecular, biomechanic and biochemical aspects of the injury set here to initiate the wound healing process. Excision and micro needle are the technicalities, but what is the actual damage inflicted? How exclusive is this procedure to the injured skin, as compared to the surgically wounded gut mucosa and wall during anastomosis healing, or liver damage after puncture etc.? How comparable is mechanic injury to UV light- or gamma-irradiation-mediated skin damage, or other types of drug-induced senescence by systemic (e.g. chemotherapeutics, or CCl4-mediated hepatotoxicity) or topically applied drugs (such as DMBA/TPA to induce Ras-senescent lesions)? I'm not asking for all those experiments, but would like to see the work presented in the wider scope of the senescence field. The discussion only superficially touches upon muscle and bone healing.

It seems that the skin comes with specific properties not applying to other damage-induced senescence scenarios – and this might be its pre-exposure to UV light, potentially explaining the pre-elevated p21 transcript expression as sort of a senescence-primed/-prone standby mode. Hence, experiments on long-term light-deprived skin or mice would have potentially been insightful. Likewise, the authors did not consider to test damage-accumulated old skin (leading to "actinic keratosis" in humans) vs. young skin.

3. Data on the secreted factor epigen appear rather isolated and non-central to the manuscript. Similarly, the Perilipin-related lipid droplets are a side story of limited value.

4. The CIP1 (p21) promoter-driven Cre:ER crossed to the Rosa26-LSL-dtTomato strain is, in principle, a powerful tool, but the information provided about timing of the tamoxifen (Tam) pulse-chase administration – e.g. "at the time of injury" – is not clear enough to allow readers to understand the biological consequences, i.e. how quick a p21 promoter-driven Cre:ER product would be available for Tam to unleash a red-fluorescent read-out, especially in light of the author-undervalued aspect that p21 transcripts (hence, the Cre:ER transcript as well?) are pre-elevated in skin keratinocytes…

5. The idea of stress-driven, pre-elevated p21 transcripts in the nucleus of light-exposed keratinocytes to prime for rapid-onset senescence is interesting. However, why these transcript levels do not further increase upon acutely inflicted tissue injury remains unexplained. The authors report higher p53 protein levels and elevated p21 transcript levels in the pre-injury normal skin, and argue that binding of the p21 transcript to certain RNA-binding proteins prevents its translation, ok. But what happens to p53 levels as the p21-driving transcription factor during injury, why is p53 protein found low in the wound? Why are no other wound-associated mechanisms – namely FoxO/TGF-beta-Smads – employed to drive p21 transcription (Seonae et al., Cell 2004; Ponugoti et al., JCB 2013)?

And, while there might be a small fraction of p21 protein expression to be controlled via mTOR-governed translation (and, hence, amenable to mTOR inhibition [Beuvink et al., Cell 2005]), other but transcription-dependent mechanisms might apply to p21 regulation upon mTOR inhibition: mTOR inhibition will reduce feedback inhibition of PI3K activity, allowing AKT to inactivate FoxOs, which, together with Smads (see above) drive p21 translation.

Moreover, the work is inconsistent to whether or not p21 transcripts are further induced upon injury (claimed stable, but found "amplified" in mRNA pull-down experiments).

6. How p21-specific the SRSF binding to mRNA really is would need to be shown; conversely, the reduced detectability of SRSF3 and hnRNP proteins by mRNA pull-down may be due to injury-related dissociation from the transcript, or by lowered protein expression for any other reason. The authors assume that this will enable the mRNA's export to the cytoplasm, but nuclear translation (Reid and Nicchitta, JCB 2012) of p21 as an alternative and very rapid stress-related alternative is not even discussed.

Minor concerns

1. The writing style requires revision for clarity. For instance, "…healing process. One step in this process is the formation of the p-rpS6-zone, which occurs within mere minutes after wounding…" refers to a previous finding of the last author, but remains without any explanation. What that zone is, how big it is, etc. is important for NCB's readership, and is certainly not common knowledge in the scientific community. As another example, "Mechanistically, Cdkn1a transcripts remove proteins, …" is scientifically simply wrong. Or the entire sub-paragraph starting with "In all our tracking experiments we observed a single instance of a cluster tdTom+ keratinocytes that was mostly negative… …demonstrate that injury-induced senescent cells are removed after wound closure via distinct, cell-type specific mechanisms" is not only impossible to follow at the outset, but presents a conclusion at the end for which the underlying data are missing.

2. Often (especially in the legends) the authors miss to clearly state whether they talk about protein or transcript expression – although this distinction is central. Moreover, sometimes, only data on wounded, not non-wounded material is shown, or cell numbers cannot be compared (e.g. p21+ cells per mm2 vs. frequency of p21+ cells).

3. Why is p21/CIP1/CDKN1A transcript expression, although central to the manuscript, only shown in supplementary figures?

GUIDELINES FOR MANUSCRIPT SUBMISSION TO NATURE CELL BIOLOGY

READABILITY OF MANUSCRIPTS – Nature Cell Biology is read by cell biologists from diverse backgrounds, many of whom are not native English speakers. Authors should aim to communicate their findings clearly, explaining technical jargon that might be unfamiliar to non-specialists, and avoiding non-standard abbreviations. Titles and abstracts should concisely communicate the main findings of the study, and the background, rationale, results and conclusions should be clearly explained in the manuscript in a manner accessible to a broad cell biology audience. Nature Cell Biology uses British spelling.

MANUSCRIPT FORMAT – please follow the guidelines listed in our Guide to Authors regarding manuscript formats at Nature Cell Biology.

TITLE – should be no more than 100 characters including spaces, without punctuation and avoiding technical terms, abbreviations, and active verbs..

AUTHOR NAMES – should be given in full.

AUTHOR AFFILIATIONS – should be denoted with numerical superscripts (not symbols) preceding the names. Full addresses should be included, with US states in full and providing zip/post codes. The corresponding author is denoted by: "Correspondence should be addressed to [initials]."

ABSTRACT AND MAIN TEXT – please follow the guidelines that are specific to the format of your manuscript, as listed in our Guide to Authors (http://www.nature.com/ncb/pdf/ncb_gta.pdf) Briefly, Nature Cell Biology Articles, Resources and Technical Reports have 3500 words, including a 150 word abstract, and the main text is subdivided in Introduction, Results, and Discussion sections. Nature Cell Biology Letters have up to 2500 words, including a 180 word introductory paragraph (abstract), and the text is not subdivided in sections.

ACKNOWLEDGEMENTS – should be kept brief. Professional titles and affiliations are unnecessary. Grant numbers can be listed.

AUTHOR CONTRIBUTIONS – must be included after the Acknowledgements, detailing the contributions of each author to the paper (e.g. experimental work, project planning, data analysis etc.). Each author should be listed by his/her initials.

FINANCIAL AND NON-FINANCIAL COMPETING INTERESTS – the authors must include one of three declarations: (1) that they have no financial and non-financial competing interests; (2) that they have financial and non-financial competing interests; or (3) that they decline to respond, after the Author Contributions section. This statement will be published with the article, and in cases where financial and non-financial competing interests are declared, these will be itemized in a web supplement to the article. For further details please see https://www.nature.com/licenceforms/nrg/competing-interests.pdf.

REFERENCES – are limited to a total of 70 for Articles, Resources, Technical Reports; and 40 for Letters. This includes references in the main text and Methods combined. References must be numbered sequentially as they appear in the main text, tables and figure legends and Methods and must follow the precise style of Nature Cell Biology references. References only cited in the Methods should be numbered consecutively following the last reference cited in the main text. References only associated with Supplementary Information (e.g. in supplementary legends) do not count toward the total reference limit and do not need to be cited in numerical continuity with references in the main text. Only published papers can be cited, and

each publication cited should be included in the numbered reference list, which should include the manuscript titles. Footnotes are not permitted.

METHODS – Nature Cell Biology publishes methods online. The methods section should be provided as a separate Word document, which will be copyedited and appended to the manuscript PDF, and incorporated within the HTML format of the paper.

Methods should be written concisely, but should contain all elements necessary to allow interpretation and replication of the results. As a guideline, Methods sections typically do not exceed 3,000 words. The Methods should be divided into subsections listing reagents and techniques. When citing previous methods, accurate references should be provided and any alterations should be noted. Information must be provided about: antibody dilutions, company names, catalogue numbers and clone numbers for monoclonal antibodies; sequences of RNAi and cDNA probes/primers or company names and catalogue numbers if reagents are commercial; cell line names, sources and information on cell line identity and authentication. Animal studies and experiments involving human subjects must be reported in detail, identifying the committees approving the protocols. For studies involving human subjects/samples, a statement must be included confirming that informed consent was obtained. Statistical analyses and information on the reproducibility of experimental results should be provided in a section titled “Statistics and Reproducibility”.

All Nature Cell Biology manuscripts submitted on or after March 21 2016 must include a Data availability statement as a separate section after Methods but before references, under the heading "Data Availability”. . For Springer Nature policies on data availability see http://www.nature.com/authors/policies/availability.html; for more information on this particular policy see http://www.nature.com/authors/policies/data/data-availability-statements-data-citations.pdf. The Data availability statement should include:

• Accession codes for primary datasets (generated during the study under consideration and designated as "primary accessions") and secondary datasets (published datasets reanalysed during the study under consideration, designated as "referenced accessions"). For primary accessions data should be made public to coincide with publication of the manuscript. A list of data types for which submission to community-endorsed public repositories is mandated (including sequence, structure, microarray, deep sequencing data) can be found here http://www.nature.com/authors/policies/availability.html#data.

• Unique identifiers (accession codes, DOIs or other unique persistent identifier) and hyperlinks for datasets deposited in an approved repository, but for which data deposition is not mandated (see here for details http://www.nature.com/sdata/data-policies/repositories).

• At a minimum, please include a statement confirming that all relevant data are available from the authors, and/or are included with the manuscript (e.g. as source data or supplementary information), listing which data are included (e.g. by figure panels and data types) and mentioning any restrictions on availability.

• If a dataset has a Digital Object Identifier (DOI) as its unique identifier, we strongly encourage including this in the Reference list and citing the dataset in the Methods.

We recommend that you upload the step-by-step protocols used in this manuscript to protocols.io. More details can found at https://www.protocols.io/help/publish-articles.

DISPLAY ITEMS – main display items are limited to 6-8 main figures and/or main tables for Articles, Resources, Technical Reports; and 5 main figures and/or main tables for Letters. For Supplementary Information see below.

FIGURES – Colour figure publication costs $600 for the first, and $300 for each subsequent colour figure. All panels of a multi-panel figure must be logically connected and arranged as they would appear in the final version. Unnecessary figures and figure panels should be avoided (e.g. data presented in small tables could be stated briefly in the text instead).

All imaging data should be accompanied by scale bars, which should be defined in the legend.
Cropped images of gels/blots are acceptable, but need to be accompanied by size markers, and to retain visible background signal within the linear range (i.e. should not be saturated). The boundaries of panels with low background have to be demarked with black lines. Splicing of panels should only be considered if unavoidable, and must be clearly marked on the figure, and noted in the legend with a statement on whether the samples were obtained and processed simultaneously. Quantitative comparisons between samples on different gels/blots are discouraged; if this is unavoidable, it should only be performed for samples derived from the same experiment with gels/blots were processed in parallel, which needs to be stated in the legend.

Figures should be provided at approximately the size that they are to be printed at (single column is 86 mm, double column is 170 mm) and should not exceed an A4 page (8.5 x 11"). Reduction to the scale that will be used on the page is not necessary, but multi-panel figures should be sized so that the whole figure can be reduced by the same amount at the smallest size at which essential details in each panel are visible. In the interest of our colour-blind readers we ask that you avoid using red and green for contrast in figures. Replacing red with magenta and green with turquoise are two possible colour-safe alternatives. Lines with widths of less than 1 point should be avoided. Sans serif typefaces, such as Helvetica (preferred) or Arial should be used. All text that forms part of a figure should be rewritable and removable.

We accept files from the following graphics packages in either PC or Macintosh format:

- For line art, graphs, charts and schematics we prefer Adobe Illustrator (.AI), Encapsulated PostScript (.EPS) or Portable Document Format (.PDF). Files should be saved or exported as such directly from the application in which they were made, to allow us to restyle them according to our journal house style.

- We accept PowerPoint (.PPT) files if they are fully editable. However, please refrain from adding PowerPoint graphical effects to objects, as this results in them outputting poor quality raster art. Text used for PowerPoint figures should be Helvetica (preferred) or Arial.

- We do not recommend using Adobe Photoshop for designing figures, but we can accept Photoshop generated (.PSD or .TIFF) files only if each element included in the figure (text, labels, pictures, graphs, arrows and scale bars) are on separate layers. All text should be editable in 'type layers' and line-art such as graphs and other simple schematics should be preserved and embedded within 'vector smart objects' - not flattened raster/bitmap graphics.

- Some programs can generate Postscript by 'printing to file' (found in the Print dialogue). If using an application not listed above, save the file in PostScript format or email our Art Editor, Allen Beattie for advice (a.beattie@nature.com).

Regardless of format, all figures must be vector graphic compatible files, not supplied in a flattened raster/bitmap graphics format, but should be fully editable, allowing us to highlight/copy/paste all text and move individual parts of the figures (i.e. arrows, lines, x and y axes, graphs, tick marks, scale bars etc.). The only parts of the figure that should be in pixel raster/bitmap format are photographic images or 3D rendered graphics/complex technical illustrations.

All placed images (i.e. a photo incorporated into a figure) should be on a separate layer and independent from any superimposed scale bars or text. Individual photographic images must be a minimum of 300+ DPI (at actual size) or kept constant from the original picture acquisition and not decreased in resolution post image acquisition. All colour artwork should be RGB format.

EXTENDED DATA FIGURES - When re-submitting your manuscript, please ensure that any supplementary figures and tables that are crucial to the manuscript's conclusions are converted into Extended Data figures and tables to increase visibility of these data. Extended Data figures and tables are online-only (present in the online PDF and full-text HTML versions of the paper), peer-reviewed display items that provide essential background to the article but are not included in the main article due to space constraints. A maximum of ten Extended Data display items (figures and tables) is permitted.

FIGURE LEGENDS – must not exceed 350 words for each figure to allow fit on a single printed NCB page together with the figure. They must include a brief title for the whole figure, and short descriptions of each panel with definitions of the symbols used, but without detailing methodology.

TABLES – main tables should be provided as individual Word files, together with a brief title and legend. For supplementary tables see below.

SUPPLEMENTARY INFORMATION – Supplementary information is material directly relevant to the conclusion of a paper, but which cannot be included in the printed version in order to keep the manuscript concise and accessible to the general reader. Supplementary information is an integral part of a Nature Cell Biology publication and should be prepared and presented with as much care as the main display item, but it must not include non-essential data or text, which may be removed at the editor's discretion. All supplementary material is fully peer-reviewed and published online as part of the HTML version of the manuscript. Supplementary Figures and Supplementary Notes are appended at the end of the main PDF of the published manuscript.

Supplementary items should relate to a main text figure, wherever possible, and should be mentioned sequentially in the main manuscript, designated as Supplementary Figure, Table, Video, or Note, and numbered continuously (e.g. Supplementary Figure 1, Supplementary Figure 2, Supplementary Table 1, Supplementary Table 2 etc.).

Unprocessed scans of all key data generated through electrophoretic separation techniques need to be presented in a supplementary figure that should be labelled and numbered as the final supplementary figure, and should be mentioned in every relevant figure legend. This figure does not count towards the total number of figures and is the only figure that can be displayed over multiple pages, but should be provided as a single file, in PDF or TIFF format. Data in this figure can be displayed in a relatively informal style, but size markers and the figures panels corresponding to the presented data must be indicated.

The total number of Supplementary Figures (not including the "unprocessed scans" Supplementary Figure) should not exceed the number of main display items (figures and/or tables (see our Guide to Authors and March 2012 editorial http://www.nature.com/ncb/authors/submit/index.html#suppinfo; http://www.nature.com/ncb/journal/v14/n3/index.html#ed). No restrictions apply to Supplementary Tables or Videos, but we advise authors to be selective in including supplemental data.

Each Supplementary Figure should be provided as a single page and as an individual file in one of our accepted figure formats and should be presented according to our figure guidelines (see above). Supplementary Tables should be provided

as individual Excel files. Supplementary Videos should be provided as .avi or .mov files up to 50 MB in size. Supplementary Figures, Tables and Videos much be accompanied by a separate Word document including titles and legends.

GUIDELINES FOR EXPERIMENTAL AND STATISTICAL REPORTING

REPORTING REQUIREMENTS – We are trying to improve the quality of methods and statistics reporting in our papers. To that end, we are now asking authors to complete a reporting summary that collects information on experimental design and reagents. The Reporting Summary can be found here https://www.nature.com/documents/nr-reporting-summary.pdf)If you would like to reference the guidance text as you complete the template, please access these flattened versions at http://www.nature.com/authors/policies/availability.html.

STATISTICS – Wherever statistics have been derived the legend needs to provide the n number (i.e. the sample size used to derive statistics) as a precise value (not a range), and define what this value represents. Error bars need to be defined in the legends (e.g. SD, SEM) together with a measure of centre (e.g. mean, median). Box plots need to be defined in terms of minima, maxima, centre, and percentiles. Ranges are more appropriate than standard errors for small data sets. Wherever statistical significance has been derived, precise p values need to be provided and the statistical test used needs to be stated in the legend. Statistics such as error bars must not be derived from n<3. For sample sizes of n<5 please plot the individual data points rather than providing bar graphs. Deriving statistics from technical replicate samples, rather than biological replicates is strongly discouraged. Wherever statistical significance has been derived, precise p values need to be provided and the statistical test stated in the legend.

Information on how many times each experiment was repeated independently with similar results needs to be provided in the legends and/or Methods for all experiments, and in particular wherever representative experiments are shown.

We strongly recommend the presentation of source data for graphical and statistical analyses as a separate Supplementary Table, and request that source data for all independent repeats are provided when representative experiments of multiple independent repeats, or averages of two independent experiments are presented. This supplementary table should be in Excel format, with data for different figures provided as different sheets within a single Excel file. It should be labelled and numbered as one of the supplementary tables, titled “Statistics Source Data”, and mentioned in all relevant figure legends.

--------- Please don't hesitate to contact NCB@nature.com should you have queries about any of the above requirements ---------

Version 2:

Decision Letter:

Our ref: NCB-A59350B

4th March 2026

Dear Dr. Ogrodnik,

Thank you for submitting your revised manuscript "Transcription-independent induction of rapid-onset senescence is integral to healing" (NCB-A59350B). It has now been seen by the original referee and their comments are below. The reviewer finds that the paper has improved in revision, and therefore we'll be happy in principle to publish it in Nature Cell Biology, pending minor revisions to satisfy the referee's final requests and to comply with our editorial and formatting guidelines.

We are now performing detailed checks on your paper and will send you a checklist detailing our editorial and formatting requirements in about 1-2 weeks. Please do not upload the final materials and make any revisions until you receive this additional information from us.

Thank you again for your interest in Nature Cell Biology. Please do not hesitate to contact me if you have any questions.

Sincerely,

Melina Casadio, PhD
Senior Editor, Nature Cell Biology
ORCID ID: https://orcid.org/0000-0003-2389-2243

Reviewer #5 (Remarks to the Author):

This is now the re-revised version in reply to my (reviewer #5) comments and concerns.

Following editorial guidance, the authors have undertaken major efforts to address many of my points, especially those listed as no. 5 and no. 6.

I congratulate the authors for providing novel evidence that helps to clarify issues, although open questions remain throughout the manuscript. Despite all efforts undertaken to extract key molecular mechanisms in early wound healing, the process in focus is highly dynamic and certainly not perfectly synchronized. The story appears quite bold and complex, and is likely to stimulate intense discussions within the scientific community rather than being perceived as definite evidence.

As a "late comer" to this already extensive review process and the additional experiments enforced thereby, I find this work now, in principle, mature enough and of sufficient quality to be published in NCB.

However, I strongly recommend another round of internal (and, perhaps, external) redacting of the data composition and phrasing (which should not involve expert reviewers anymore...) - there is still room for a more succinct, clear and sharpened presentation, which is essential for such data-dense work not to lose its readership in the middle of the main text, distracted by an endless load of supplementary figure material. This also includes or starts with the abstract, which could be much more scientific, i.e. referring to specific experimental cause-consequence findings (unlike the "...wounding causes cells to become senescent..." style), and skipping information of no direct value (such as "..., including epigen,...") for outlining the big picture.

Reviewer #5 (Remarks on Protocol(s)):

I did not review the methods in detail.

Version 3:

Decision Letter:

Dear Dr Ogrodnik,

I am pleased to inform you that your manuscript, "Transcription-independent induction of rapid-onset senescence is integral to healing", has now been accepted for publication in Nature Cell Biology.

Thank you for sending us the final manuscript files to be processed for print and online production, and for returning the manuscript checklists and other forms. Your manuscript will now be passed to our production team who will be in contact with you if there are any questions with the production quality of supplied figures and text.

Over the next few weeks, your paper will be copyedited to ensure that it conforms to Nature Cell Biology style. Once your paper is typeset, you will receive an email with a link to choose the appropriate publishing options for your paper and our Author Services team will be in touch regarding any additional information that may be required.

After the grant of rights is completed, you will receive a link to your electronic proof via email with a request to make any corrections within 48 hours. If, when you receive your proof, you cannot meet this deadline, please inform us at rjsproduction@springernature.com immediately.

You will not receive your proofs until the publishing agreement has been received through our system.

Due to the importance of these deadlines, we ask that you please let us know now whether you will be difficult to contact over the next month. If this is the case, we ask you provide us with the contact information (email, phone and fax) of someone who will be able to check the proofs on your behalf, and who will be available to address any last-minute problems.

If you have any questions about our publishing options, costs, Open Access requirements, or our legal forms, please contact ASJournals@springernature.com

Once your paper has been scheduled for online publication, the Nature press office will be in touch to confirm the details. An online order form for reprints of your paper is available at https://www.nature.com/reprints/author-reprints.html. All co-authors, authors' institutions and authors' funding agencies can order reprints using the form appropriate to their geographical region.

Publication is conditional on the manuscript not being published elsewhere and on there being no announcement of this work to any media outlet until the online publication date in Nature Cell Biology.

You may wish to make your media relations office aware of your accepted publication, in case they consider it appropriate to organize some internal or external publicity. Once your paper has been scheduled you will receive an email confirming the

publication details. This is normally 3-4 working days in advance of publication. If you need additional notice of the date and time of publication, please let the production team know when you receive the proof of your article to ensure there is sufficient time to coordinate. Further information on our embargo policies can be found here: https://www.nature.com/authors/policies/embargo.html

Please note that *Nature Cell Biology* is a Transformative Journal (TJ). Authors may publish their research with us through the traditional subscription access route or make their paper immediately open access through payment of an article-processing charge (APC). Authors will not be required to make a final decision about access to their article until it has been accepted. Find out more about Transformative Journals

Authors may need to take specific actions to achieve compliance with funder and institutional open access mandates. If your research is supported by a funder that requires immediate open access (e.g. according to Plan S principles or the NIH public access policy) then you should select the gold OA route, and we will direct you to the compliant route where possible. Because authors warrant under our subscription licensing terms that they haven't committed to licensing any version of their article under a licence inconsistent with the terms of our agreement – including the applicable embargo period – publication under the subscription model isn't suitable for authors whose funders require no embargo.

To assist our authors in disseminating their research to the broader community, our SharedIt initiative provides you with a unique shareable link that will allow anyone (with or without a subscription) to read the published article. Recipients of the link with a subscription will also be able to download and print the PDF.

If your paper includes color figures, please be aware that in order to help cover some of the additional cost of four-color reproduction, Nature Portfolio charges our authors a fee for the printing of their color figures. Please contact our offices for exact pricing and details.

As soon as your article is published, you will receive an automated email with your shareable link.

If you have not already done so, we strongly recommend that you upload the step-by-step protocols used in this manuscript to protocols.io (https://protocols.io), an open online resource that allows researchers to share their detailed experimental know-how. All uploaded protocols are made freely available and are assigned DOIs for ease of citation. Protocols and Nature Portfolio journal papers in which they are used can be linked to one another, and this link is clearly and prominently visible in the online versions of both. Authors who performed the specific experiments can act as primary authors for the Protocol as they will be best placed to share the methodology details, but the Corresponding Author of the present research paper should be included as one of the authors. By uploading your Protocols onto protocols.io, you are enabling researchers to more readily reproduce or adapt the methodology you use, as well as increasing the visibility of your protocols and papers. You can also establish a dedicated workspace to collect your lab Protocols. Further information can be found at https://www.protocols.io/help/publish-articles.

You can use a single sign-on for all your accounts, view the status of all your manuscript submissions and reviews, access usage statistics for your published articles and download a record of your refereeing activity for the Nature Portfolio.

Nature Cell Biology encourages authors presenting evidence for cell, biological, molecular, and genetic interactions to consider communicating these findings using Biofactoid (https://biofactoid.org/). This tool helps users share a searchable representation of interactions (e.g. binding, gene expression, post-translational modification) between genes, gene products, or chemicals. Information added to Biofactoid, with author attribution, is shared on social media and public databases, such as Pathway Commons, where it can be discovered and analyzed in the context of a large and growing corpus of knowledge.

Please feel free to contact us if you have any questions.

With kind regards,

Melina Casadio, PhD
Senior Editor, Nature Cell Biology
ORCID ID: https://orcid.org/0000-0003-2389-2243

Click here if you would like to recommend Nature Cell Biology to your librarian
http://www.nature.com/subscriptions/recommend.html#forms

** Visit the Springer Nature Editorial and Publishing website at www.springernature.com/editorial-and-publishing-jobs for more information about our career opportunities. If you have any questions please click here.**

Open Access This Peer Review File is licensed under a Creative Commons Attribution 4.0 International License, which permits use, sharing, adaptation, distribution and reproduction in any medium or format, as long as you give appropriate credit to the original author(s) and the source, provide a link to the Creative Commons license, and indicate if changes were made.
In cases where reviewers are anonymous, credit should be given to 'Anonymous Referee' and the source.
The images or other third party material in this Peer Review File are included in the article's Creative Commons license, unless indicated otherwise in a credit line to the material. If material is not included in the article's Creative Commons license and your intended use is not permitted by statutory regulation or exceeds the permitted use, you will need to obtain permission directly from the copyright holder.
To view a copy of this license, visit https://creativecommons.org/licenses/by/4.0/

Response letter

Reviewer #1 (Remarks to the Author):

The authors have addressed all of my concerns and the manuscript is greatly improved. Congratulations.

Thank you very much, we are very grateful for your appreciation of our work!

Reviewer #2 (Remarks to the Author):

I commend the intensive efforts of the authors in expanding the manuscript, particularly in clarifying the p21-lineage tracing system, improving figure consistency, and integrating additional molecular data. Despite these enhancements, I remain unconvinced that the revision sufficiently addresses the core mechanistic questions. Moreover, recent literature raises notable contradictions. Gasek et al have recently demonstrated that clearing p21-high senescent cells actually expedites wound closure, mediated at least in part by NF-κB inhibition. This finding directly opposes the primary assertion of the current manuscript, which posits that $p21^+$ senescent cells are essential for wound healing. Unfortunately the authors merely cite and mention this recent manuscript without actually offering any potential explanation for the opposite results.

The article by Gasek et al., was published as a short communication and was submitted several months after our pre-print was published. We have already provided a detailed explanation of how differences in the model, study design, and the robustness of the experimental setup likely account for the discrepancies in the findings in the previous response letter. Furthermore, all these differences pertain to only one specific aspect of our study: the impact of p21+ cells on the rate of healing. Our study extends beyond this topic by exploring the rapid induction of senescence and its underlying mechanisms, among other aspects.

In our response letter, we included a whole page of detailed explanations and presented several lines of evidence clarifying the differences between the two studies. Moreover, the publication was cited in the revised manuscript as the reviewer requested. It remains unclear to us what additional measures would be considered suitable to address the differences that short-report study submitted after our original submission reported.

Major points of concern:

1. Although additional markers have been provided, the work still lacks functional validation of true, irreversible senescence. The lineage tracking shows persistence, but may equally reflect reporter stability. It remains a stretch to define thi cell population as fully senescent.

*To date, there has not been a single study demonstrating anything that meets the reviewer's definition of "*true, irreversible senescence*" in vivo. Our study provides, to the best of our knowledge, the most comprehensive characterization of senescence in vivo, presenting strong evidence of multiple senescence markers coexisting in a population of cells exhibiting stable cell cycle arrest. The reviewer's failure to suggest any methods to demonstrate the purported "*true, irreversible senescence*" suggests that this point is raised primarily to cast doubt on our findings.*

Our methodology for identifying cellular senescence fully meets the criteria established by the international research community, as detailed in our recent Cell publication (PMID: 39121846).

2. Despite added experiments, the link between basal p21 mRNA levels and rapid protein induction remains inferential. Transcription-blocking agents are not ideal controls; translation inhibition suppresses all protein synthesis, not just p21. The RNA-protein binding data remain correlative and descriptive. More direct manipulation is needed to support a causal model.

*Once again, it is unclear what the reviewer means by requiring "*direct manipulation is needed to support a causal model*". If "*Transcription-blocking agents are not ideal controls*" we would appreciate the reviewer's suggestion on what would constitute an ideal control in this context. We have conducted numerous experiments to support this finding, so it is truly disappointing that the reviewer remains unsatisfied and has not offered any constructive alternatives.*

3. The pulse-chase tracing with tdTomato lacks power and fails to rigorously prove that the same keratinocytes harboring high baseline p21 mRNA are tracked and eliminated. The rare occurrence of $tdTom^+/p21^-$ clusters undermines fidelity of the system. The possibility of reporter persistence or artifact remains unaddressed.

*The findings we have described on the pulse-chase tracing with tdTomato are in almost all cases of $p<0.0001$. How could that be anyhow indicative that this set of experiments "*lacks power*"?*

*The statement "*The rare occurrence of $tdTom^+/p21^-$ clusters undermines fidelity of the system.*" is just so unfair. To maintain full transparency, we reported a single instance (out of hundreds of images analyzed) of $tdTom^+/p21^-$ cells, yet the reviewer uses this as an argument against the validity of our system. No lineage tracing system is 100% accurate, and the one we used approaches that level of fidelity and should not be dismissed as unreliable. Again, it remains unclear what the reviewer means by "*The possibility of reporter persistence or artifact remains unaddressed.*" since no constructive suggestions have been provided on how to address this concern.*

4. While the authors suggest that mTOR inhibition reduces p21 induction, this remains superficial pharmacology without mechanistic clarity. The notion of transcription-independent initiation followed by transcription-dependent reinforcement is interesting but not empirically distinguished. The actual signaling triggers such as mechanosensing, osmolarity, or others are purely speculative.

*This finding is just one among many that address the criticism regarding insufficient evidence for the upstream pathway driving rapid-onset senescence in wounds. We are confident in this result, supported by experiments using two pharmacological agents. Once again, it is unclear what the reviewer means by "*superficial pharmacology without mechanistic clarity*" as to our knowledge, this finding cannot be validated by other methods. Throughout the project, we have employed several approaches beyond pharmacology to support and verify our results, including the use of three different transgenic mouse models, which do not rely on pharmacological interventions.*

Excluding results like IL-6 (reported as "irregular and scattered") without robust validation or explanation raises concerns about selective reporting. Both positive and negative findings need transparent inclusion to avoid bias.

*We have reported all the experiments conducted during the revision process, including those that did not yield sufficiently reliable results. This approach is the opposite of "*selective reporting.*" Out of six probes we purchased, established, and validated, four did not produce results reliable enough to include in the manuscript, but we described them in the rebuttal letter. Again, it remains unclear what form of "*robust validation or explanation*" beyond what is already provided in the rebuttal letter the reviewer expects to see that would prevent our findings from being published. We are happy to add further descriptions, controls, or validations as needed, but the purpose of such additions is not clear to us. During the revision, we included data on two additional transcripts/probes, primarily Ibfbp3 and Adam8, and we do not understand what kind of "*transparent inclusion*" the reviewer believes these findings require.*

Reviewer #3 (Remarks to the Author):

The authors have made a comprehensive work in revising the manuscript. While they might not have addressed each and any of the sggestions of all the reviewers, the manuscript has improved in this revision and I am satisfied

Thank you very much! We sincerely appreciate the reviewer's recognition of our work on the manuscript and throughout the revision process.

Reviewer #4 (Remarks to the Author):

While the authors had tried (often unsuccessfully) to address the different questions raised during the initial review, the conceptual novelty of this manuscript remain extremely limited. If we replaced the word senescence by terminal differentiation, most of the data have been previously described.

*It is unclear why the reviewer describes the manuscript as having "*extremely limited*" conceptual novelty, especially without specifying the basis for this assessment. From our understanding of the manuscript, and after numerous consultations with leading experts in the field, we believe the manuscript presents significant conceptual novelty. This includes the mechanistic characterization of rapid-onset senescence, the impact of senescent cells on the healing process, and the molecular details of how the p21 (Cdkn1a) transcript is regulated under homeostatic conditions and activated upon wounding, all of which are entirely novel.*

As for "If we replaced the word senescence by terminal differentiation, most of the data have been previously described.*", statement such as "If we replaced the word xxx by yyy, most of the data have been previously described." could be stated for essentially any great discovery up to date.*

Unfortunately, the mechanistic insights about the sequestration and rapid translation of p21 upon wounding had not been significantly improved. As it stands, this manuscript would be better suitable for a more specialized journal such as Nat Communications.

We performed several experiments to strengthen the conclusions regarding the sequestration and rapid translation of p21 upon wounding to the best of our ability. To our knowledge and understanding, providing further details beyond this is nearly or completely impossible. For example, the proteomics experiment on p21 mRNA-binding factors in vivo took us a year to complete and was only feasible in pigs due to the large number of cells required. While a study using this methodology was previously published in Cell (PMID: 25843628), it was limited to in vitro conditions and we are the first to successfully apply this method in vivo.

That said, mechanistic interventions in this system are not possible, as generating transgenic pigs or supplying sufficient drug for whole-body treatment is unfeasible. Even if we were to create a new set of transgenic mice with modified p21-binding factors, performing the proteomics experiment in mice would require sacrificing hundreds (at the very least) of animals, with a low likelihood of success. This approach would be unproductive and raise significant ethical concerns. The findings we report on the sequestration and rapid translation of p21 during wound healing already represent a substantial advance. It is unclear why this has not been recognized.

Overall, we remain open to improving the manuscript, but it is unclear what specific doubts reviewers 2 and 4 have regarding the validity and impact of our results. The absence of constructive feedback raises concerns about potential conflicts of interest. Therefore, we kindly request responses from the reviewers addressing these concerns or permission to publish our research results.

Response Letter

We are very grateful to the reviewer for taking time and effort to thoroughly review our research. But especially, we would like to thank for the positive assessment of our work. It has been a challenging manuscript to communicate to the research community, with some researchers who loved it, others (despite our extensive evidence) who hated it, which has been reflected in the response of the earlier reviewers.

The editors of Nature Cell Biology considered your feedback positively and we were given 4-8 weeks to address the comments and advised to focus primarily on addressing point 5 regarding p53 levels and p21 kinetics but especially on tackling point 6 regarding the protein levels of Cdkn1a mRNA binding partners.

Within the given timeframe, we have been working extensively on covering as many points and providing as reliable data as possible. That resulted in additions, among others:

A. *We managed to run proteomics on porcine acute excision wounds samples that were generated in an alike manner that samples on which proteomics of Cdkn1a/p21 mRNA binding partners was done. These results are now included in the manuscript and described in detail below (about bulk proteomics itself) and in response to point 6.*
   "To further verify that changes in the abundance of proteins bound to *Cdkn1a* mRNA are not due to overall alterations in protein levels following injury, we performed proteomic analysis of porcine excision injury samples collected using the same procedure as for the *Cdkn1a* mRNA samples, at two time points: 15 min and 4 h post-injury (Extended Data Fig. 12; Extended Data Table 6). The proteomic analysis identified nearly 8,000 proteins, of which approximately 5,000 were quantified with high confidence, based on sufficient peptide coverage and protein intensity (Extended Data Table 6). As expected, minimal changes were detected at 15 minutes post-injury (Extended Data Fig. 12a, b), while at 4 hours post-injury, we observed significant upregulation of 477 proteins and downregulation of 262 proteins (Extended Data Fig. 12a, c). GO term enrichment analysis revealed that proteins upregulated at both early and late time points were primarily associated with coagulation and immune cell infiltration (Extended Data Fig. 12d, e). In contrast, proteins downregulated at the late time point (no proteins were significantly downregulated at the early time point) were mainly linked to translation (Extended Data Fig. 12f), consistent with previously reported rapid translational responses to injury (PMID: 37098351; PMID: 37495694)."
   How these results relate to the proteomics of the Cdkn1a mRNA protein binders is described in response to point 6, briefly, <u>we observed that (bulk) protein levels of Cdkn1a mRNA binding partners do not change in a manner that corresponds to the changes we observed in Cdkn1a mRNA-bound proteins suggesting that these changes are indeed due to dissociation and binding to p21 mRNA and not due to the changes in levels of these proteins in bulk</u>. We also used this dataset to investigate FoxO/TGF-β-Smads pathway (for details see response to point 5).

B. *We performed several staining procedures and biochemical assays, as well as analyses and re-analyses of existing materials, to provide supporting evidence for rapid-senescence induction in cell types beyond keratinocytes and tissue types beyond dorsal skin. Our work for this revision also aimed to assess pathways requested for evaluation regarding their association with rapid-onset senescence induction, investigate the relationship between p16/Cdkn2a expression levels and the temporal kinetics of the injury response, and determine the timing required for Tamoxifen to induce tdTomato expression, among other aspects. The results are described in detail in the responses to points 2, 4, and 5.*

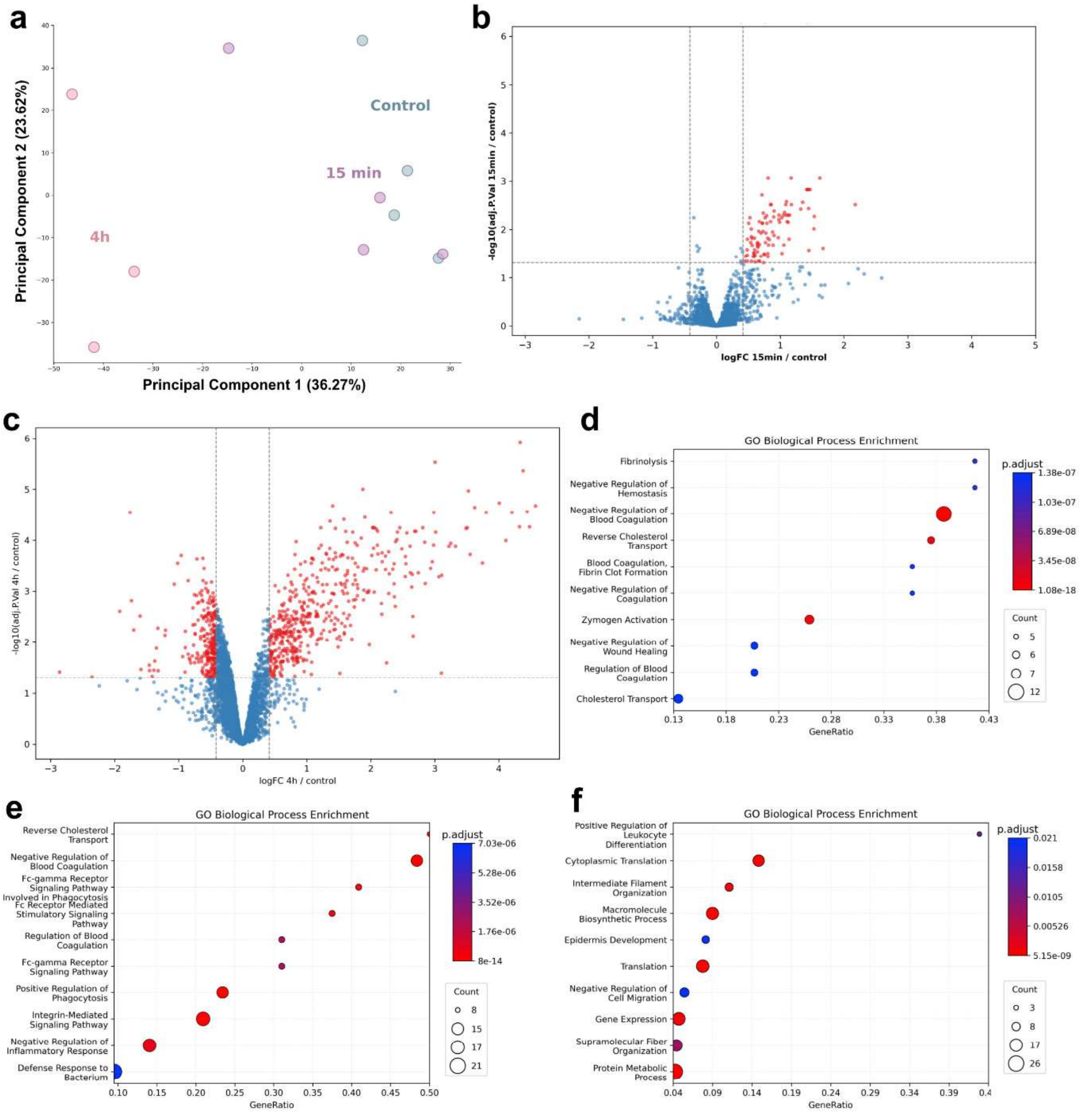

(**a**) Principal Component Analysis (PCA) of proteomics results obtained from porcine excision injuries at two timepoints, a 15 min timepoint ("short") and a 4 h timepoint ("long"), as well as from control skin samples.

(**b**)–(**c**) Volcano plots showing protein abundance at the (**b**) 15 min and (**c**) 4 h timepoints following excision injury compared to control samples. Proteins that were significantly different (adjusted p value < 0.05 and $\log_2$FC > 1.33 or < –1.33) are highlighted in red; all others are shown in blue.

(**d**)–(**f**) Dot plots illustrating Gene Ontology (GO) - enriched Kyoto Encyclopedia of Genes and Genomes (KEGG) term "Biological Process Enrichment" for (**d**) upregulated proteins at the 15 min timepoint, (**e**) upregulated proteins at the 4 h timepoint, and (**f**) downregulated proteins in porcine excision wounds.

Proteomics analyses were performed on samples from n = 4 pigs for the control and 15 min timepoint conditions, and n = 3 pigs for the 4 h timepoint.

C. *We conducted thorough bioinformatics analysis of the binding properties between Cdkn1a mRNA and several candidate proteins expected to be involved in the rapid onset induction of p21. The detailed outcomes of these investigations are described in the response to point 6 and parts of the analyses are included as the Extended Data Figure 11f-g. Briefly though, we found that SRSF3 and hnRNPA1 are highly likely to bind to the 3'UTR region of the Cdkn1a mRNA.*

D. *Conducted several in vivo experiments in pigs to collect samples for RIP-qPCR (see point F and the response to point 6 below), as well as for late time points (8 h and 10 h) after excision injury to determine the detailed kinetics of Cdkn1a induction (see the response to point 5 below). The latter experiments demonstrated for the first time that while p21 protein is induced independent of transcription as early as ~90 min post-injury, Cdkn1a mRNA levels increase later, at around 8 h post-injury.*

E. *We performed in vivo experiments in mice to obtain a more detailed kinetic profile of tdTomato induction following Tamoxifen administration in p21-CreER-R26-tdTomato mice. The details are described in our response to point 5, but briefly, we used seven p21-CreER-R26-tdTomato mice and two wild-type mice to show that tdTomato induction occurs in less than 24 hours (and later than in 6 hours). We also conducted a pilot experiment on aged and young mice to assess whether there are any differences in the rapid-onset induction of p21 upon injury. Here we observed only a trend for aged skin showing fewer p21+ cells in 6h wounds.*

F. *The most challenging and high-risk, yet probably the most directly relevant, experiment addressing the reviewer's request to verify the binding capacity of SRSF3 to Cdkn1a mRNA was the RNA immunoprecipitation followed by quantitative PCR (RIP-qPCR). Given the limited time available, it was extremely difficult to obtain all required samples and reagents, not to mention the several-week duration of the procedure. Fortunately, we succeeded in organizing an in vivo pig experiment, from which we collected 198 samples of excision wounds (the maximum number allowed by our animal ethics license) and 198 samples of control skin. We were initially concerned that this material might still be insufficient to immunoprecipitate SRSF3 and yield adequate Cdkn1a mRNA for subsequent RT-qPCR analysis. However, with all controls confirming the experiment's validity, we can confidently state that our hypothesis has been verified: SRSF3 binds approximately 50% less Cdkn1a mRNA after injury compared with healthy control skin. These findings are further described in the response to Point 6 and illustrated in Figure 6g and Extended Data Figure 13c. Detailed methodology is provided in the Extended Data document, and the raw western blot data are included in the file "Source Data – Unprocessed Western blots and gels." Overall, this result further substantiates the specificity of the SRSF3–Cdkn1a mRNA interaction and supports the proposed mechanism of Cdkn1a mRNA dissociating from SRSF3 upon injury, as requested by the reviewer.*

G. *A large number of in situ experiments and analyses were conducted including RNA-ISH for Cdkn1a, IHC and IHF for Smad2, p53, and p21, among others.*

H. *Added source data for all gels (Western blots and RNA blots) and for all quantitative datasets, now provided as separate files: "Source Data – Unprocessed Western blots and gels" and "Source Data – Statistical data for figures," respectively.*

I. *Implemented numerous quality improvements to the manuscript, including providing more detailed figure legends, updating and expanding references, and refining figure design.*

Reviewers' Comments:

Reviewer #5 (Remarks to the Author):

Valdivieso K,... ...Ogrodnik M, Transcription-independent induction of rapid-onset senescence is integral to healing Submitted to Nature Cell Biology

This is an additional comment on an ongoing controversial debate between two of the four consulted referees (#2 and #4) and the authors. As a late-entering expert #5, I provide here focused comments on this controversy and my overall impression of the manuscript – with the caveat that I had no access to the Supplementary Information/Extended Data Files.

The central observation of this work is the unexpectedly early detectability of high-level p21 protein-expressing keratinocytes just hours after skin injury, interpreted as rapid-onset senescence during wound healing by the authors. The key claim is the transcription-independent induction of p21 protein expression via immediate translation of pre-elevated p21 transcripts in the nucleus.

With respect to referee #2:

- Clashing evidence recently published in a different paper (Gasek et al., Nat Aging, 2024): This should be appropriately discussed here

- Lacks functional validation of true, irreversible senescence: I do not see this as a fundamental concern; please find my points on other types of cellular senescence below.

- Evidence on basal p21 mRNA levels and rapid protein induction: I partly agree, see my points below

- Technical issues with tdTomato-governed pulse-chase tracing: I partly agree, see my points below

- Impact of mTOR inhibition on p21 induction: I partly agree, see my points below

- Excluding results like IL-6: I cannot comment, this did not come up to my attention

Thank you very much for supporting our research work. Accordingly, we expanded the discussion on the results provided by Gasek et al., (2024). The text is in the discussion section of the manuscript and provided below:

*"*A key distinction between that study and our findings is that the authors report their construct selectively eliminates "p21–highly expressing" cells. In our study, p21-ATTAC mice undergo targeted apoptosis initiated by FKBP dimerization and subsequent caspase-8 activation, whereas in p21-DTA mice, cell death is induced through inhibition of protein synthesis. Although that study reports reduced tdTom and p21 protein synthesis, the efficiency of selective elimination of p21-positive cells remains unclear. Moreover, as those mice were treated daily with tamoxifen, unlike most of our experiments, which employ a single administration (with the exception of UC2288), this dosing regimen may have been sufficiently intense to elicit side effects, as previously reported (PMIDs: 22001866 and 32943672), potentially influencing wound healing. This finding highlights the need for further comparisons between models and treatment protocols, and we remain optimistic that future follow-up studies using these mice will provide more detailed molecular characterization, facilitating a more comprehensive comparison.*"*

Moreover, we addressed all the other points, and their description is provided as responses to the points below.

With respect to referee #4:

- Conceptual novelty of this manuscript remains extremely limited: I disagree; I find the primed-for-senescence concept with pre-elevated p21 transcripts appealing and novel

- Mechanistic insights about sequestration and rapid translation of p21 upon wounding: I cannot comment on improvements during review, but see this topic addressed, albeit with some concerns, as detailed below

Thank you very much for supporting the publication of our research discoveries.

In essence, extensive time and resources have been invested so far to improve the manuscript based on previous statements by four referees. I share some but not all concerns of the two colleagues that remain reserved. However, my perception of the manuscript is that a thorough textual revision including better presentation of the figure material (where, how, and legends) may substantially strengthen this manuscript – which will advance the field, not without stimulating discussions and controversies, but adequate for the high level of NCB.

We are extremely grateful for appreciating the value of our work and the time/resources we spent to further improve it throughout the revisions. We have now further adjusted and improved the manuscript closely following the feedback of the reviewer, with the point-by-point details described below.

Accordingly, we added changes to the text, figures and legends to make the manuscript easier to follow.

Major concerns and comments

1. It is intrinsic to the claim of "rapid-onset senescence" that not all features of a senescent cell (morphology, epigenomic remodeling, lysosomal changes, dynamics of the secretome over time etc.) will be found in cells that start to exhibit signs of commencing senescence unusually early. The authors should state this more carefully. Whether these early-senescent cells would potentially become truly and full-featured senescent at later times remains rather philosophical, since the fate of these keratinocytes is to be rapidly turned over by shedding anyway, irrespective of their senescent-or-not status.

   We couldn't agree with the reviewer more. Indeed, while we found numerous markers of cellular senescence being upregulated rapidly upon injury, it is likely there are some markers that are not upregulated as rapidly and take some time to be established. As at least some of these, such as epigenetic modifications, are very challenging or even impossible methodologically to be done spatially on a single-cell level, we are hopeful that future research will reveal the distinct trajectory of changes starting from the rapid senescence induction. In this respect, we allowed ourselves to merge the thoughts of the subject of the Review and our own and we have added the following text to the discussion part of the manuscript:

   *"*The concept of "rapid-onset senescence" inherently implies that not all canonical features of senescent cells, such as epigenomic remodeling, lysosomal alterations, and temporal dynamics of the secretome, will be present in cells that begin to exhibit early signs of senescence. While we observed a rapid upregulation of numerous senescence markers following injury, it is likely that some features require more time to become established. As certain characteristics, including epigenetic modifications, are technically challenging or even

currently infeasible to assess spatially at the single-cell level, we remain hopeful that future studies will elucidate the distinct temporal trajectory of molecular changes associated with rapid senescence induction.*”*

Of note, the authors initially also report detection of p21-high fibroblasts and myocytes, but do not follow up on those more wound-resident cells later.

Indeed, throughout the manuscript we have prioritized the proof-of-concept description of rapid-onset senescence of keratinocytes, yet, when possible and appropriate, tried to relate to other types of cells. In regard to fibroblasts, we observed that a fraction of them become positive in porcine skin excision injury (see below and Extended Data Figure 6a) and mouse ear excision injury (see response to Point 2 and Extended Data Figure 7).

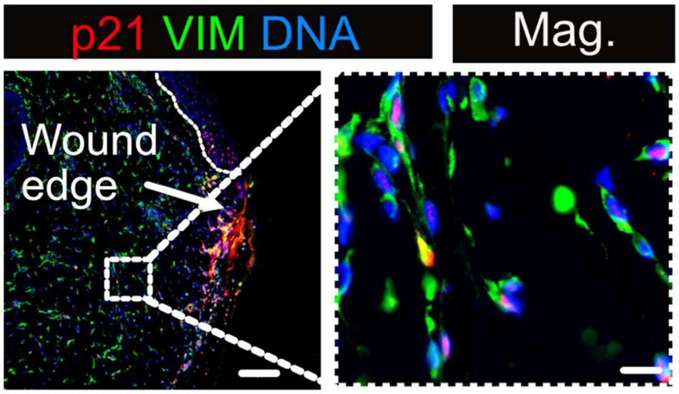

Representative images of p21 (red) and Vimentin (VIM; green) in porcine skin at 3 h after injury. DNA is stained with DAPI (blue) and dashed white line marks basal lamina of epidermis. The scalebars show 100 µm, for their micrographs scale bars are 10 µm.

Moreover, we found that at 1.5 hours post-injury, a time point when overall Cdkn1a transcript levels are not yet increased (Extended Data Figure 9g-j), those fibroblasts that became p21-positive already exhibited higher baseline levels of Cdkn1a (see below and Extended Data Figure 9e-f).

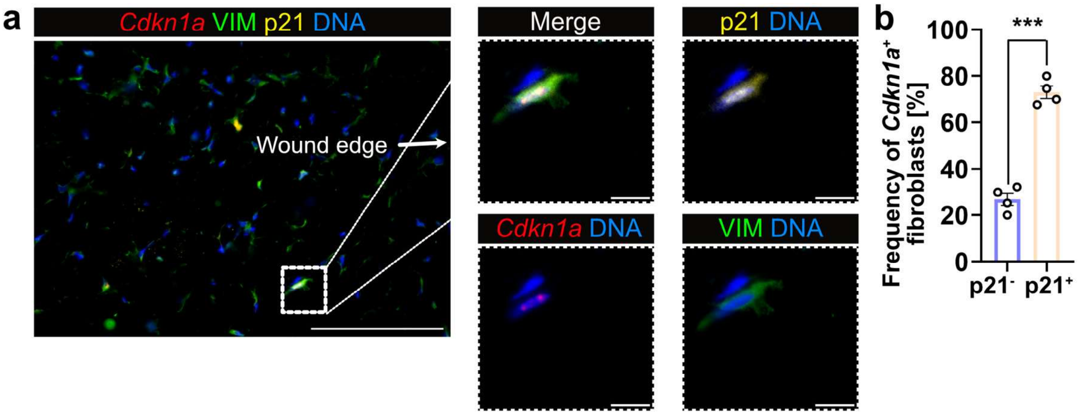

(**a**) A representative image of an Immuno-RNA-ISH staining against *Cdkn1a* (red), Vimentin (VIM; green) and p21 (yellow) of a sample from porcine injury 1.5 h after wounding.
(**b**) Quantification of the frequency of *Cdkn1a*$^{+}$ fibroblasts that are p21^{+} or p21^{-}.
DNA is visualized by DAPI (blue). Mean ± SEM plotted. Data are from n = 4 pigs per group. An unpaired t-test was used. The scale bar of the main image shows 100 µm, for its magnifications the scale bar is 10 µm. *** is p<0.001

Likewise, we observed fibroblasts becoming tdTom+ in p21-Cre-R26-tdTomato mice, and similarly to what observed in keratinocytes, with properties of stable cell cycle arrest (see below and Extended Data Figure 8 a and c). These fibroblasts persist for ~2 weeks after injury and afterwards are reduced in number, likely due to induction of apoptosis (Extended Data Figure 8 l-m).

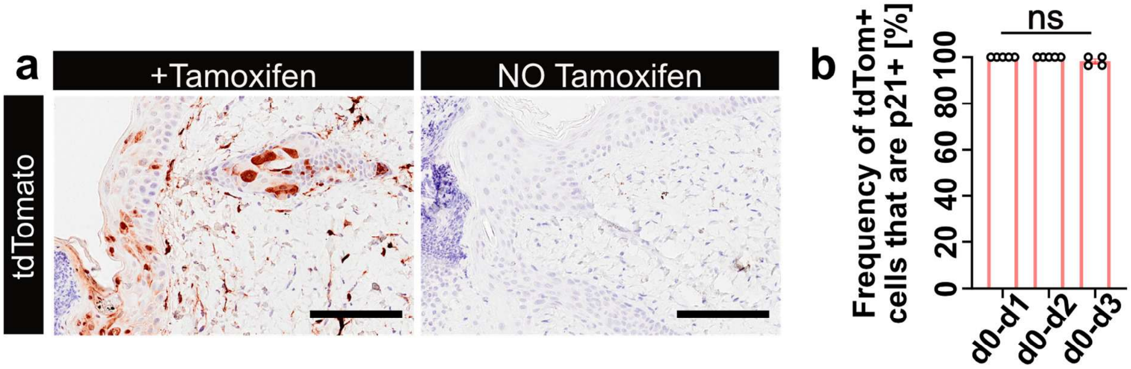

(**a**) A representative image of an IHC staining against p21 in mouse skin 3 days after injury and a single Tamoxifen injection and on the right side is the same staining done in a mouse skin injury that did not receive Tamoxifen.
(**b**) Quantification of the frequency of tdTom^{+} fibroblasts that are p21^{+} in days 1-3 following injury and Tamoxifen injection.
Data are from n = 4-5 mice per group. Mean ± SEM plotted. One-way ANOVA with post-hoc Dunnett's test was used. The scale bars show 100 µm. Ns stands for non-significant.

Utilizing porcine skin, while having advantage of high similarity to human skin, a lot of material to work with, comes with a limitations that these animals (similarly as humans) lack subcutaneous muscle, thus we did not investigate myocytes beyond earlier experiments in mouse skin. Instead, we provide breadcrumbs for future researchers that also smooth muscle cells of blood vessels can become p21-positive shortly upon injury (see below and Extended Data Figure 6b).

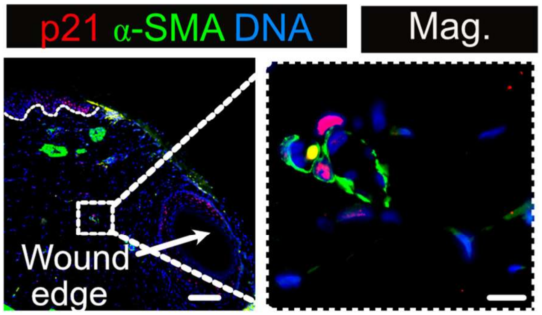

Representative images of p21 (red) and alpha smooth muscle actinin (α-SMA; green) in porcine skin at 3 h after injury. DNA is stained with DAPI (blue) and dashed white line marks basal lamina of epidermis. The scalebars show 100 µm, for their micrographs scale bars are 10 µm.

It should also be noted that some of the methods we employed, such as bulk proteomics and proteomics of p21 mRNA-binders did not allow for single-cell approach and thus they might represent an average response of several cell types being rapidly induced to senescence. Overall, we are hopeful that having a thorough description of injury-induced rapid onset senescence of keratinocytes and in bulk, with preliminary leads on these phenomena being consistent for other types of skin cells, will lead to other research groups investigating this phenomenon further and exciting discoveries on the physiology of cellular senescence in vivo.

While p21 is essential for keratinocyte senescence (Missero et al., Genes Dev 1996), more information on paralleling or delayed kinetics of the other central cell-cycle-inhibiting senescence indicator/mediator p16INK4a would have been appreciated.

We thank the reviewer for pointing this out, and while we have been trying to be as thorough as possible when it comes to establishing the relationship of p16INK4a to senescence in wounds. We even utilized a dedicated mouse model (kindly provided by Prof. Norman Sharpless), p16-tdTomato. While we were able to detect an increase in mRNA for p16 (Cdkn2a) and tdTomato several days after injury, the levels of both were extremely low, and we did not find any evidence for expression of p16 or tdTomato proteins (which we are convinced is not due to our methodology, as we were able to detect tdTomato in p21-cre-R26-tdTomato mice). These are presented below and Extended Data Fig. 3h-i)

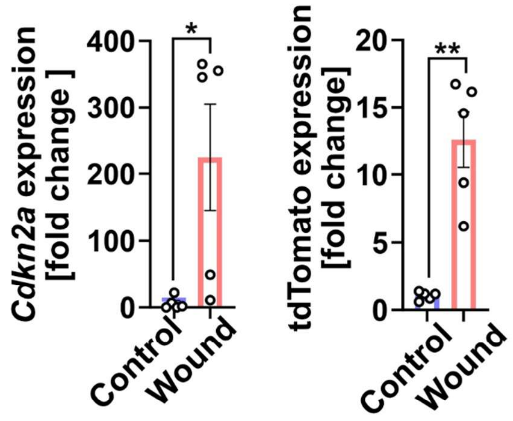

RT-qPCR results of **(left side)** *Cdkn2a* (p16) and **(right side)** *tdTomato* expression at 7 days after wounding of p16-tdTomato mice when compared to the unwounded skin of p16-tdTomato mice.

In our sc-RNA-seq transcriptomics data, we were able to detect Cdkn2a/p16 increasing in fibroblasts during aging and keratinocytes and fibroblasts in wounds 1-3 weeks post injury (see below and Extended Data Figure 3j-k).

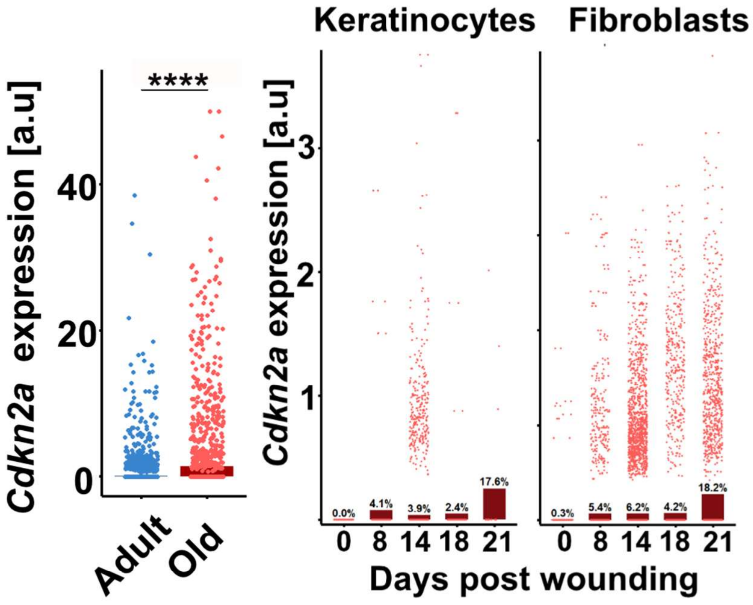

(**left graph**) The average expression levels of Cdkn2a among fibroblasts from skin samples from adult and old mice.

(**right graphs**) Average *Cdkn2a* expression levels among keratinocytes (left side) fibroblasts (right side) from samples of control and wounded mouse skin used to create the sc-RNA-seq mouse skin atlas. The wound samples were split to represent the timepoints at which the samples were collected. Each dot represents an individual cell, and an average is shown as a bar-graph. Values above each bar show the % of population positive the presence of *Cdkn2a* transcript.

Overall, while we cannot exclude that we missed something, this data implies that Cdkn2a/p16 is expressed in skin cells of wounds yet, only at later points after injury.

2. It would have helped to elaborate more on the underlying cell biologic, molecular, biomechanic and biochemical aspects of the injury set here to initiate the wound healing process. Excision and micro needle are the technicalities, but what is the actual damage inflicted? How exclusive is this procedure to the injured skin, as compared to the surgically wounded gut mucosa and wall during anastomosis healing, or liver damage after puncture etc.?

Thank you very much for this suggestion. We are hopeful that this study will serve as a foundation for future research on how exactly damage propagates and whether rapid-onset senescence is selective to our outer layer or could also occur in inner organs, such as during wounding of gut or liver. In this respect, injuries to the liver, brain, and other organs in which the induction of cellular senescence has been implicated (PMID: Liver: 18724938, 35708907, 32139422; Brain: 34650425, 32508743, 33964983) should be exciting to research next.
*In relation to the question "*what is the actual damage inflicted*" our earlier research (Ring et al., 2023 Developmental Cell; PMID: 37098351) shows that damage-associated molecular patterns (DAMPs) are at the heart of the rapid responses following the injury and we expect those to be involved in the induction of the rapid-onset senescence as well. That said, we have not yet developed any reliable in vivo or in situ model where we could modulate the level or propagation of DAMPs, in wounds to directly address this question.*
*We added a paragraph to the discussion: "*We are hopeful that this study will also provide a foundation for future investigations into the mechanisms of damage propagation and the contextual selectivity of rapid-onset senescence. In this respect, our earlier research (PMID: 37098351) showed that damage-associated molecular patterns (DAMPs) are at the heart of the rapid responses following the injury, and we expect those to be involved in the induction of the rapid-onset senescence as well.
It also remains to be determined whether the phenomenon of rapid-onset senescence is restricted to dorsal skin and ears or whether it can also occur within internal organs, such as the liver or gut, in response to injury. In this regard, examining injuries to the liver, brain, and other organs in which the induction of cellular senescence has previously been reported (PMID: liver—18724938, 35708907; brain—34650425, 32508743) represents a particularly promising direction for future research*".*
Moreover, for the purposes of the revision, we have established methods to detect rapid senescence in aural (ear) injuries. Our model involves creating a circular punch wound through the ear tissue (see below).

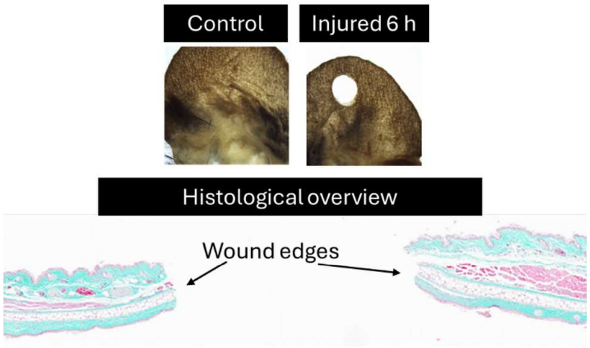

Photographs of the murine aural injury (top panel) and representative histological images (Trichrome-stained) of samples collected 6 hours after injury (bottom panel).

Consistent with our observations from excisional injuries of the dorsal skin, we found that ear tissue also exhibits a rapid upregulation of p21 at the edge of the injury, primarily in the epidermis and dermis, although occasional cartilage cells also become positive. These results are shown below and in the Extended Data Figure 7.

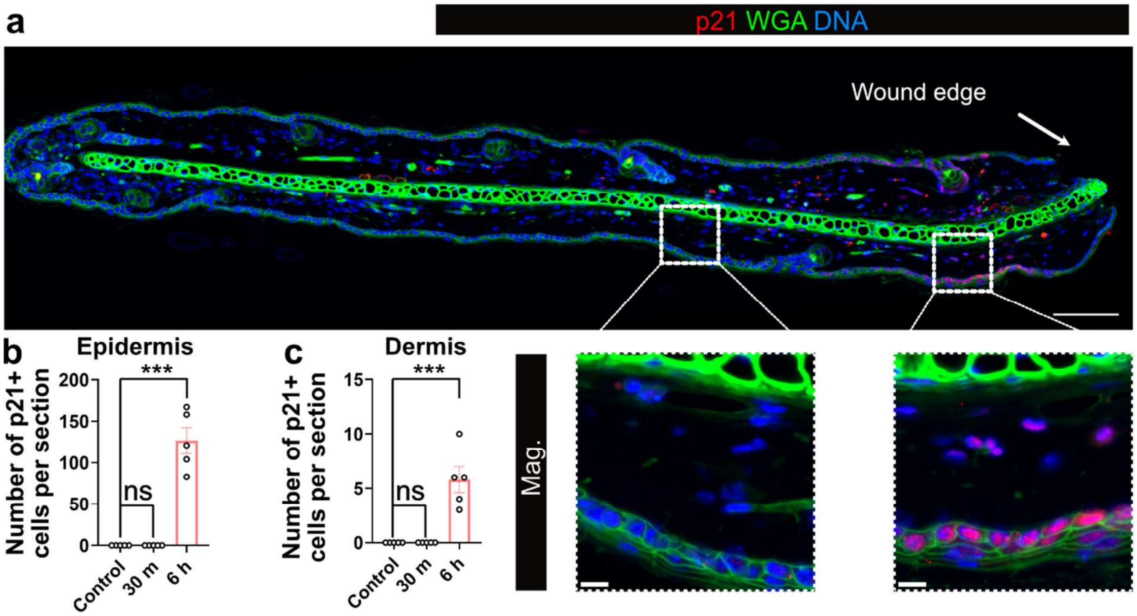

a) Representative images of p21 (red) and WGA (Wheat Germ Agglutinin; green) staining in samples of murine aural injury at 6 h post-wounding. DNA is stained with DAPI (blue). The scalebars show 100 µm, for their micrographs scale bars are 10 µm.
b) Quantification of the number of p21$^+$ cells per section in the epidermal compartment next to the injury in the control, unwounded region as well as in injuries at 30 min and 6 h.
c) Quantification of the number of p21$^+$ cells per section in the dermal compartment next to the injury in the control, unwounded region as well as in injuries at 30 min and 6 h.
Data are from n = 5 mice. Mean ± SEM plotted. For all the graphs one-way ANOVA with post-hoc Dunnett's test. ***p<0.001 and "ns" is non-significant.

How comparable is mechanic injury to UV light- or gamma-irradiation-mediated skin damage, or other types of drug-induced senescence by systemic (e.g. chemotherapeutics, or CCl4-mediated hepatotoxicity) or topically applied drugs (such as DMBA/TPA to induce Ras-senescent lesions)? I'm not asking for all those experiments, but would like to see the work presented in the wider scope of the senescence field. The discussion only superficially touches upon muscle and bone healing. It seems that the skin comes with specific properties not applying to other damage-induced senescence scenarios – and this might be its pre-exposure to UV light, potentially explaining the pre-elevated p21 transcript expression as sort of a senescence-primed/-prone standby mode. Hence, experiments on long-term light-deprived skin or mice would have potentially been insightful.

Thank you for this valuable comment. In our experimental setups, we primarily used pig skin, which is arguably light-exposed, as well as skin from C57BL/6 mice, which, due to the presence of black hair, is likely to be much less light-exposed. Thus far, rapid-onset senescence has been consistently induced in both models. The majority of differences we observed in the extent or frequency of senescence induction appear to be primarily attributable to anatomical and histological differences, including variations in epidermal thickness.

In principle, we could conduct additional experiments comparing injury responses in light-exposed versus light-protected skin regions (e.g., dorsal/back vs. ventral/belly skin of pigs) or even pre-expose the skin to UV light to assess whether such treatment alone can induce rapid-onset senescence or influence the kinetics and frequency of senescence induction upon injury. These experiments would require obtaining new ethical approval (Tierversuchsantrag; TVA) and developing corresponding models, therefore this would take us a long time to generate reliable results on. Following the editors' advice to prioritize experimental efforts addressing points 5 and 6, we have not directly pursued these experimental efforts at this stage. However, we intend to initiate the necessary procedures to perform these experiments, which will likely be included in our follow-up work. In the meantime, we have added a dedicated section discussing this aspect in the revised manuscript:

"Not only in relation to various tissue types, but the induction of rapid-onset senescence upon mechanical injury is likely to also differ mechanistically from senescence triggered by damage that does not directly breach the tissue integrity, such as UV or γ-irradiation, chemotherapeutics, or chemical agents such as DMBA/TPA, all of which primarily act through DNA damage or oxidative stress pathways (PMID: 26106138). Likewise, it is unknown whether conditions that are known to modulate the basal level of senescence-related machinery, including levels of *Ckdn1a* mRNA, such as aging or sun exposure (contrasted by light-protected skin) could impact on the rate or efficiency of the rapid-onset senescence induction in situation of tissue breach such as a mechanical injury. Future work comparing mechanically induced versus irradiation- or drug-evoked senescence, including in light-deprived skin, will help to clarify how environmental exposure influences the skin's senescence landscape.".

Likewise, the authors did not consider to test damage-accumulated old skin (leading to "actinic keratosis" in humans) vs. young skin.

Thank you. This is indeed an interesting issue that has also been on our minds for quite some time. While we are still in the process of generating colonies of aged mice to conduct a set of studies focused specifically on aged skin, we have recently gained access to several aged mice. To address the reviewer's comment, we performed experiments, imaging, and quantification of p21 in injured skin of both aged and young animals. Although the study is somewhat underpowered, it shows a trend (p=0.0977) suggesting that aged skin exhibits a reduced capacity to mount rapid-onset senescence, with fewer senescent cells induced in 6-hour wounds of aged skin compared to those of young skin (see the figure below). We consider these data too preliminary and somewhat beyond the scope of the current manuscript, but we intend to explore this observation further in our upcoming research project.

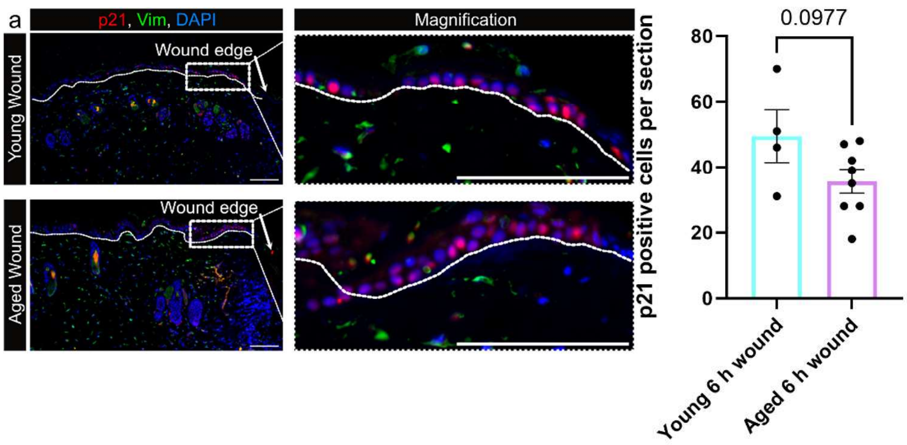

a) Representative images of staining for p21 (red) in samples of murine 6 h wounds collected from young and aged animals. DNA (blue) was stained with DAPI. The epidermis is outlined in white. **b**) Quantification of the fraction of p21^{+} keratinocytes. Data are from n = 4-8 mice per group; mean ± SEM are plotted. An unpaired t test was used. Scale bars are 100 µm.

3. Data on the secreted factor epigen appear rather isolated and non-central to the manuscript. Similarly, the Perilipin-related lipid droplets are a side story of limited value.

We thank the reviewer for this thoughtful comment. We agree that the data on epigen and the Perilipin-related lipid droplets are not the central focus of our manuscript. However, we believe they provide useful complementary insights into the broader biological context of our findings. Specifically, the inclusion of epigen data highlights the possible involvement of secreted factors downstream of our main pathway, while the Perilipin-related results support is used as a further validation of cellular senescence induction, as this marker has been associated with cellular senescence in vivo earlier (PMID: 28608850, 30612898, 39121846). That said, we have revised the text to clarify their supporting rather than central role and to avoid overemphasizing these aspects.

4. The CIP1 (p21) promoter-driven Cre:ER crossed to the Rosa26-LSL-dtTomato strain is, in principle, a powerful tool, but the information provided about timing of the tamoxifen (Tam) pulse-chase administration – e.g. "at the time of injury" – is not clear enough to allow readers to understand the biological consequences, i.e. how quick a p21 promoter-driven Cre:ER product would be available for Tam to unleash a red-fluorescent read-out, especially in light of the author-undervalued aspect that p21 transcripts (hence, the Cre:ER transcript as well?) are pre-elevated in skin keratinocytes…
We fully agree with the reviewer that clarifying the rate of tdTomato expression following Tamoxifen injection in our p21-tracker (p21-CreER-R26-tdTomato) model is of great importance. To address this point, we sacrificed all currently available p21-tracker mice at three time points after Tamoxifen (20 mg/kg) injection: 30 minutes, 6 hours, and 24 hours. In addition, excision wounds were induced in the mice 6 hours before sacrifice. We also included two wild-type (WT) mice, which were likewise injected with Tamoxifen at 24 hours and wounded 6 hours before sacrifice. Regarding tdTomato mRNA levels, we found that 6 hours was insufficient to observe transcript induction in the wounds. Induction became evident at 24 hours post-Tamoxifen injection, indicating that effective transgene activation occurs between 6 and 24 hours. We were somewhat disappointed that the overall induction was modest (approximately 5–10-fold over background levels), which may relate to the fact that 6 hours after injury is not yet sufficient to induce Cdkn1a transcription, and consequently the tdTomato transgene. Consistent with this, another experiment performed for this revision showed that it takes approximately 8 hours to achieve robust Cdkn1a expression in wounds.

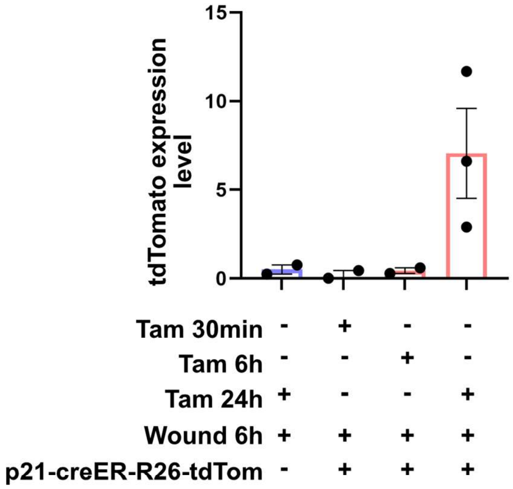

Results of RT-qPCR for tdTomato expression in 6 h wounds upon Tamoxifen (20mg/kg) injection to p21-creER-R26-tdTomato (red bars) and WT (blue bar) mice. Tamoxifen was injected at 30 min, 6 h and 24 h to creER-R26-tdTomato and at 24 h to WT mice.

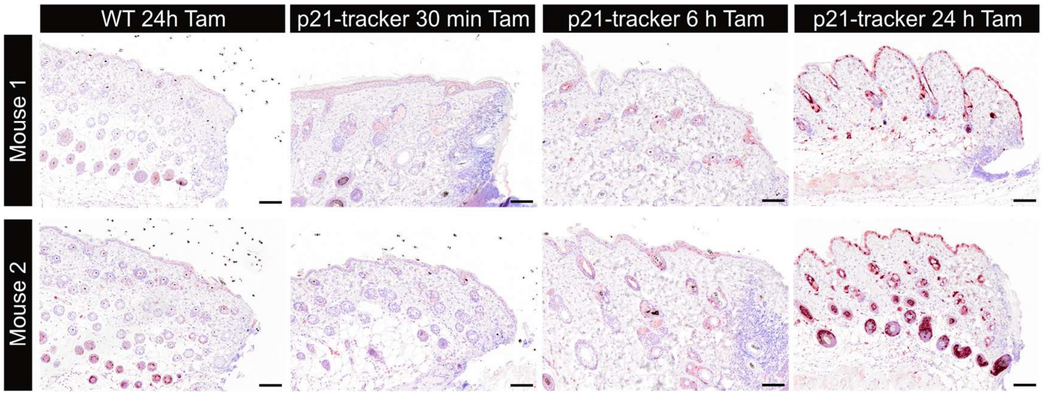

The follow-up histology using tdTomato staining confirmed that, although some nonspecific signal is present in the 30 min– and 6 h–Tam-treated as well as in WT mice, a prominent signal is observed only in p21-tracker mice treated with Tam for 24 h. Overall, these experiments suggest that tdTom induction is specific to Tamoxifen and occurs after 6 h and before 24h of treatment.

5. The idea of stress-driven, pre-elevated p21 transcripts in the nucleus of light-exposed keratinocytes to prime for rapid-onset senescence is interesting. However, why these transcript levels do not further increase upon acutely inflicted tissue injury remains unexplained.

We are grateful to the Reviewer for highlighting this seemingly contradictory pattern. Indeed, we uphold both claims: that the rapid-onset induction of cellular senescence is transcription-independent, and yet the expression of Cdkn1a increases at a later time point after injury. This can be explained by the fact that the rapid induction of senescence appears to rely primarily on pre-existing Cdkn1a transcripts. However, since mRNA has a limited lifespan and its usage is elevated under these conditions, Cdkn1a expression subsequently rises to sustain high levels of p21 protein in senescent cells throughout the healing process. We have demonstrated by RT-qPCR that there is no increase in Cdkn1a expression in the skin within the first few hours after injury (Extended Data Figure 9g-h). To address the Reviewer's request for further investigation, we have also performed RNA in situ hybridization (RNA-ISH) before injury and at 1.5 h, 8 and 10 h post-injury, observing no significant difference for 1.5h, but a prominent upregulation of Cdkn1a at 8 h and 10 h. This data is presented below, and in response to one of the minor comments, it has been added to the manuscript as not to Extended Data, but instead to one of the main figures, Figure 5d-e.

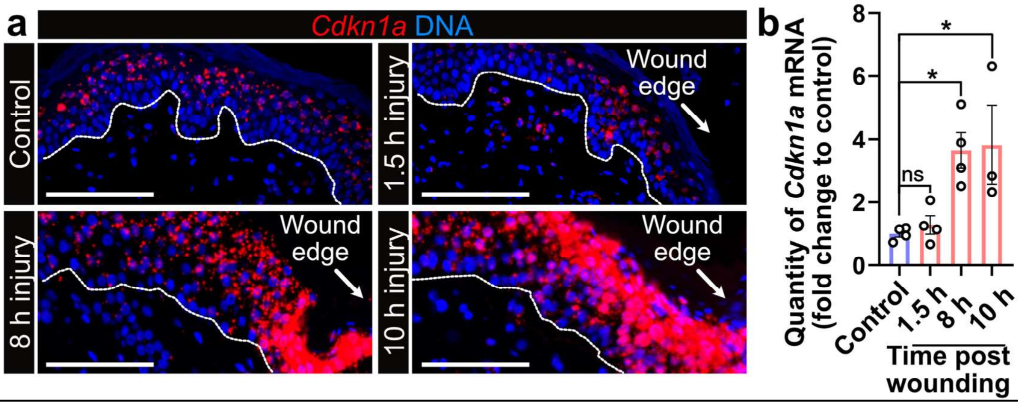

(**a**) Representative images of RNA-ISH staining against *Cdkn1a* (red) in samples from porcine skin unwounded or wounded and collected at 1.5 h, 8 h and 10 h after injury.

(**b**) Quantification of the expression of *Cdkn1a* in keratinocytes after 1.5 h, 8 h and 10 h post injury and normalized to the average of the control skin. Mean ± SEM plotted. One-way ANOVA with Dunnett's post-hoc test was used. n = 3 - 4 pigs. Scale bars show 100 µm.

The authors report higher p53 protein levels and elevated p21 transcript levels in the pre-injury normal skin, and argue that binding of the p21 transcript to certain RNA-binding proteins prevents its translation, ok. But what happens to p53 levels as the p21-driving transcription factor during injury, why is p53 protein found low in the wound? Why are no other wound-associated mechanisms – namely FoxO/TGF-beta-Smads – employed to drive p21 transcription (Seonae et al., Cell 2004; Ponugoti et al., JCB 2013)?
And, while there might be a small fraction of p21 protein expression to be controlled via mTOR-governed translation (and, hence, amenable to mTOR inhibition [Beuvink et al., Cell 2005]), other but transcription-dependent mechanisms might apply to p21 regulation upon mTOR inhibition: mTOR inhibition will reduce feedback inhibition of PI3K activity, allowing AKT to inactivate FoxOs, which, together with Smads (see above) drive p21 translation.

Thank you for these hypotheses and we agree that these pathways could be potential contributors to the rapid-onset senescence. Specifically, we investigated p53 levels and signaling in our experimental setup for wounding-induced rapid onset cellular senescence. Using our *ex vivo* wounding methodology, we tested a range of compounds to assess potential upstream pathways responsible for the rapid induction of p21-positive senescent cells following injury. Our initial hypothesis was that DNA damage and the DNA damage response (DDR), both well-established triggers of senescence (PMID: 39121846, 32212823, 24449267, 18574463), might be involved. To test this, we conducted an additional set of experiments, which yet revealed that inhibition of p53 (using Pifithrin-α; 200 μM) or ATM (using KU55933; 15 μM) does not prevent the induction of p21-positive cells at the injury site. These findings are presented below and in a newly added supplementary figure included in this revision (Extended Data Figure 10).

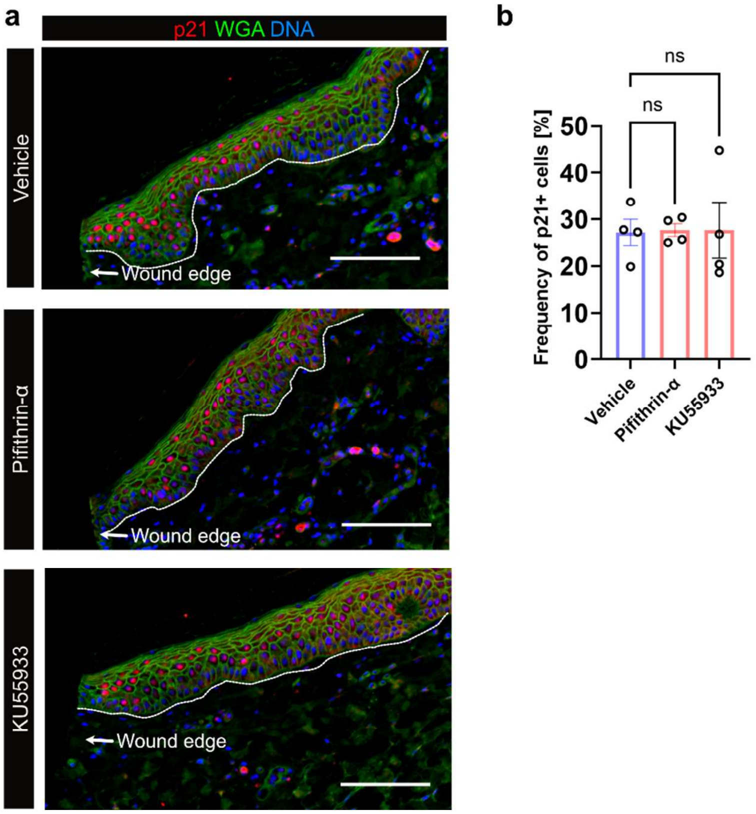

a) Representative images of staining for p21 (red) in samples from porcine skin *ex vivo*, wounded and treated for 1.5 h with vehicle (DMSO in PBS), p53 inhibitor (Pifithrin-α; 200 μM), or ATM inhibitor (KU55933; 15 μM). DNA (blue) was stained with DAPI, and cell membranes are outlined using WGA (green). The epidermis is outlined in white. **b**) Quantification of the fraction of $p21^+$ keratinocytes. Data are from n = 4 pigs; mean ± SEM are plotted. One-way ANOVA with post-hoc Dunnett's test was used for statistical analysis. For all images, scale bars represent 100 μm.

Additionally, we investigated whether p53-positive cells overlap with p21-positive cells. To this end, we used a p53 antibody (Abcam; ab13144) and performed a series of stainings to determine whether the observed p21-positive cells also express p53. Interestingly, we found that while p21-positive cells do not exhibit detectable levels of p53 (Fig. below, lower panel), other cells located farther from the injury site do. In particular, cells of the sebaceous glands, which produce sebum, a fatty substance that protects the skin, appear to express high levels of p53 in the absence of p21 (middle panel). This observation could serve as the basis for a follow-up study, and we would be happy to collaborate on exploring the role of $p53^{+}/p21^{-}$ cells in skin physiology. These data are included in Extended Data Figure 10.

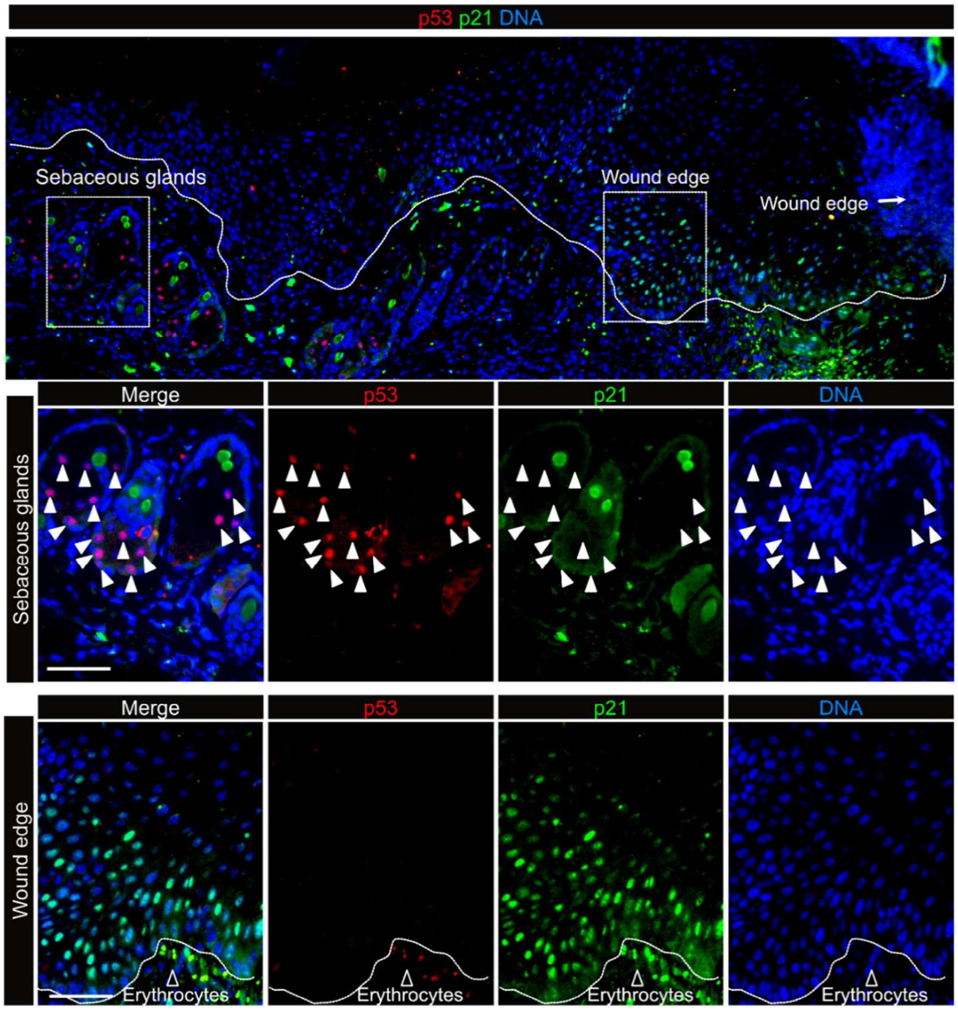

Representative images of staining for p21 (green), p53 (red), and DNA (blue) in samples from mouse wounded skin. The upper panel shows a low-magnification view of the peri-wound region. The middle panel shows a magnified view of sebaceous glands. $p53^{+}$ cells are marked with white arrows. The bottom panel shows the epidermis at the edge of the injury. $p21^{+}$ cells are not marked as they are too numerous. A hollow arrow indicates erythrocytes that are autofluorescent and visible in both green and red channels. The epidermis is outlined in white. For all images, scale bars represent 50 µm.

Moreover, to provide evidence on the relevance of Smad pathways in the rapid-onset senescence phenotype, we purchased an antibody against p-Smad2 (ab280888). Fortunately, the antibody has proven to be very reliable in visualizing p-Smad2-positive cells, which we observed in high quantities in murine wounds 3, 7 and 12 days after injury was performed.

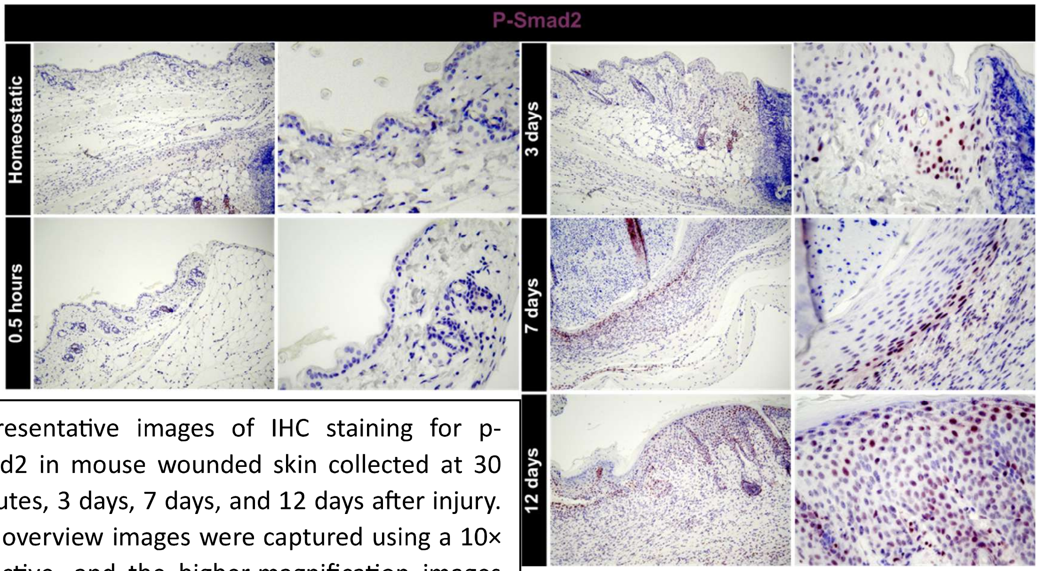

Representative images of IHC staining for p-Smad2 in mouse wounded skin collected at 30 minutes, 3 days, 7 days, and 12 days after injury. The overview images were captured using a 10× objective, and the higher-magnification images on the right were captured using a 40× objective.

Interestingly, we observed that cells (keratinocytes) that are positive for p21 and almost always also co-positive for p-Smad2 (see below).

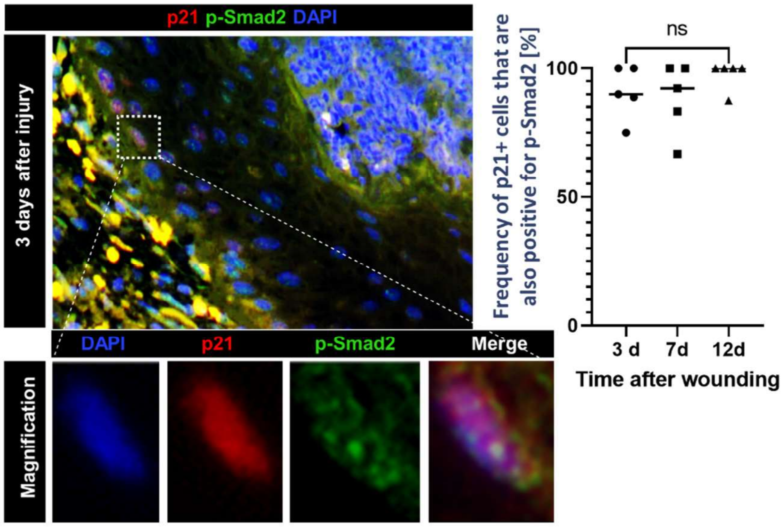

(**left**)Representative image of IHF co-staining for p-Smad2 and p21 in mouse wounded skin collected at 3 days after injury.
(**right**) Quantification of keratinocytes positive for p21 that are co-positive for p-Smad2 3, 7 and 12 days post wounding. N=5 mice per group and one-way ANOVA with Tukey's test was used.

Yet, after testing both porcine (see figure below) and murine (see the figure with IHC images above) samples collected within minutes to hours after injury, we did not detect any reliably p-Smad2–positive cells. Therefore, our current conclusion is that while the Smad pathway is likely involved in the maintenance of senescence or in changes occurring within senescent cells over time, we currently have no evidence supporting its role in the induction of rapid-onset senescence following injury.

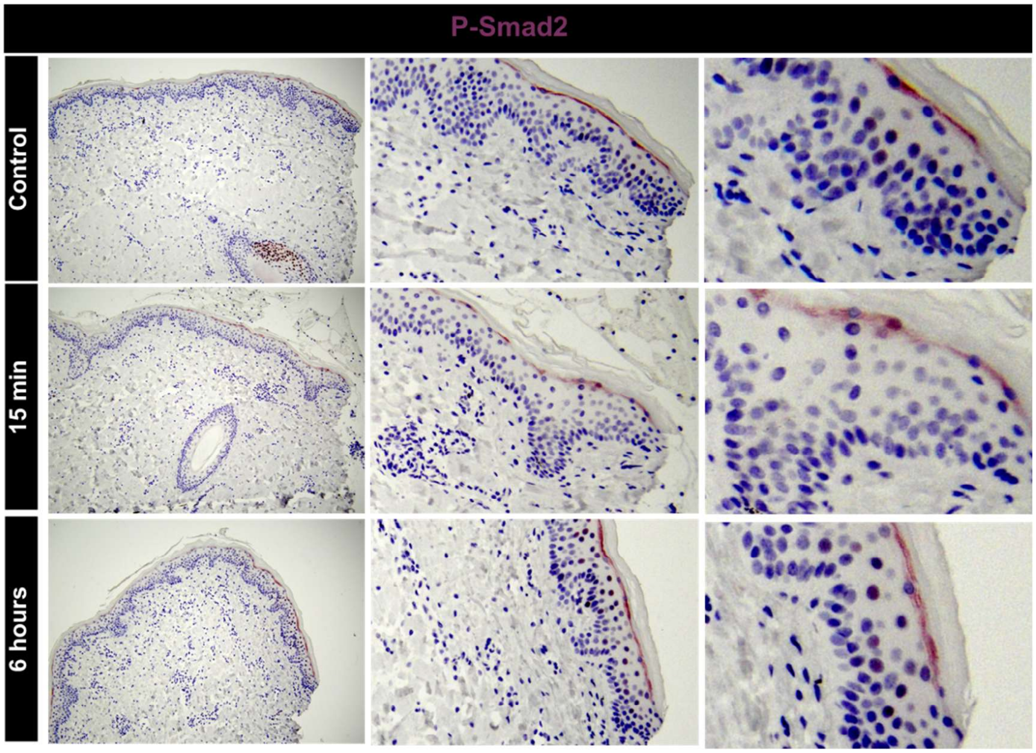

Representative images of IHC staining for p-Smad2 in porcine wounded skin collected at 15 minutes and 6 hours after injury as well as in control skin. The overview images were captured using a 10× objective, and the magnification images on the right were captured using a 20x and 40× objective, respectively.

Finally, we used the proteomics experiment to test protein levels of key components involved in the FoxO/TGF-beta/Smads signaling. We searched the KEGG database to obtain the gene lists for the FoxO signaling pathway (hsa04068) and the TGF-beta signaling pathway (hsa04350). Genes annotated as participating in these pathways were compiled and compared against entries in our (bulk) proteomics results (of 15 min and 4 h excision injury response in porcine skin) to identify which proteins were present. Genes not found in the proteomics database were noted but excluded from visualization. Using the subset of pathway genes, of which protein products were detected in our proteomics experiment, we generated a heatmap and highlighted proteins statistically significant based on the adjusted p-values available in the proteomics results, applying a significance threshold of 0.05 for the adjusted p-value (see below). The results did not provide a very consistent pattern. Out of 73 interrogated proteins involved in FoxO/TGF-beta/Smads signaling, we found 10 proteins being upregulated and 8 proteins being downregulated at 4h upon injury. 55 proteins, including Foxo3 and majority of Smad proteins were not significantly changed (see the figure as below). Together with the histological results of the timepoints minutes, hours, and days after injuries in pigs and mice, these experiments did not provide prominent evidence that FoxO/TGF-beta/Smads signaling is leading senescence induction upon injury. Nonetheless, it is possible that more in-depth approaches, such as phospho-proteomics or pharmacologic or genetic interventions, could provide more reliable evidence and we will be happy to perform such experiments in our follow-up work.

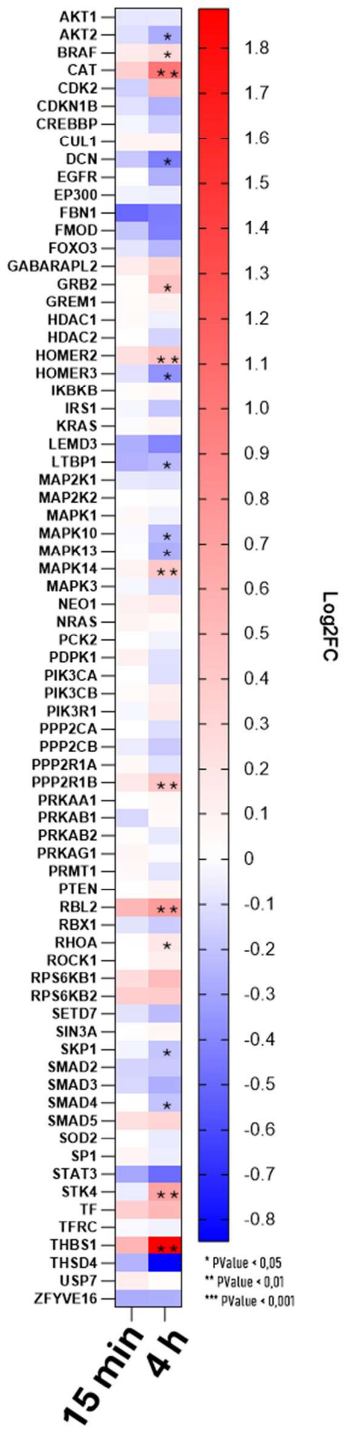

The heatmap shows protein levels associated with the FoxO/TGF-beta/Smads signaling pathway in porcine excision injuries at 15 minutes and 4 hours after wounding. Proteins with adjusted p-values meeting the significance threshold of 0.05 are marked with * ($p < 0.05$) and ** ($p < 0.01$).

These results on the FoxO/TGF-beta/Smads signaling pathway were not included in the resubmitted manuscript, as we found it challenging to explain them without making the text overly complex or distracting. Nonetheless, if the Reviewer would like us to include them, we would be happy to do it.

Moreover, the work is inconsistent to whether or not p21 transcripts are further induced upon injury (claimed stable, but found “amplified” in mRNA pull-down experiments).

We addressed this point above (see the response at the beginning of point 5), where we showed that although Cdkn1a mRNA is not increased at early stages of the wounding response (for example, 1.5 hours after wounding), when p21 protein is already elevated, it does increase at later time points. Specifically, at 8 and 10 hours after injury, Cdkn1a mRNA is significantly upregulated (Fig. 5d-e). Taken together, these results suggest that while the immediate, rapid-onset induction of senescence is indeed independent of transcription and relies on the pre-existing Cdkn1a mRNA, upregulation of Cdkn1a expression occurs at later time points (and is observed throughout the entire healing process), likely helping to maintain high levels of p21 protein and the stability of the cellular senescence phenotype.

*The word “*amplified*” in that context was referring to being amplified by RT-qPCR, not increased in quantity. We corrected it to “present”.*

6. How p21-specific the SRSF binding to mRNA really is would need to be shown; conversely, the reduced detectability of SRSF3 and hnRNP proteins by mRNA pull-down may be due to injury-related dissociation from the transcript, or by lowered protein expression for any other reason. The authors assume that this will enable the mRNA’s export to the cytoplasm, but nuclear translation (Reid and Nicchitta, JCB 2012) of p21 as an alternative and very rapid stress-related alternative is not even discussed.

We agree with the reviewer that it is a very relevant point. One core experiment we performed specifically this point (but in addition also point about the FoxO/TGF-beta/Smads signaling) is the bulk proteomics experiment. This experiment is described in detail above (in the summary section at the beginning of the response letter) and in the manuscript, but briefly, we collected porcine control skin as well as excision wounds at 15 min and 4 hours upon injury and conducted proteomics.

Thus, firstly, we compared the results of the (bulk) proteomics with the results of the Cdkn1a mRNA pulldown proteomics.
Among the proteins upregulated on Cdkn1a mRNA, none showed significant upregulation at the bulk level, and several were downregulated (see below and Extended Data Fig. 13a), highlighting the specificity of the injury-induced protein binding to Cdkn1a mRNA. Similarly, among 71 proteins downregulated on Cdkn1a mRNA, 2 were also upregulated and 15 were downregulated at the bulk level, yet minimally (see below and Extended Data Fig. 13b). For example, while bulk levels of SRSF3 and hnRNPK decreased by approximately 20%, their quantity on Cdkn1a mRNA was reduced by ~80% and ~65%, respectively, indicating that most changes occurring on Cdkn1a mRNA are specific to the transcript and not primarily driven by global protein expression changes.

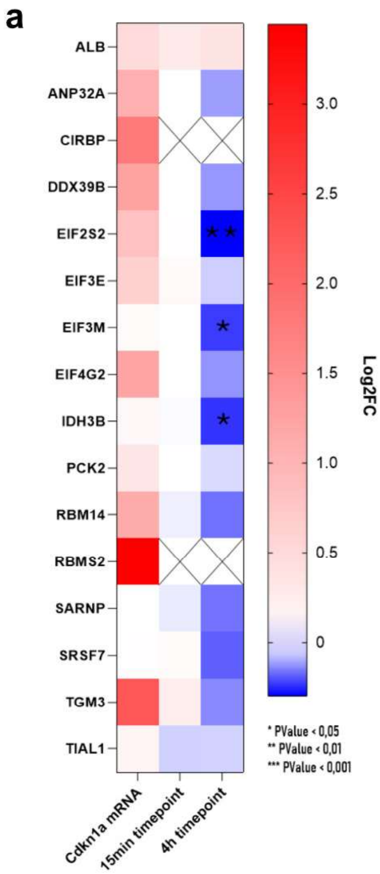

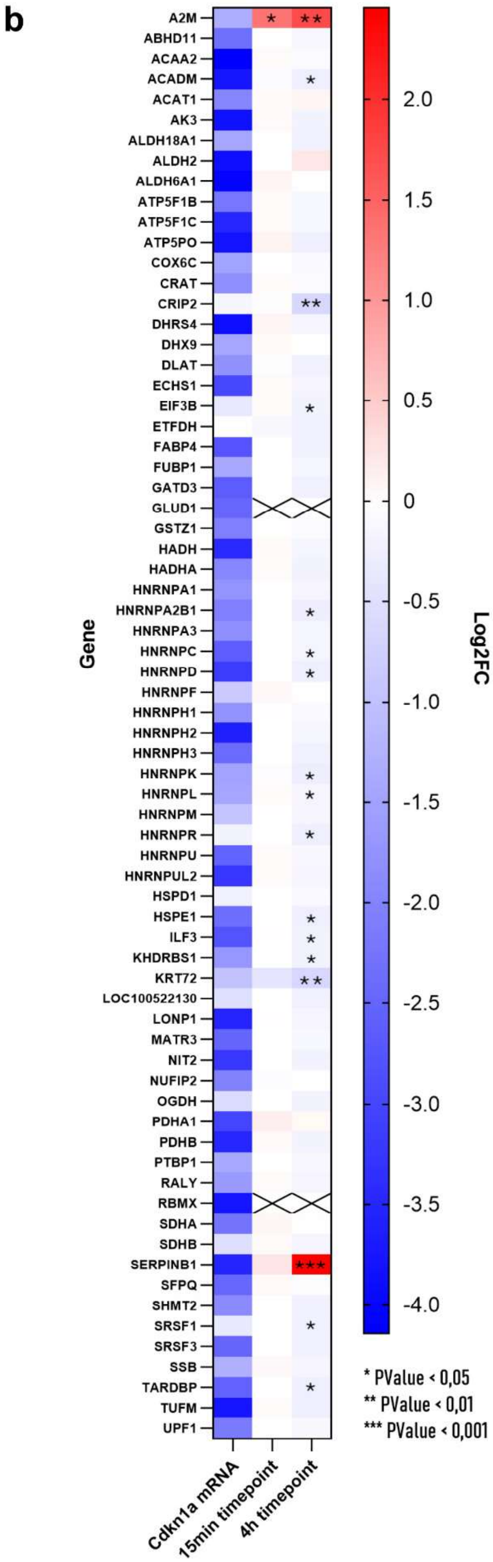

(**a–b**) Heatmaps showing log2 fold changes (log2FC) from the proteomic analysis of the Cdkn1a mRNA pulldown (left column) and bulk proteomics at 15 minutes (middle column) and 4 hours (right column) post-excision injury in porcine skin. Heatmaps display proteins (**a**) upregulated or (**b**) downregulated in association with Cdkn1a mRNA in porcine excision wounds and microneedling experiments. Proteins not found in the (bulk) proteomics analyses are crossed out. *, ** and *** indicate adjusted p value below 0,05, 0,01 and 0,001 respectively.

Secondly, to further confirm the specificity of the interaction, we have performed a comprehensive analysis integrating multiple lines of bioinformatic evidence. We utilized the ATtRACT database, which experimentally validated motifs of RNA-binding protein targets. Our analysis indicated strong predicted binding sites for both SRSF3 and hnRNPA1 within the 3' UTR of the Cdkn1a transcript (see parts a and b of the figure below). Next, leveraging multi-species orthologs obtained from NCBI, we examined the conservation of these predicted binding sites across evolutionarily distant species (see part b of the figure below). This conservation suggests functional relevance for these protein-RNA interactions in general, including potentially in contexts like wound healing. Crucially, to directly link these bioinformatic predictions to experimental data, we incorporated SRSF3 iCLIP peak data from a relevant published study (GEO dataset GSE123002; PMID: 31145021). Our analysis of this dataset confirms that SRSF3 indeed binds within the predicted and conserved region in the 3' UTR of Cdkn1a (see part c of the figure below). These analyses collectively provide robust bioinformatic validation for the specificity of SRSF3 and hnRNPA1 binding to the Cdkn1a mRNA.

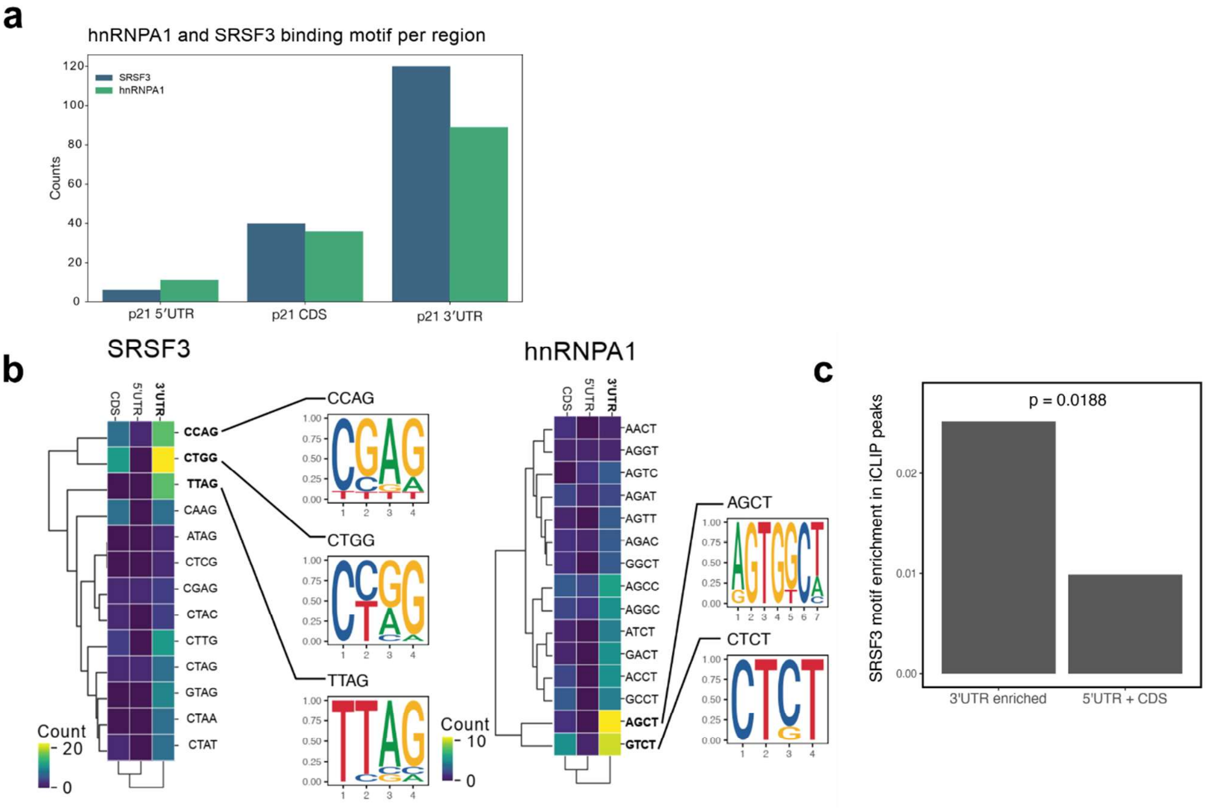

(**a**) Predicted RBP motif distribution across *Cdkn1a* transcript regions.

(**b**) Sub-motif enrichment patterns for SRSF3 and hnRNPA1 across *Cdkn1a* transcript regions. Clustered heatmaps showing the distribution of individual SRSF3 (Right) and hnRNPA1 (left) sub-motifs across the 5'UTR, coding sequence (CDS), and 3'UTR of *Cdkn1a*. Evolutionary conservation of *Cdkn1a* motif sequences across mammals are shown in the DNA logo plots.

(**c**) Bar graph enrichment SRSF3 iCLIP enrichment and SRSF3 motif density across *Cdkn1a*. 3'UTR motifs significantly higher than in background region 5'UTR and CDS of *Cdkn1a*. Enrichment was tested using Fisher's exact test.

Thirdly, to provide additional evidence for the specificity of the SRSF3–Cdkn1a mRNA interaction and its dissociation upon injury, we conducted RNA immunoprecipitation followed by quantitative PCR (RIP-qPCR). This experiment was both the most challenging and high-risk, yet also the most directly relevant to the reviewer's request to verify the binding capacity of SRSF3 to Cdkn1a mRNA. Given the limited time frame, obtaining all necessary samples and reagents for this several-week procedure proved extremely demanding. Nevertheless, we succeeded in organizing an in vivo pig experiment, from which we collected 198 samples of excision wounds and 198 samples of control skin, comparable to the ChIRP-MS setup and estimated to contain roughly 500,000,000 p21^{+} cells per condition.

We immunoprecipitated SRSF3 crosslinked to RNA from both wounded and unwounded skin (see the figure below (a) and Extended Data Fig. 13c), reverse-transcribed the recovered RNA into cDNA, and performed RT-qPCR analysis. To validate the efficiency and specificity of the RNA immunoprecipitation, western blot (WB) images showing 5% of the Protein A bead fraction from each RIP condition were analyzed. Samples included Control + anti-SRSF3, Wound + anti-SRSF3, and the corresponding No-antibody (NoAb) controls processed in parallel. In addition, a Rabbit IgG control was included on the blot to exclude nonspecific bands. The flow-through (FT) fractions collected after bead incubation were analyzed for Control and Wound lysates to document depletion of SRSF3 from the supernatant upon immunoprecipitation. Despite initial concerns that the available material might be insufficient for SRSF3 immunoprecipitation and reliable Cdkn1a detection, all controls confirmed the validity of the experiment. The results demonstrated that, 1.5 hours after injury, SRSF3 binds approximately 50% less Cdkn1a mRNA compared with healthy control skin (see the figure below (b) and Fig. 6g). These data confirm our hypothesis and further substantiate the specificity of the SRSF3–Cdkn1a mRNA interaction, supporting the proposed mechanism of Cdkn1a mRNA dissociating from SRSF3 upon injury. Detailed methodology is provided in the Extended Data document, and the raw western blot data are included in the file "Source Data – Unprocessed Western blots and gels."

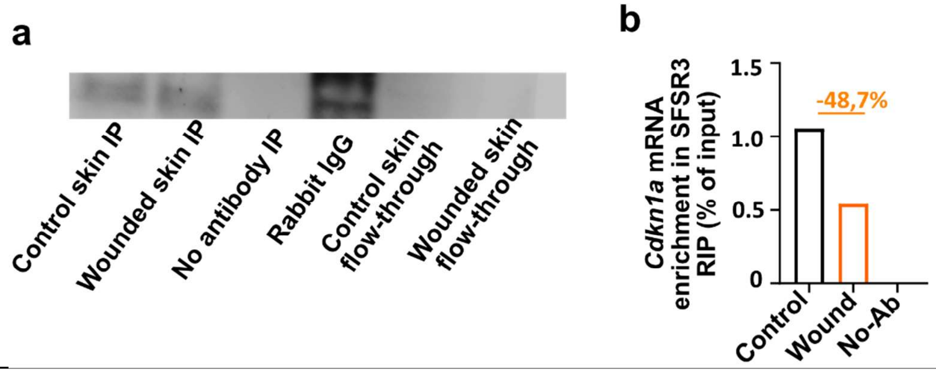

SRSF3 binds less *Cdkn1a* mRNA after injury.

(**a**) WB showing validation of SRSF3 RNA IP in wounded and control porcine skin, including anti-SRSF3, No-antibody, and Rabbit IgG controls, as well as flow-through fractions demonstrating SRSF3 depletion.

(**b**) Results of RNA IP followed by quantitative PCR (RIP-qPCR) analysis of wounded and unwounded porcine skin showing the change in the amount of Cdkn1a mRNA bound to SRSF3 before ("Control") and 1.5 hours after excision injury ("Wound"). "No-Ab" is RIP-qPCR when no SRSF3 antibody was used.

*Finally, indeed, involvement of nuclear translation is not negligible, and accordingly, we added a dedicated section to the discussion part: "*While our data indicate that reduced binding of SRSFs and hnRNPs to *Cdkn1a* mRNA promotes its nuclear export for cytoplasmic translation,

nuclear translation (PMID: 22472436) represents a compelling complementary mechanism that may enable rapid p21 induction under stress. This process allows select mRNAs to engage nuclear ribosomes directly, bypassing export delays through nuclear pores and supporting swift protein synthesis. Future studies employing isolated nuclear pulldowns will be crucial to delineate the relative contributions of these mechanisms and to confirm the binding specificity of the *Cdkn1a* mRNA–binding proteins identified in this study.*".*

Minor concerns

1. The writing style requires revision for clarity. For instance, "…healing process. One step in this process is the formation of the p-rpS6-zone, which occurs within mere minutes after wounding…" refers to a previous finding of the last author, but remains without any explanation. What that zone is, how big it is, etc. is important for NCB's readership, and is certainly not common knowledge in the scientific community. As another example, "Mechanistically, Cdkn1a transcripts remove proteins, …" is scientifically simply wrong. Or the entire sub-paragraph starting with "In all our tracking experiments we observed a single instance of a cluster tdTom+ keratinocytes that was mostly negative… …demonstrate that injury-induced senescent cells are removed after wound closure via distinct, cell-type specific mechanisms" is not only impossible to follow at the outset, but presents a conclusion at the end for which the underlying data are missing.

Thank you for bringing this issue up, and we now made changes in the text to correct any issues.

Regarding "…healing process. One step in this process is the formation of the p-rpS6-zone, which occurs within mere minutes after wounding…*", to simplify the introduction we now omit the information about the p-prS6 zone and rephrase it simply: "*Our recent findings indicate that signaling pathways become active within minutes following skin injury and exhibit spatiotemporal control that shapes the healing process*".*

*We also corrected the mistake in the statement "*Mechanistically, *Cdkn1a* transcripts remove proteins…*" and changed the sentence to: "*Mechanistically, *Cdkn1a* transcripts lose proteins that are known to restrict nuclear export, which in turn permits interaction with translation initiation factors and promotes translation*".*

*The sub-paragraph starting "*In all our tracking experiments we observed*" was brought earlier to address one of the comments of the reviewers about whether we observed any instances of the tdTomato-positive cells in p21-creER-R26-tdTomato mice that were yet negative for p21. As we observed a single instance of keratinocytes showing this pattern, this is what we considered important to report. Now, we have expanded and clarified this part so that it is easier for follow and understand: "*In all our tdTom-based tracing experiments of p21-expressing cells in p21-creER-R26-tdTomato mice, we observed only a single instance of a cluster of tdTom$^+$ keratinocytes that were largely negative for p21. In this case, the tdTom$^+$ keratinocytes originated from the bulb of a hair follicle (Extended Data Fig. 8p), suggesting that some cells may escape senescence, or express insufficient levels of p21 to fully enter it, although this appears to be exceedingly rare. Based on our observations that, upon completion of healing, tdTom$^+$ keratinocytes are eliminated through epidermal shedding and tdTom$^+$ fibroblasts become co-positive for apoptotic markers, we conclude that injury-induced senescent cells are cleared after wound closure via distinct, cell-type-specific mechanisms.*"*

Finally, we reviewed the text to identify any remaining errors and overly complex sentences to improve overall readability.

2. Often (especially in the legends) the authors miss to clearly state whether they talk about protein or transcript expression – although this distinction is central.

Thank you for catching this. We have updated all figure legends and the text to consistently label the transcript as Cdkn1a and the protein as p21. There were several corrections of this sort made for the Figures 1, 2 and 5. We also added the statement to the introduction section clarifying this: "… p21$^{CIP1/WAF1}$ protein (hereafter referred to as p21) that is encoded by *Cdkn1a* gene (whose transcript/mRNA is also denoted as *Cdkn1a* mRNA) …*".*

Moreover, sometimes, only data on wounded, not non-wounded material is shown, or cell numbers cannot be compared (e.g. p21+ cells per mm2 vs. frequency of p21+ cells).

While we are not entirely certain what the reviewer is referring to, we assume the comment concerns figure panels where, instead of displaying separate images for injured and control tissues, we show wound samples that also include non-wounded regions within the same section. In these cases, the absence of a phenotype in unwounded tissue is evident in areas located far from the wound edge. The scientific rationale for this approach is based on our current and previous findings (PMID: 37098351; 40516907), which demonstrate that wounding-associated phenotypes are restricted to a defined distance from the injury site. For instance, phosphorylation of rpS6 is confined to within approximately 1 mm of the wound edge (PMID: 37098351), whereas p21 induction occurs within about 400 µm (see Figure 3a and 3e). This layout allows us to conserve figure space, enabling the inclusion of more legible graphs and higher-resolution images. All biochemical and other non-spatial experiments were, of course, appropriately controlled using homeostatic skin collected well away from the injury site. We hope that this explanation clarifies our rationale. Nevertheless, we are happy to modify how these data are presented if the reviewer suggests specific figure panels that require adjustment.

*Regarding the comment that "*cell numbers cannot be compared (e.g., p21^{+} cells per mm^{2} vs. frequency of p21^{+} cells),*" we acknowledge that the reviewer may not have had access to the Extended Data materials, which would likely have clarified this point. In certain experiments, we were uncertain whether the data should be presented as absolute counts (e.g., number of cells per section) or normalized values (e.g., relative to the size of the injury or the wound edge affected by the injury), as the latter parameters change markedly over the course of tissue repair. To enhance reproducibility and transparency, we therefore reported both absolute and normalized values. Nonetheless, we will gladly revise the data presentation if the reviewer prefers a specific format or analysis.*

3. Why is p21/CIP1/CDKN1A transcript expression, although central to the manuscript, only shown in supplementary figures?

If we understand correctly, the reviewer is referring to Cdkn1a levels in rapid-onset senescence. These data were originally presented in Extended Data Figure 11 due to space limitations in the main figures and because no significant changes in Cdkn1a mRNA levels were observed within the first minutes to hours after injury. We have now expanded this dataset to include the 8 h and 10 h time points, which show a significant upregulation (albeit considerably later than the increase in p21 protein levels), and have moved the complete dataset to Figure 5d, e.

We thank the reviewer again for the valuable comments and constructive criticism and we believe that all these new experiments and analyses significantly strengthen the manuscript, and we hope that these revisions fully address the reviewer's concerns.

Response Letter

Reviewer #5:

Remarks to the Author:

This is now the re-revised version in reply to my (reviewer #5) comments and concerns. Following editorial guidance, the authors have undertaken major efforts to address many of my points, especially those listed as no. 5 and no. 6.

We are very grateful to the reviewer for the positive assessment of our revision.

I congratulate the authors for providing novel evidence that helps to clarify issues, although open questions remain throughout the manuscript. Despite all efforts undertaken to extract key molecular mechanisms in early wound healing, the process in focus is highly dynamic and certainly not perfectly synchronized. The story appears quite bold and complex, and is likely to stimulate intense discussions within the scientific community rather than being perceived as definite evidence.

We agree with the reviewer that this manuscript does not close the book on what rapid-onset senescence is or on the role of these cells in animal physiology. We are very excited about the follow-up research and future findings on this topic by the scientific community.

As a "late comer" to this already extensive review process and the additional experiments enforced thereby, I find this work now, in principle, mature enough and of sufficient quality to be published in NCB.

Thank you! We are thrilled to proceed with the publication of these findings in Nature Cell Biology.

However, I strongly recommend another round of internal (and, perhaps, external) redacting of the data composition and phrasing (which should not involve expert reviewers anymore...) - there is still room for a more succinct, clear and sharpened presentation, which is essential for such data-dense work not to lose its readership in the middle of the main text, distracted by an endless load of supplementary figure material.

Thank you for these suggestions. We also received numerous comments from the editors, totaling about 15 pages, with detailed recommendations on how to revise the manuscript to make it more accessible to readers. Among other changes, one-third of the extended data figures were moved to the supplementary materials, and the manuscript was shortened accordingly. We are hopeful that these revisions have helped us strike an appropriate balance between providing sufficient evidence to support the reliability of our findings and ensuring that the presentation remains accessible and engaging for readers.

This also includes or starts with the abstract, which could be much more scientific, i.e. referring to specific experimental cause-consequence findings (unlike the "...wounding causes cells to become senescent..." style), and skipping information of no direct value (such as "..., including epigen,...") for outlining the big picture.

*To accommodate this suggestion we changed the abstract to: "*Cellular senescence contributes to tissue repair, tumor suppression, and aging. We identify a rapid, transcription-independent senescence response in skin following injury. Within minutes to hours after wounding, neighboring skin cells display hallmark features of senescence. This response involves the utilization of pre-existing Cdkn1a mRNA through the shedding of nuclear export inhibitors, which enables Cdkn1a transcript translation and rapid p21 protein accumulation. These cells undergo stable cell-cycle arrest and secrete pro-migratory and pro-inflammatory factors that promote tissue repair, including re-epithelialization. Experimental suppression of this rapid senescence, either genetically or pharmacologically, markedly delayed wound closure, whereas inhibition during later repair phases had no effect. Our findings establish rapid-onset senescence as a mechanistic requirement for efficient tissue regeneration.*".*

Remarks on Protocol(s):

I did not review the methods in detail.

The manuscript, along with all extended data and supplementary materials, has been updated to align with the journal's recommendations and requirements.
